# PTPN2 phosphatase deletion in T cells promotes anti‐tumour immunity and CAR T‐cell efficacy in solid tumours

**DOI:** 10.15252/embj.2019103637

**Published:** 2019-12-05

**Authors:** Florian Wiede, Kun‐Hui Lu, Xin Du, Shuwei Liang, Katharina Hochheiser, Garron T Dodd, Pei K Goh, Conor Kearney, Deborah Meyran, Paul A Beavis, Melissa A Henderson, Simone L Park, Jason Waithman, Sheng Zhang, Zhong‐Yin Zhang, Jane Oliaro, Thomas Gebhardt, Phillip K Darcy, Tony Tiganis

**Affiliations:** ^1^ Monash Biomedicine Discovery Institute Monash University Clayton Vic. Australia; ^2^ Department of Biochemistry and Molecular Biology Monash University Clayton Vic. Australia; ^3^ Peter MacCallum Cancer Centre Melbourne Vic. Australia; ^4^ Sir Peter MacCallum Department of Oncology The University of Melbourne Melbourne Vic. Australia; ^5^ Department of Microbiology and Immunology The University of Melbourne Melbourne Vic. Australia; ^6^ Peter Doherty Institute for Infection and Immunity Melbourne Vic. Australia; ^7^ Telethon Kids Institute University of Western Australia Perth WA Australia; ^8^ Department of Medicinal Chemistry and Molecular Pharmacology Institute for Drug Discovery Purdue University West Lafayette IN USA

**Keywords:** adoptive T‐cell therapy, CAR T cells, protein tyrosine phosphatase N2, STAT‐5 signalling, TCR signalling, Cancer, Immunology

## Abstract

Although adoptive T‐cell therapy has shown remarkable clinical efficacy in haematological malignancies, its success in combating solid tumours has been limited. Here, we report that PTPN2 deletion in T cells enhances cancer immunosurveillance and the efficacy of adoptively transferred tumour‐specific T cells. T‐cell‐specific PTPN2 deficiency prevented tumours forming in aged mice heterozygous for the tumour suppressor p53. Adoptive transfer of PTPN2‐deficient CD8^+^ T cells markedly repressed tumour formation in mice bearing mammary tumours. Moreover, PTPN2 deletion in T cells expressing a chimeric antigen receptor (CAR) specific for the oncoprotein HER‐2 increased the activation of the Src family kinase LCK and cytokine‐induced STAT‐5 signalling, thereby enhancing both CAR T‐cell activation and homing to CXCL9/10‐expressing tumours to eradicate HER‐2^+^ mammary tumours *in vivo*. Our findings define PTPN2 as a target for bolstering T‐cell‐mediated anti‐tumour immunity and CAR T‐cell therapy against solid tumours.

## Graphical abstract

Protein tyrosine phosphatase N2 inhibits tumour T‐cell infiltration and CAR T‐cell cytotoxicity and may thus be a therapeutic target to enhance solid‐tumour immunotherapy.

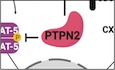

## Introduction

Tumours can avoid the immune system by co‐opting immune checkpoints to directly or indirectly inhibit the activation and function of cytotoxic CD8^+^ T cells (Pardoll, 2012; Ribas & Wolchok, 2018). In particular, the inflammatory tumour microenvironment can upregulate ligands for T‐cell inhibitory receptors such as programmed cell death protein‐1 (PD‐1) on tumour cells to inhibit T‐cell signalling and promote the tolerisation or exhaustion of T cells (Pardoll, 2012; Ribas & Wolchok, 2018). Immune checkpoint receptors, including PD‐1 and cytotoxic T‐lymphocyte antigen‐4 (CTLA‐4), can suppress the amplitude and/or duration of T‐cell responses by recruiting phosphatases to counteract the kinase signalling induced by the T‐cell receptor (TCR) and co‐stimulatory receptors such as CD28 on αβ T cells (Pardoll, 2012; Zappasodi *et al*, 2018).

Protein tyrosine phosphatase N2 (PTPN2) negatively regulates αβ TCR signalling by dephosphorylating and inactivating the most proximal tyrosine kinase in the TCR signalling cascade, the Src family kinase (SFK) LCK (van Vliet *et al*, 2005; Wiede *et al*, 2011). PTPN2 also antagonises cytokine signalling required for T‐cell function, homeostasis and differentiation by dephosphorylating and inactivating Janus‐activated kinase (JAK)‐1 and JAK‐3, and their target substrates signal transducer and activator of transcription (STAT)‐1, STAT‐3 and STAT‐5 in a cell context‐dependent manner (ten Hoeve *et al*, 2002; Simoncic *et al*, 2002; Wiede *et al*, 2017a,b). By dephosphorylating LCK, PTPN2 sets the threshold for productive TCR signalling and prevents overt responses to self‐antigen in the context of T‐cell homeostasis and antigen cross‐presentation to establish peripheral T‐cell tolerance (Wiede *et al*, 2014a,b). The importance of PTPN2 in T cells in immune tolerance is highlighted by the development of autoimmunity in aged T‐cell‐specific PTPN2‐deficient mice on an otherwise non‐autoimmune C57BL/6 background (Wiede *et al*, 2011), the systemic inflammation and autoimmunity evident when PTPN2 is deleted in the hematopoietic compartment of adult C57BL/6 mice (Wiede *et al*, 2017b) and the accelerated onset of type 1 diabetes in T‐cell‐specific PTPN2‐deficient mice on the autoimmune‐prone non‐obese diabetic (NOD) background (Wiede *et al*, 2019). In humans, *PTPN2* deficiency is accompanied by the development of type 1 diabetes, rheumatoid arthritis and Crohn's disease (Consortium, 2007, Long *et al*, 2011). The autoimmune phenotype of PTPN2‐deficient mice is reminiscent of that evident in mice in which the immune checkpoint receptors PD‐1 (Nishimura *et al*, 1999, 2001; Wang *et al*, 2005) or CTLA4 (Tivol *et al*, 1995; Waterhouse *et al*, 1995) have been deleted. Whole‐body PD‐1 deletion results in spontaneous lupus‐like autoimmunity in C57BL/6 mice (Nishimura *et al*, 1999) and accelerated type 1 diabetes onset in NOD mice (Wang *et al*, 2005), whereas CTLA4 deletion in C57BL/6 mice results in marked lymphoproliferation, autoreactivity and early lethality (Tivol *et al*, 1995; Waterhouse *et al*, 1995). Although PD‐1 and/or CTLA4 blockade can be accompanied by the development of immune‐related toxicities, antibodies targeting these receptors have nonetheless shown marked therapeutic efficacy in various tumours, including melanomas, non‐small‐cell lung carcinomas, renal cancers and Hodgkin lymphoma (Pardoll, 2012; Ribas & Wolchok, 2018). Accordingly, we sought to assess the role of PTPN2 in T‐cell‐mediated immunosurveillance and the impact of targeting PTPN2 on adoptive T‐cell immunotherapy. We especially focused on CAR T‐cell therapy, which has shown marked clinical efficacy in B‐cell acute lymphoblastic leukaemia (ALL), but has been largely ineffective in solid tumours (Grupp *et al*, 2013; Maude *et al*, 2014; Fesnak *et al*, 2016; Yong *et al*, 2017).

## Results

### PTPN2 deletion prevents tumour formation in p53^+/−^ mice

**Figure 1:**
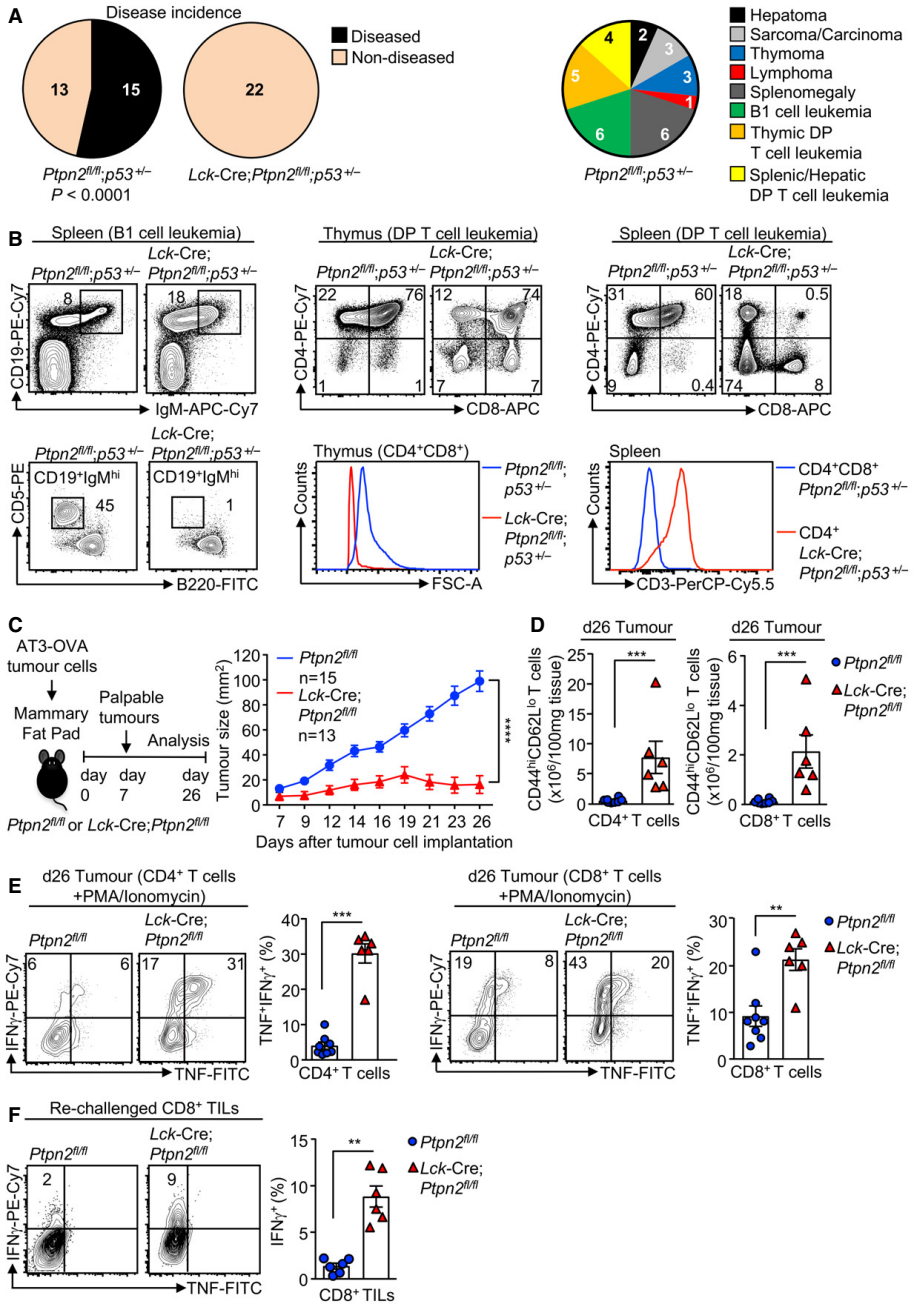
PTPN2 deletion in T cells increases tumour immunosurveillance A, B12‐month‐old *Ptpn2* ^*fl*/*fl*^;*p53* ^+/−^ and *Lck*‐Cre;*Ptpn2* ^*fl*/*f*^ *;p53* ^+/−^ mice were assessed for (A) disease and tumour incidence. (B) Lymphocyte subsets from 12‐month‐old *Ptpn2* ^*fl*/*fl*^;*p53* ^+/−^ and *Lck*‐Cre;*Ptpn2* ^*fl*/*f*^ *;p53* ^+/−^ mice were analysed by flow cytometry. Significance in (A) was determined using two‐sided Fisher's exact test.C–FAT‐3‐OVA mammary tumour cells were injected into the fourth inguinal mammary fat pads of female *Ptpn2* ^*fl*/*fl*^ and *Lck*‐Cre;*Ptpn2* ^*fl*/*fl*^ mice and (C) tumour growth monitored over 26 days. (D) At day 26 (d26), the numbers of activated tumour‐infiltrating lymphocytes (TILs) were determined. (E) The proportion of IFNγ^+^ versus IFNγ^+^TNF^+^ d26 TILs was determined by flow cytometry. (F) d26 TILs were incubated with AT‐3‐OVA tumour cells isolated from tumour‐bearing C57BL/6 mice, and the proportion of IFNγ^+^ T cells was determined.Data information: Representative flow cytometry profiles and results (means ± SEM) from at least two independent experiments are shown. In (C), significance was determined using 2‐way ANOVA test and in (D–F) significance determined using 2‐tailed Mann–Whitney *U*‐test. **P < 0.01, ***P < 0.001, ****P < 0.0001.

First we determined the impact of deleting PTPN2 in T cells on tumour formation in mice heterozygous for *p53*, the most commonly mutated tumour suppressor in the human genome (Hollstein *et al*, 1991). In humans, inheritance of one mutant allele of p53 results in a broad‐based cancer predisposition syndrome known as Li‐Fraumeni syndrome (Malkin *et al*, 1990). In mice, *p53* heterozygosity results in lymphomas and sarcomas, as well as lung adenocarcinomas and hepatomas in 44% of mice by 17 months of age with the majority of tumours exhibiting *p53* loss of heterozygosity (LOH) (Jacks, Jacks *et al*, 1994). We crossed control (*Ptpn2* ^*fl*/*fl*^) and T‐cell‐specific PTPN2‐null mice (*Lck*‐Cre;*Ptpn2* ^*fl*/*fl*^) onto the *p53* ^+/−^ background and aged the mice for 1 year. Upon necropsy 15/28 (54%), *Ptpn2* ^*fl*/*fl*^;*p53* ^+/−^ mice developed various tumours including thymomas, lymphomas, sarcomas, carcinomas and hepatomas (Fig 1A; Appendix Fig S1; Appendix Table EV1) as reported previously for *p53* heterozygous mice (Jacks *et al*, 1994). In addition, 6/28 mice exhibited splenomegaly accompanied by the accumulation of CD19^+^IgM^hi^CD5^hi^B220^int^ B1 cells consistent with the development of B‐cell leukaemias (Fig 1B), whereas CD3‐negative CD4^+^CD8^+^ double‐positive cells reminiscent of T‐cell leukaemic blasts (FSC‐A^hi^) were evident in the thymi or peripheral lymphoid organs of 5/28 mice (Fig 1A and B; Appendix Table EV1). Histological analysis revealed disorganised thymic, lymph node or splenic tissue architecture in diseased *Ptpn2* ^*fl*/*fl*^;*p53* ^+/−^ mice that were predominated by larger lymphoblasts consistent with the accumulation of pre‐leukaemic/leukaemic cells (Fig 1B, Appendix Fig S1). By contrast, no *Lck*‐Cre;*Ptpn2* ^*fl*/*fl*^;*p53* ^+/−^ mice (0/22) developed any overt tumours, splenomegaly or abnormal lymphocytic populations as assessed by gross morphology or flow cytometry and lymphoid organ tissue architecture was normal (Fig 1A and B, Appendix Fig S1, Appendix Table EV1). PTPN2 deficiency in T cells can result in inflammation/autoimmunity in aged C57BL/6 mice (Wiede *et al*, 2011). Accordingly, we determined whether PTPN2 deficiency might exacerbate inflammation in *p53* ^+/−^ mice. We found that inflammation, as assessed by measuring the pro‐inflammatory cytokines IL‐6, TNF and IFNγ in serum, was elevated in *Lck*‐Cre;*Ptpn2* ^*fl*/*fl*^;*p53* ^+/−^ mice (Appendix Fig S2A), as seen in aged *Lck*‐Cre;*Ptpn2* ^*fl*/*fl*^ mice (Appendix Fig S2B), but this did not exceed that occurring in *Ptpn2* ^*fl*/*fl*^;*p53* ^+/−^ littermate controls. Aged *Lck*‐Cre;*Ptpn2* ^*fl*/*fl*^;*p53* ^+/−^ mice also had lymphocytic infiltrates in their livers (Appendix Fig S2C), forming what resembled ectopic lymphoid‐like structures (Pitzalis *et al*, 2014), and this was accompanied by liver damage and ensuing fibrosis (Appendix Fig S2C). However, lymphocytic infiltrates and fibrosis were also evident in the livers of tumour‐bearing *Ptpn2* ^*fl*/*fl*^;*p53* ^+/−^ mice (Appendix Fig S2C). Taken together, these results indicate that PTPN2 deficiency in T cells can prevent the formation of tumours induced by *p53* LOH without exacerbating inflammation.

### PTPN2 deficiency enhances T‐cell‐mediated immunosurveillance

At least one mechanism by which PTPN2 deficiency might prevent tumour formation in *p53* ^+/−^ mice might be through the promotion of T‐cell‐mediated tumour immunosurveillance. To explore this, we first assessed the growth of syngeneic tumours arising from ovalbumin (OVA)‐expressing AT‐3 (AT‐3‐OVA) mammary carcinoma cells implanted into the inguinal mammary fat pads of *Ptpn2* ^*fl*/*fl*^ versus *Lck*‐Cre;*Ptpn2* ^*fl*/*fl*^ C57BL/6 mice (Fig 1C); AT‐3 cells lack oestrogen receptor, progesterone receptor and ErbB2 expression and are a model of triple‐negative breast cancer (Stewart & Abrams, 2007; Mattarollo *et al*, 2011). Whereas AT3‐OVA cells grew readily in *Ptpn2* ^*fl*/*fl*^ mice, tumour growth was markedly repressed in *Lck*‐Cre;*Ptpn2* ^*fl*/*fl*^ mice so that tumour progression was prevented in 5/13 mice and eradicated in 2/8 of the remaining mice after tumours had developed. The repression of tumour growth was accompanied by the infiltration of CD4^+^ and CD8^+^ effector/memory (CD44^hi^CD62L^lo^) T cells into tumours (Fig 1D). Consistent with our previous studies (Wiede *et al*, 2011), PTPN2‐deficient CD25^hi^FoxP3^+^ regulatory T cells (T_regs_) were increased rather than decreased in AT‐3‐OVA tumours (Appendix Fig S2D) and their activation was moderately enhanced (Appendix Fig S2E) precluding the repression of tumour growth being due to defective T_reg_‐mediated immunosuppression. Moreover, tumour‐infiltrating PTPN2‐deficient CD4^+^ and CD8^+^ effector/memory T cells were significantly more active, as assessed by the PMA/ionomycin‐induced production of markers of T‐cell cytotoxicity *ex vivo*, including interferon (IFN)‐γ and tumour necrosis factor (TNF) (Fig 1E). To directly assess the influence of PTPN2 deficiency on T‐cell‐mediated immunosurveillance, we next isolated tumour‐infiltrating CD8^+^ T cells from *Ptpn2* ^*fl*/*fl*^ versus *Lck*‐Cre;*Ptpn2* ^*fl*/*fl*^ mice and assessed their activation by measuring IFNγ production *ex vivo* upon re‐challenge with tumour cells isolated from AT3‐OVA tumours that had developed in *Ptpn2* ^*fl*/*fl*^ mice (Fig 1F). *Ptpn2* ^*fl*/*fl*^ tumour‐infiltrating CD8^+^ T cells remained largely unresponsive when re‐challenged (Fig 1F), consistent with tolerisation. By contrast, PTPN2‐deficient T cells exhibited significant increases in IFNγ consistent with increased effector activity (Fig 1F). These findings point towards PTPN2 having an integral role in T‐cell tolerance and immune surveillance.

**Figure 2:**
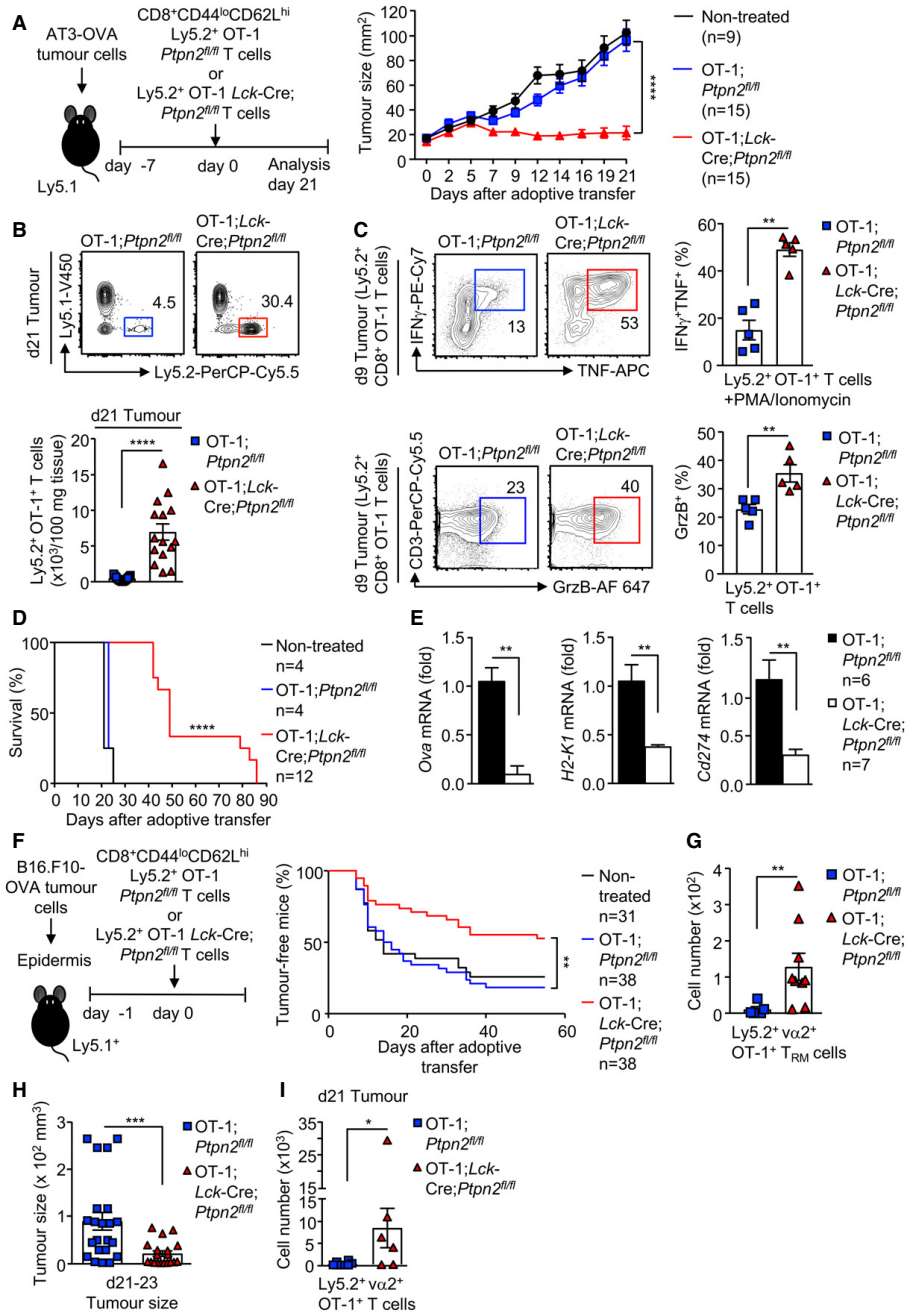
PTPN2 deletion enhances CD8^+^ T‐cell‐mediated immunosurveillance A–DAT‐3‐OVA mammary tumour cells (1 × 10^6^) were injected into the fourth inguinal mammary fat pads of female Ly5.1^+^ mice. Seven days after tumour injection, FACS‐purified 2 × 10^6^ naïve CD8^+^CD44^lo^CD62L^hi^ lymph node T cells from Ly5.2^+^;OT‐1;*Ptpn2* ^*fl*/*fl*^ versus Ly5.2^+^;OT‐1;*Lck*‐Cre;*Ptpn2* ^*fl*/*fl*^ mice were adoptively transferred into tumour‐bearing Ly5.1^+^ mice. Tumour‐bearing Ly5.1^+^ mice were monitored for (A) tumour growth over 21 days and (D) for survival over 86 days. (B) After 21 days, TILs were processed for flow cytometry and donor T‐cell numbers (Ly5.1^−^Ly5.2^+^) determined. (C) After 9 days, the proportion of Ly5.2^+^IFNγ^+^TNF^+^ versus Ly5.2^+^GrzB^+^ TILs was determined.EGene expression in tumours from mice treated with Ly5.2^+^;OT‐1;*Ptpn2* ^*fl*/*fl*^ T cells 21 days post‐adoptive transfer versus those re‐emerging in mice treated with Ly5.2^+^;OT‐1;*Lck*‐Cre;*Ptpn2* ^*fl*/*fl*^ T cells.F–IB16.F10‐OVA melanoma cells (1 × 10^5^) were engrafted onto the abraded skin in the flanks of Ly5.1^+^ mice. 24 h after tumour cell engraftment, naïve CD8^+^CD44^lo^CD62L^hi^ lymph node T cells from Ly5.2^+^;OT‐1;*Ptpn2* ^*fl*/*fl*^ versus Ly5.2^+^;OT‐1;*Lck*‐Cre;*Ptpn2* ^*fl*/*fl*^ mice were adoptively transferred and tumour incidence monitored. (G) Epidermal lymphocytes from tumour‐free mice were stained for CD69^hi^CD103^hi^ and donor‐derived (Ly5.2^+^vα2^+^) tissue‐resident memory T cells (T_RMs_) determined by flow cytometry. (H) Tumour sizes in B16.F10‐OVA melanoma bearing mice were determined between days 21 and 23. (I) TILs were assessed for Ly5.2^+^;OT‐1;*Ptpn2* ^*fl*/*fl*^ and Ly5.2^+^;OT‐1;*Lck*‐Cre;*Ptpn2* ^*fl*/*fl*^ donor T‐cell numbers by flow cytometry.Data information: Representative flow cytometry profiles and results (means ± SEM) from at least two independent experiments are shown. In (A), significance was determined using 2‐way ANOVA test and in (B, C, E, G, H, I) significance determined using 2‐tailed Mann–Whitney *U*‐test. In (D, F), significance was determined using log‐rank (Mantel–Cox) test. *P < 0.05, **P < 0.01, ***P < 0.001, ****P < 0.0001.

To explore the cellular mechanisms by which PTPN2 deficiency might enhance immunosurveillance, we determined whether PTPN2 deletion might promote the tumour‐specific activity of adoptively transferred CD8^+^ T cells expressing the OT‐1 TCR specific for the ovalbumin (OVA) peptide SIINFEKL. Naive OT‐1 T cells can undergo clonal expansion and develop effector function when they engage OVA‐expressing tumours, but thereon leave the tumour microenvironment, become tolerised and fail to control tumour growth (Shrikant & Mescher, 1999; Shrikant *et al*, 1999; Thompson *et al*, 2010). The eradication of solid tumours by naive CD8^+^ T cells is dependent on help from tumour‐specific CD4^+^ T cells (Marzo *et al*, 1999; Shrikant *et al*, 1999). Our previous studies have shown that PTPN2 deficiency enhances TCR‐instigated responses and negates the need for CD4^+^ T‐cell help in the context of antigen cross‐presentation (Wiede *et al*, 2014b). Accordingly, we determined whether PTPN2 deficiency might overcome tolerisation and render naive OT‐1 CD8^+^ T cells capable of suppressing the growth of OVA‐expressing tumours. To this end, naive OT‐1;*Ptpn2* ^*fl*/*fl*^ or OT‐1;*Lck*‐Cre;*Ptpn2* ^*fl*/*fl*^ CD8^+^ T cells were adoptively transferred into immunocompetent and non‐irradiated congenic C57BL/6 hosts bearing syngeneic tumours arising from AT‐3‐OVA cells inoculated into the mammary fat pad (Fig 2A). As expected (Shrikant & Mescher, 1999; Shrikant *et al*, 1999), adoptively transferred naive (CD44^lo^CD62L^hi^) *Ptpn2* ^*fl*/*fl*^ OT‐1 CD8^+^ T cells had no overt effect on the growth of AT‐3‐OVA mammary tumours when compared to vehicle‐treated tumour‐bearing mice (Fig 2A). By contrast 5 days after adoptive transfer, *Lck*‐Cre;*Ptpn2* ^*fl*/*fl*^ OT‐1 T cells completely repressed tumour growth (Fig 2A). The repression of tumour growth was accompanied by an increase in *Lck*‐Cre;*Ptpn2* ^*fl*/*fl*^ OT‐1 T cells in the draining lymph nodes of the tumour‐bearing mammary glands (Appendix Fig S3A) and a marked increase in tumour‐infiltrating *Lck*‐Cre;*Ptpn2* ^*fl*/*fl*^ OT‐1 T cells (Fig 2B; Appendix Fig S3B). At 9 days post‐adoptive transfer both tumour and draining lymph node *Lck*‐Cre;*Ptpn2* ^*fl*/*fl*^ OT‐1 T cells were more active, as assessed by the PMA/ionomycin‐induced expression of effector molecules, including IFNγ, TNF and granzyme B (Fig 2C; Appendix Fig S3C). Although the expression of the T‐cell inhibitory receptors PD‐1 and Lag‐3 on tumour‐infiltrating PTPN2‐deficient OT‐1 T cells at 9 days post‐transfer was not altered (Appendix Fig S3D), by 21 days post‐transfer relative PD‐1 and LAG‐3 levels were reduced and CD44 was increased on PTPN2‐deficient tumour‐infiltrating and draining lymph node OT‐1 T cells when compared to *Ptpn2* ^*fl*/*fl*^ controls (Appendix Fig S3E–G), consistent with decreased T‐cell exhaustion. AT3‐OVA tumours in mice treated with PTPN2‐deficient OT‐1 CD8^+^ T cells started to re‐emerge after 21 days, but survival was prolonged for as long as 86 days (Fig 2D; Appendix Fig S3H); by contrast, control mice achieved the maximum ethically permissible tumour burden (200 mm^2^) by 25 days. Tumour re‐emergence in this setting was accompanied by decreased OVA and MHC class I (*H2‐k1*) gene expression, consistent with decreased antigen presentation; tumour re‐emergence was also accompanied by decreased PD‐L1 (*Cd274*) gene expression (Fig 2E), but this probably followed decreased MHC class I‐mediated antigen presentation and thereby T‐cell recruitment and inflammation. Taken together, these results are consistent with PTPN2 deficiency increasing the functional activity and attenuating the tolerisation of naïve CD8^+^ T cells to suppress tumour growth.

Next, we determined whether PTPN2 deficiency might promote T‐cell‐mediated immunosurveillance and anti‐tumour activity in a different tumour setting (Fig 2F–I). Specifically, we utilised an orthotopic transplant model of cutaneous melanoma, where OVA‐expressing tumour growth occurs within the epidermis and dermis, mimicking the human condition (Wylie *et al*, 2015). This model requires that circulating OT‐1 CD8^+^ T cells traffic to the epidermis where they differentiate into non‐recirculating CD69^+^ CD103^+^ tissue‐resident memory T (T_RM_) cells that contribute to immunosurveillance and the suppression of solid tumour formation (Park *et al*, 2019). Because of the sporadic nature of tumour development and the protrusion of tumours into the dermal layer (Wylie *et al*, 2015), we monitored for the number of mice that were macroscopically tumour‐free at any one time and measured volumes after resection. We found that 55% of mice receiving PTPN2‐deficient OT‐1 naive CD8^+^ T cells were tumour‐free at 55 days post‐adoptive transfer, whereas only 18% of mice receiving control OT‐1 naive CD8^+^ T cells were tumour‐free (Fig 2F) and this was accompanied by the increased presence of OT‐1 CD8^+^ T_RMs_ in the skin (Fig 2G). These findings are consistent with PTPN2 deficiency promoting the differentiation of circulating naive OT‐1 T cells into T_RMs_ to prevent tumour formation_._ Moreover, even where tumours were evident in mice treated with PTPN2‐deficient OT‐1 T cells, tumour volumes were 10‐fold lower (Fig 2H) and this was accompanied by the increased tumour T‐cell infiltration (Fig 2I). Taken together, these results point towards T‐cell‐specific PTPN2 deficiency promoting anti‐tumour activity in a setting where T_RM_ cells are primarily responsible for immunosurveillance.

### PTPN2 deficiency enhances CAR T‐cell cytotoxicity

CAR T cells are autologous T cells engineered to express a transmembrane CAR specific for a defined tumour antigen that signals via canonical TCR signalling intermediates such as LCK (Yong *et al*, 2017; Davenport *et al*, 2018). CAR T cells targeting CD19 have especially been impressive in the treatment of ALL, with clinical response rates of up to 90% in paediatric B‐cell ALL patients (Grupp *et al*, 2013; Maude *et al*, 2014). However, therapeutic efficacies of CAR T cells in other malignancies, including solid tumours, have been relatively poor (Fesnak *et al*, 2016; Yong *et al*, 2017). Given our findings on PTPN2 in T‐cell‐mediated immunosurveillance and anti‐tumour immunity, we determined whether targeting PTPN2 might enhance the function of CAR T cells in solid tumours. In particular, we assessed the therapeutic efficacy of second‐generation CAR T cells harbouring the intracellular signalling domains of CD28 and CD3ζ and targeting the human orthologue of murine ErbB2/Neu, HER‐2 (Haynes *et al*, 2002). HER‐2 is overexpressed in many solid tumours, including 20% of breast cancers, where it promotes tumour aggressiveness and metastasis (Arteaga *et al*, 2011).

**Figure EV1:**
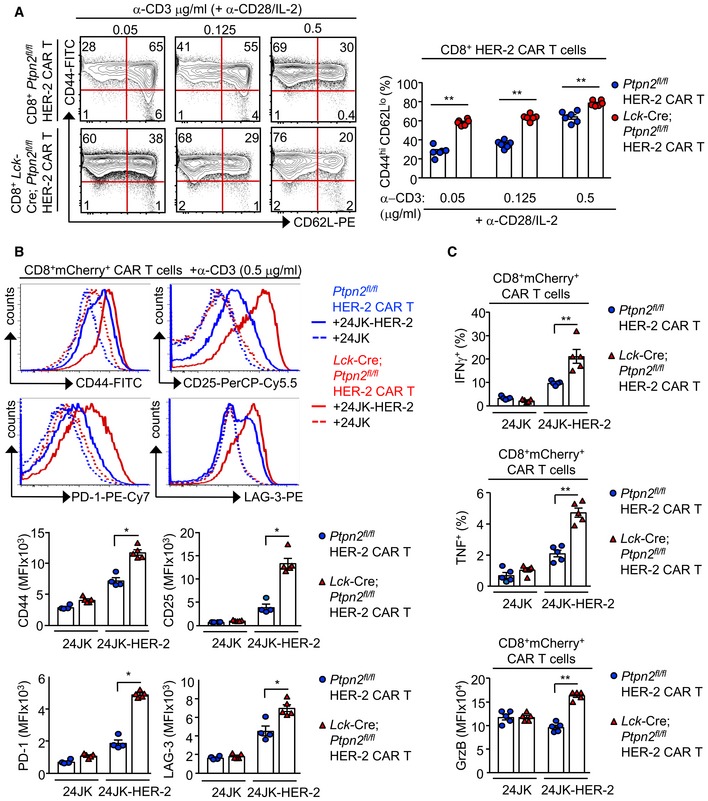
PTPN2 deficiency enhances the generation and the antigen‐induced activation of CAR T cells A
*Ptpn2* ^*fl*/*fl*^ versus *Lck*‐Cre;*Ptpn2* ^*fl*/*fl*^ HER‐2 CAR T cells were generated with varying concentrations of α‐CD3 (0.05, 0.125 and 0.5 μg/ml) in the presence of α‐CD28 (0.5 μg/ml) and IL‐2 (2 ng/ml). After 6 days in culture, the generation of effector/memory (CD44^hi^CD62L^lo^) CD8^+^ HER‐2 CAR T cells was determined by flow cytometry.B, C
*Ptpn2* ^*fl*/*fl*^ versus *Lck*‐Cre;*Ptpn2* ^*fl*/*fl*^ HER‐2 CAR T cells were incubated with HER‐2‐expressing 24JK sarcoma cells (24JK‐HER‐2) and HER‐2‐negative 24JK sarcoma cells. (B) CD44, CD25, PD‐1 and LAG‐3 MFIs, (C) the proportion of CD8^+^IFNγ^+^ versus CD8^+^TNF^+^ CAR T cells and GrzB mean fluorescence intensity (MFI) were determined by flow cytometry.Data information: Significance in (A–C) was determined using 2‐tailed Mann–Whitney *U*‐test. *P < 0.05, **P < 0.01.

First, we assessed the impact of PTPN2 deletion on CAR T cells *in vitro*. Consistent with PTPN2's role in setting thresholds for TCR‐instigated responses (Wiede *et al*, 2011, 2014a,b), we found that PTPN2 deficiency resulted in tenfold lower concentrations of TCR crosslinking antibodies (α‐CD3ε) being required for the maximal generation of CD8^+^ HER‐2 CAR T cells *in vitro*, with the resulting CAR T cells being predominated by the effector/memory (CD44^hi^CD62L^lo^) subset (Fig EV1A). Next, we assessed the impact of PTPN2 deficiency on antigen‐induced CAR T‐cell activation *in vitro*. PTPN2‐deficient CD8^+^ HER‐2 CAR T cells were more activated, as assessed by the expression of CD44, CD25, PD‐1 and LAG‐3, after overnight incubation with HER‐2‐expressing 24JK (24JK‐HER‐2) sarcoma cells, but importantly, not HER‐2‐negative 24JK control cells (Fig EV1B). Moreover, PTPN2‐deficient CD8^+^ HER‐2 CAR T cells exhibited increased antigen‐specific cytotoxic capacity *in vitro* (Fig EV1C; Appendix Fig S4A), as assessed by the increased intracellular expression of IFNγ (required for tumour eradication by CAR T cells *in vivo*; Moeller *et al*, 2005), TNF and granzyme B upon challenge with 24JK‐HER‐2 cells but not 24JK cells. Moreover, both effector/memory (CD44^hi^CD62L^lo^) and central memory (CD44^hi^CD62L^hi^) PTPN2‐deficient CD8^+^ HER‐2 CAR T cells were more effective at specifically killing 24JK‐HER‐2 cells but not 24JK cells *in vitro* (Appendix Fig S4B). Taken together, these results are consistent with PTPN2 deficiency enhancing not only the generation, but also the antigen‐specific activation and cytotoxicity of CAR T cells *in vitro*.

### PTPN2 deficiency enhances LCK‐dependent CAR T‐cell function

**Figure EV2:**
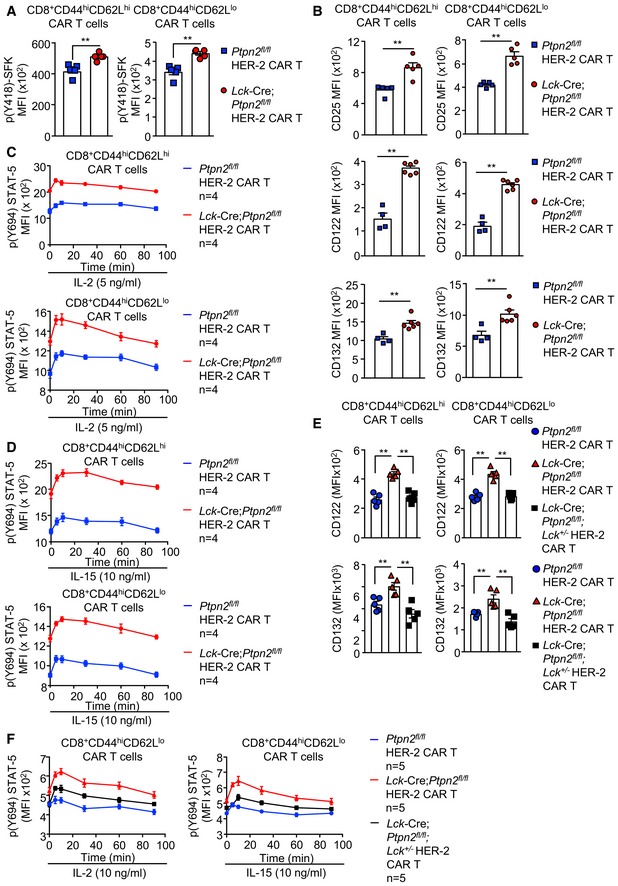
PTPN2 deficiency increases SFK and IL‐2/15‐induced STAT5 signalling in CD8^+^ HER‐2 CAR T cells A
*Ptpn2* ^*fl*/*fl*^ versus *Lck*‐Cre;*Ptpn2* ^*fl*/*fl*^ HER‐2 CD8^+^CD44^hi^CD62L^hi^ or CD44^hi^CD62L^lo^ CAR T cells were assessed for intracellular p(Y418)‐SFK MFI by flow cytometry.BHER‐2‐specific *Ptpn2* ^*fl*/*fl*^ versus *Lck*‐Cre;*Ptpn2* ^*fl*/*fl*^ CAR T cells were incubated with plate‐bound α‐CD3 and CD25; CD122 and CD132 MFIs on CD8^+^CD44^hi^CD62L^hi^ CAR T cells versus CD8^+^CD44^hi^CD62L^lo^ CAR T cells were determined by flow cytometry.C, D
*Ptpn2* ^*fl*/*fl*^ versus *Lck*‐Cre;*Ptpn2* ^*fl*/*fl*^ HER‐2 CAR T cells were incubated with plate‐bound α‐CD3 and stimulated with recombinant (C) IL‐2 and (D) IL‐15 for the indicated time points. Intracellular p(Y694)‐STAT‐5 MFIs on CD8^+^CD44^hi^CD62L^hi^ versus CD8^+^CD44^hi^CD62L^lo^ CAR T cells were determined by flow cytometry.E
*Ptpn2* ^*fl*/*fl*^, *Lck*‐Cre;*Ptpn2* ^*fl*/*fl*^ and *Lck*‐Cre;*Ptpn2* ^*fl*/*fl*^;*Lck* ^+/−^ HER‐2 CAR T cells were incubated with plate‐bound α‐CD3; CD122 and CD132 MFIs on CD8^+^CD44^hi^CD62L^lo^ versus CD8^+^CD44^hi^CD62L^hi^ CAR T cells were determined by flow cytometry.F
*Ptpn2* ^*fl*/*fl*^ versus *Lck*‐Cre;*Ptpn2* ^*fl*/*fl*^ versus *Lck*‐Cre;*Ptpn2* ^*fl*/*fl*^;*Lck* ^+/−^ HER‐2 CAR T cells were incubated with plate‐bound α‐CD3 and stimulated with recombinant IL‐2 and IL‐15 for the indicated time points. Intracellular p(Y694)‐STAT‐5 MFIs on CD8^+^CD44^hi^CD62L^lo^ CAR T cells were determined by flow cytometry.Data information: Representative results (means ± SEM) are shown from two independent experiments. In (A, B, E), significance was determined using 2‐tailed Mann–Whitney *U*‐test. **P < 0.01.

**Figure 3:**
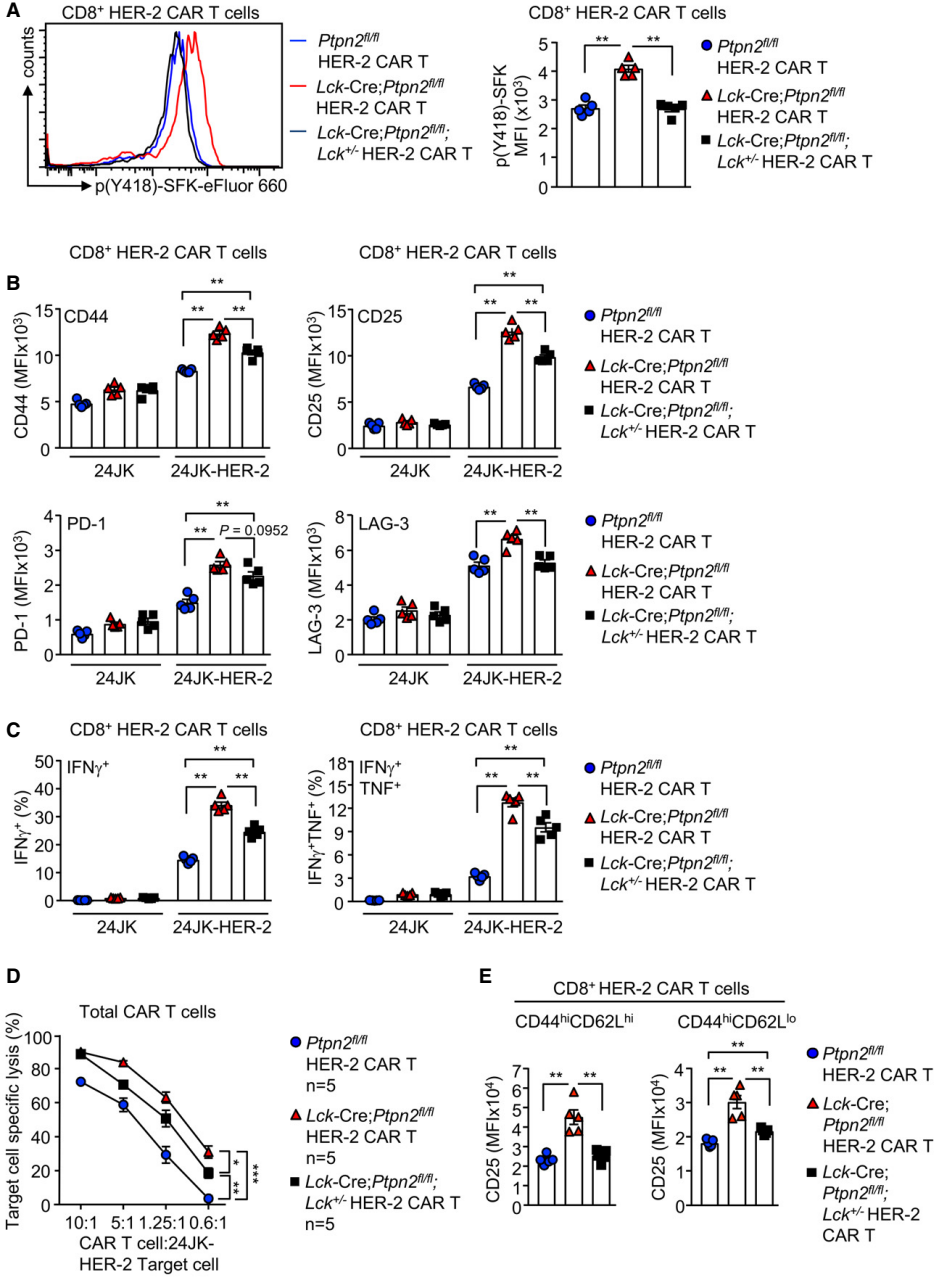
PTPN2 deletion enhances the LCK‐dependent activation of CAR T cells ACD8^+^ HER‐2 CAR T cells generated from *Ptpn2* ^*fl*/*fl*^ versus *Lck*‐Cre;*Ptpn2* ^*fl*/*fl*^ versus *Lck*‐Cre;*Ptpn2* ^*fl*/*fl*^;*Lck* ^+/−^ splenocytes were stained for intracellular p(Y418)‐SFK, and p(Y418)‐SFK MFIs were determined by flow cytometry.BHER‐2‐specific *Ptpn2* ^*fl*/*fl*^ versus *Lck*‐Cre;*Ptpn2* ^*fl*/*fl*^ versus *Lck*‐Cre;*Ptpn2* ^*fl*/*fl*^;*Lck* ^+/−^ CAR T cells were incubated with HER‐2‐expressing 24JK sarcoma cells (24JK‐HER‐2) or HER‐2‐negative 24JK sarcoma cells, and CD44, CD25, PD‐1 and LAG‐3 MFIs on CD8^+^ CAR T cells were determined by flow cytometry.C
*Ptpn2* ^*fl*/*fl*^, *Lck*‐Cre;*Ptpn2* ^*fl*/*fl*^ or *Lck*‐Cre;*Ptpn2* ^*fl*/*fl*^;*Lck* ^+/−^ HER‐2 CAR T cells were incubated with 24JK‐HER‐2 or 24JK sarcoma cells and the proportion of CD8^+^IFNγ^+^ versus CD8^+^IFNγ^+^TNF^+^ CAR T cells determined by flow cytometry.D
*Ptpn2* ^*fl*/*fl*^, *Lck*‐Cre;*Ptpn2* ^*fl*/*fl*^ or *Lck*‐Cre;*Ptpn2* ^*fl*/*fl*^;*Lck* ^+/−^ HER‐2 CAR T cells were incubated with 5 μM CTV‐labelled (CTV^bright^) 24JK‐HER‐2 and 0.5 μM CTV‐labelled (CTV^dim^) 24JK sarcoma cells. Antigen‐specific target cell lysis was monitored for the depletion of CTV^bright^ 24JK‐HER‐2 cells by flow cytometry.EHER‐2‐specific *Ptpn2* ^*fl*/*fl*^, *Lck*‐Cre;*Ptpn2* ^*fl*/*fl*^ and *Lck*‐Cre;*Ptpn2* ^*fl*/*fl*^;*Lck* ^+/−^ CAR T cells were incubated with plate‐bound α‐CD3 and CD25 MFIs on CD8^+^CD44^hi^CD62L^lo^ versus CD8^+^CD44^hi^CD62L^hi^ CAR T cells determined by flow cytometry.Data information: Representative histograms and results (means ± SEM) from at least two independent experiments are shown. In (A–C, E) significance determined using 2‐tailed Mann–Whitney *U*‐test. In (D), significance was determined using 2‐way ANOVA test. *P < 0.05, **P < 0.01, ***P < 0.001.

**Figure EV3:**
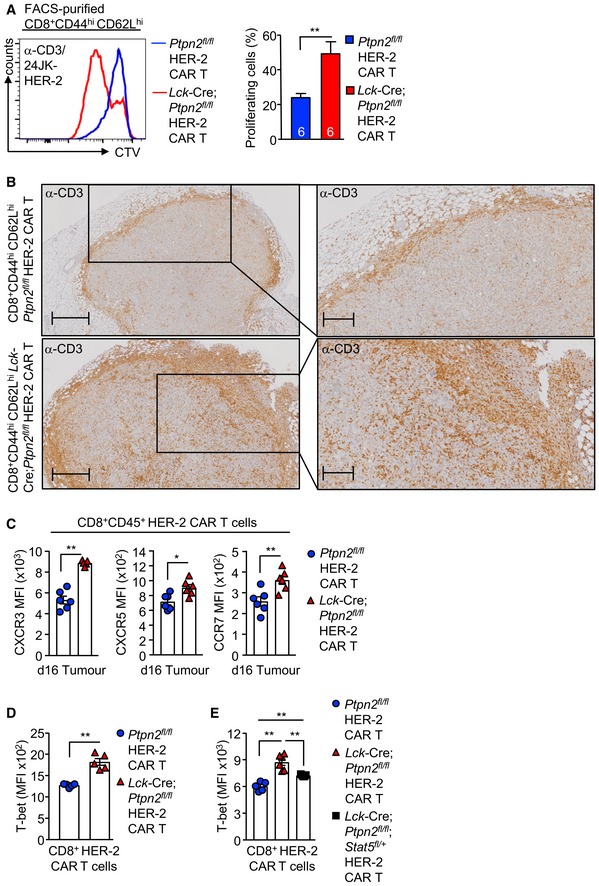
PTPN2 deficiency enhances CAR T‐cell proliferation and tumour infiltration ACD8^+^CD44^hi^CD62L^hi^ HER‐2 CAR T cells generated from *Ptpn2* ^*fl*/*fl*^ versus *Lck*‐Cre;*Ptpn2* ^*fl*/*fl*^ splenocytes were stimulated with plate‐bound α‐CD3 and subsequently labelled with CTV and incubated with 24JK‐HER‐2 cells, and proliferation was determined by flow cytometry.BHER‐2‐E0771 tumours isolated from HER‐2 TG mice on day 10 after adoptive *Ptpn2* ^*fl*/*fl*^ versus *Lck*‐Cre;*Ptpn2* ^*fl*/*fl*^ HER‐2 CAR T‐cell transfer were analysed for CD3^+^ T‐cell infiltrates by immunohistochemistry. Scale bars: 500 μm (full size) and 200 μm (zoom).CHER‐2‐E0771 cells (2 × 10^5^) were injected into the fourth inguinal mammary fat pads of female HER‐2 transgenic (TG) mice. Six days after tumour injection, HER‐2 TG mice received total body irradiation (4 Gy) followed by the adoptive transfer of 6 × 10^6^ FACS‐purified CD8^+^CD62L^hi^CD44^hi^ central memory HER‐2 CAR T cells generated from *Ptpn2* ^*fl*/*fl*^ versus *Lck*‐Cre;*Ptpn2* ^*fl*/*fl*^ splenocytes. Mice were injected with IL‐2 (50,000 IU/day) on days 0–4 after adoptive CAR T‐cell transfer. Lymphocytes were isolated from the tumours at day 16 post‐adoptive transfer and CXCR3, CXCR5 and CCR7 MFIs on CD45^+^CD8^+^ T cells determined by flow cytometry.D, EIntracellular T‐bet MFIs in CD8^+^ HER‐2 CAR T cells were determined by flow cytometry.Data information: Representative flow cytometry profiles and results (means ± SEM) are shown from two independent experiments. In (A, C, D, E), significance was determined using 2‐tailed Mann–Whitney *U*‐test. *P < 0.05, **P < 0.01.

Next, we explored the mechanisms by which PTPN2 deficiency may influence CAR T‐cell activation and function. PTPN2 dephosphorylates and inactivates the SFK LCK to tune TCR signalling so that T cells can differentially respond to self versus non‐self (Wiede *et al*, 2011, 2014a,b, 2017a). CAR T cells are reliant on canonical TCR signalling intermediates, including LCK for their activation and function (Davenport *et al*, 2018). Accordingly, we assessed the influence of PTPN2 deficiency on the activation of LCK in CAR T cells by monitoring for the phosphorylation of Y394 (using antibodies specific for Y418‐phosphorylated SFKs). PTPN2 deficiency significantly increased SFK Y418 phosphorylation in CD8^+^ HER‐2 CAR T cells (Figs EV2A and 3A). We have shown previously that the enhanced TCR‐induced T‐cell activation resulting from PTPN2 deficiency is accompanied by the increased expression of the interleukin (IL)‐2 receptor chains CD25 (IL‐2 receptor α chain) and CD122 (IL‐2/15 receptor β chain) and IL‐2 induced STAT‐5 signalling (Wiede *et al*, 2011, 2014b). Consistent with this, we found that CD25, CD122 and CD132 (common γ chain shared by IL‐2 and IL‐15) receptor levels were elevated in activated PTPN2‐deficient CAR T cells (Fig EV2B) and this was accompanied by increased basal and IL‐2/15‐induced STAT‐5 Y694 phosphorylation (Fig EV2C and D). To explore the extent to which the increased SFK signalling may contribute to this and the enhanced CAR T‐cell activation and function, we crossed *Lck*‐Cre;*Ptpn2* ^*fl*/*fl*^ mice onto the *Lck* ^+/−^ background (Wiede *et al*, 2017a) so that total LCK would be reduced by 50% and LCK signalling may more closely approximate that in *Ptpn2* ^*fl*/*fl*^ controls. Consistent with this, we found that SFK Y418 phosphorylation in *Lck*‐Cre;*Ptpn2* ^*fl*/*fl*^;*Lck* ^+/−^ CD8^+^ HER‐2 CAR T cells was reduced to that in *Ptpn2* ^*fl*/*fl*^ controls (Fig 3A). Strikingly, *Lck* heterozygosity attenuated the enhanced antigen‐specific HER‐2 CAR T‐cell activation (as monitored by CD44, CD25, PD‐1 and LAG‐3 levels; Fig 3B) and cytotoxic potential (as measured by the antigen‐induced expression of IFNγ and TNF; Fig 3C) and the enhanced capacity of PTPN2‐deficient CAR T cells to specifically kill HER‐2‐expressing tumour cells (Fig 3D). In addition, *Lck* heterozygosity corrected the enhanced IL‐2/15 receptor levels (Figs 3E and EV2E) and partially corrected the enhanced IL‐2/15‐induced STAT‐5 signalling (Fig EV2F). The persistent increased STAT‐5 signalling despite correcting IL‐2/15 receptor levels is consistent with previous studies showing that STAT‐5 can also serve as direct a *bona fide* substrate of PTPN2 and that PTPN2 deficiency promotes cytokine‐induced STAT‐5 signalling in thymocytes/T cells (Simoncic *et al*, 2002; Tiganis & Bennett, 2007; Wiede *et al*, 2011, 2014a, 2017a; Gurzov *et al*, 2014). Irrespective, these results are consistent with PTPN2 deficiency enhancing the antigen‐specific activation and function of CAR T cells through the promotion of LCK signalling.

### PTPN2‐deficient CAR T cells eradicate solid tumours

**Figure 4:**
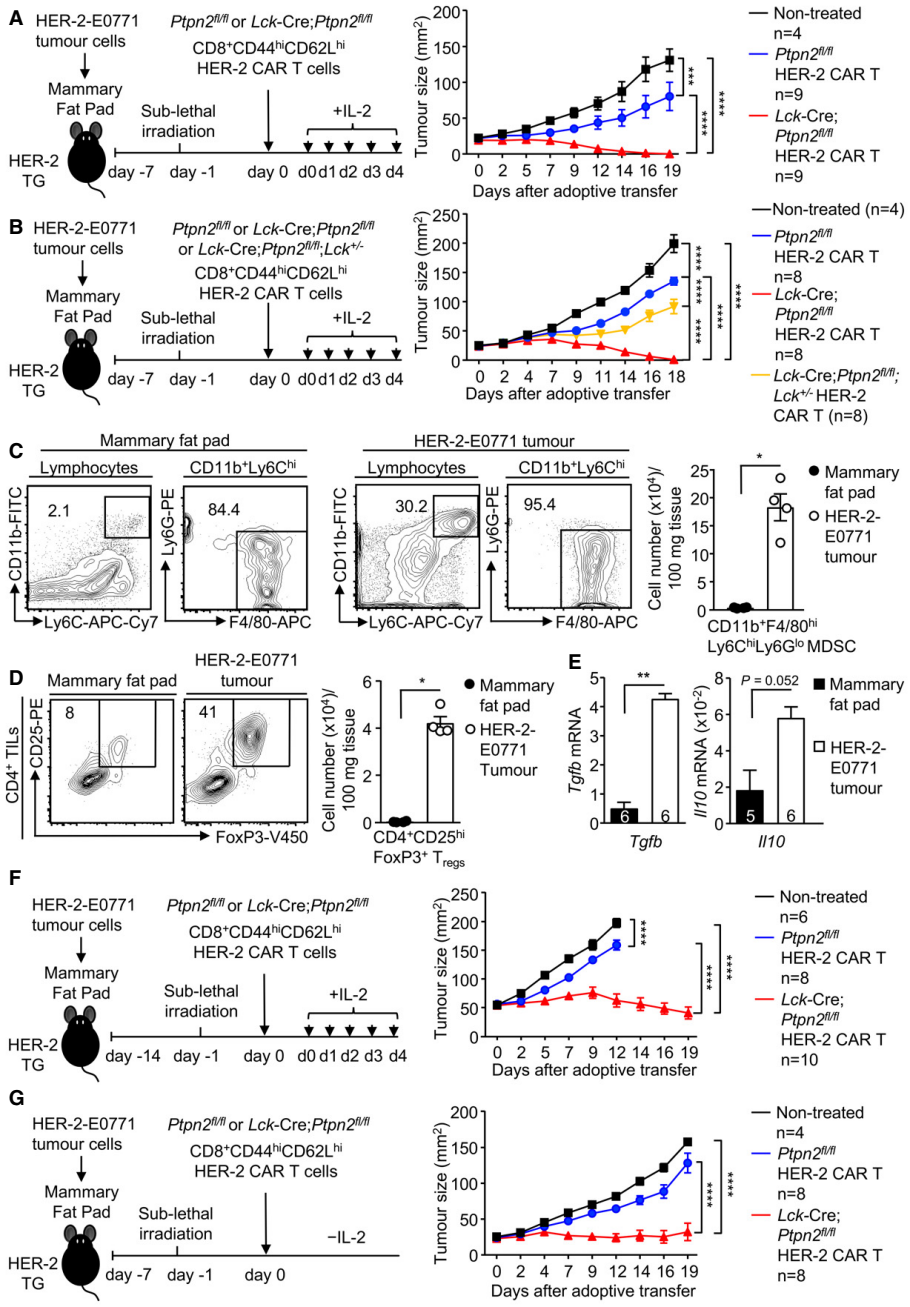
PTPN2 deletion enhances CAR T‐cell efficacy *in vivo* A, BHER‐2‐E0771 mammary tumour cells (2 × 10^5^) were injected into the fourth inguinal mammary fat pads of female HER‐2 TG mice. Six days after tumour injection, HER‐2 TG mice received total body irradiation (4 Gy) followed by the adoptive transfer of 6 × 10^6^ FACS‐purified CD8^+^CD44^hi^CD62L^hi^ central memory HER‐2 CAR T cells generated from *Ptpn2* ^*fl*/*fl*^ versus *Lck*‐Cre;*Ptpn2* ^*fl*/*fl*^ or *Ptpn2* ^*fl*/*fl*^ versus *Lck*‐Cre;*Ptpn2* ^*fl*/*fl*^ versus *Lck*‐Cre;*Ptpn2* ^*fl*/*fl*^;*Lck* ^+/−^ splenocytes. Mice were injected with IL‐2 (50,000 IU/day) on days 0–4 after adoptive CAR T‐cell transfer and monitored for tumour growth.C, DTILs isolated from HER‐2‐E0771 mammary tumour versus lymphocytes isolated from the contralateral mammary fat pads were analysed for (C) CD11b^+^ F4/80^hi^Ly6C^hi^Ly6G^lo^ myeloid‐derived suppressor cells (MDSC) and (D) CD4^+^CD25^hi^FoxP3^+^ regulatory T cells (T_regs_) by flow cytometry.E
*Tgfb* and *Il10* mRNA levels in HER‐2‐E0771 tumours and mammary fat pads were assessed by quantitative real‐time PCR.F, GHER‐2‐E0771 mammary tumour cells (2 × 10^5^) were injected into the fourth inguinal mammary fat pads of female HER‐2 TG mice. (F) 13 or (G) 6 days after tumour injection, HER‐2 TG mice received total body irradiation (4 Gy) followed by the adoptive transfer of 6 × 10^6^ FACS‐purified CD8^+^CD44^hi^CD62L^hi^ central memory HER‐2 CAR T cells generated from *Ptpn2* ^*fl*/*fl*^ versus *Lck*‐Cre;*Ptpn2* ^*fl*/*fl*^ splenocytes. Mice were injected with (F) IL‐2 (50,000 IU/day) or (G) saline on days 0–4 after adoptive CAR T‐cell transfer and monitored for tumour growth.Data information: Representative flow cytometry profiles and results (means ± SEM) from three independent experiments are shown. In (A, B, F, G), significance was determined using 2‐way ANOVA test. In (C–E), significance was determined using 2‐tailed Mann–Whitney *U*‐test. *P < 0.05, **P < 0.01, ***P < 0.001, ****P < 0.0001.

To explore the therapeutic efficacy of PTPN2‐deficient HER‐2‐targeting CAR T cells *in vivo,* we adoptively transferred a single dose (6 × 10^6^) of purified *Ptpn2* ^*fl*/*fl*^ versus *Lck*‐Cre;*Ptpn2* ^*fl*/*fl*^ central memory CD8^+^ HER‐2 CAR T cells into sub‐lethally irradiated syngeneic recipients bearing established orthotopic tumours arising from the injection of HER‐2‐expressing E0771 (HER‐2‐E0771) mammary tumour cells (Fig 4). We adoptively transferred central memory CAR T cells as these cells engraft better and elicit persistent anti‐tumour responses (Klebanoff *et al*, 2012). In addition, as lymphodepletion prior to T‐cell infusion is used routinely in the clinic to facilitate T‐cell expansion and enhance efficacy (Brentjens *et al*, 2011), we immunodepleted mice by sublethal irradiation (400 cGy) as is routine in murine CAR T‐cell studies (Beavis *et al*, 2017). Notably, HER‐2‐E0771 cells were grafted into HER‐2 transgenic (TG) mice, where HER‐2 expression was driven by the whey acidic protein (WAP) promoter that induces expression in the cerebellum and the lactating mammary gland (Piechocki *et al*, 2003), so that HER‐2‐expressing orthotopic tumours would be regarded as self and host anti‐tumour immunity repressed. Previous studies have shown that effective tumour killing and eradication by CD8^+^ CAR T cells are reliant on the presence of CD4^+^ CAR T cells (Moeller *et al*, 2005). Strikingly, we found that although PTPN2‐expressing central memory CD8^+^ HER‐2 CAR T cells modestly suppressed HER‐2‐E0771 mammary tumour growth, PTPN2‐deficient CD8^+^ HER‐2 CAR T cells eradicated tumours (Fig 4A). The ability of PTPN2‐deficient CAR T cells to suppress tumour growth and eradicate tumours was reliant on the enhanced activation of LCK, as this was significantly, albeit not completely abrogated by *Lck* heterozygosity (Fig 4B) that corrected the enhanced LCK activation in *Lck*‐Cre;*Ptpn2* ^*fl*/*fl*^ CAR T cells *in vitro* (Fig 3A). The repression of tumour growth by PTPN2‐deficient CD8^+^ HER‐2 CAR T cells occurred despite tumours harbouring an immunosuppressive microenvironment (Topalian *et al*, 2015; Popovic *et al*, 2018), with increased immunosuppressive myeloid‐derived suppressor cells (MDSCs) (Fig 4C) and T_regs_ (Fig 4D) and the increased expression of immunosuppressive cytokines, including transforming growth factor β (*Tgfb*) and IL‐10 (*Il10*) (Fig 4E), when compared to normal mammary tissue. PTPN2‐deficient CAR T cells markedly suppressed tumour growth, even when the tumours were allowed to grow to one quarter (50 mm^2^) of the maximal ethically permissible mammary tumour burden prior to CAR T‐cell therapy (Fig 4F). Moreover, PTPN2‐deficient CAR T cells were effective in repressing tumour growth even without the co‐administration of IL‐2 (Fig 4G) that is used routinely in rodent pre‐clinical models to promote CAR T‐cell expansion but is not used in the clinic. Taken together, these results demonstrate that PTPN2 deficiency promotes the LCK‐dependent activation of CAR T cells and overcomes the immunosuppressive tumour microenvironment to eradicate solid tumours *in vivo*.

**Figure 5:**
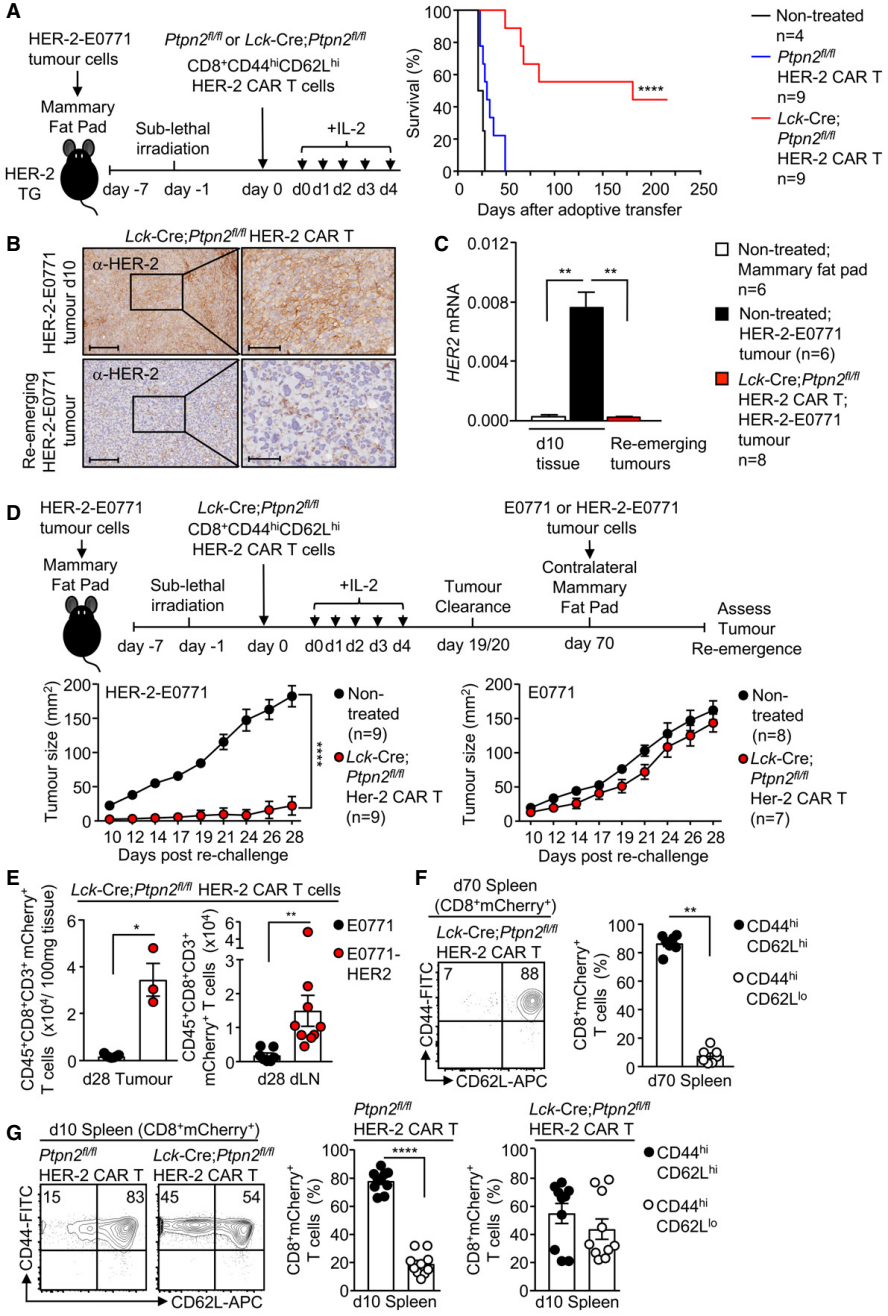
PTPN2 deficiency prevents the re‐emergence of tumours AHER‐2‐E0771 mammary tumour cells (2 × 10^5^) were injected into the fourth inguinal mammary fat pads of female HER‐2 TG mice. Six days after tumour injection, HER‐2 TG mice received total body irradiation (4 Gy) followed by the adoptive transfer of 6 × 10^6^ FACS‐purified *Lck*‐Cre;*Ptpn2* ^*fl*/*fl*^ CD8^+^CD44^hi^CD62L^hi^ central memory HER‐2 CAR T cells. Mice were injected with IL‐2 (50,000 IU/day) on days 0–4 after adoptive CAR T‐cell transfer and monitored for survival.BHER‐2‐E0771 tumours at day 10 post‐adoptive CAR T‐cell transfer or HER‐2‐E0771 tumours that had re‐emerged after being cleared by *Lck*‐Cre;*Ptpn2* ^*fl*/*fl*^ CD8^+^ HER‐2 CAR T cells were analysed for HER‐2 expression by immunohistochemistry. Scale bars: 200 μm (full size) and 70 μm (zoom).CNormal mammary tissue, HER‐2‐E0771 tumours that were not treated with CAR T cells, or those that had re‐emerged after being cleared by *Lck*‐Cre;*Ptpn2* ^*fl*/*fl*^ CD8^+^ HER‐2 CAR T cells were analysed for HER‐2 expression by quantitative PCR.D, EHER‐2‐E0771 versus HER‐2‐negative E0771 mammary tumour cells (2 × 10^5^) were injected into the contralateral fourth inguinal mammary fat pads of female HER‐2 TG mice 70 days after adoptive CAR T‐cell transfer, and mice were monitored for tumour growth. (E) At day 28, the number of CD45^+^CD3^+^CD8^+^ mCherry^+^ CAR T cells in HER‐2‐E0771 versus E0771 tumours and dLNs were determined by flow cytometry.F, G
*Ptpn2* ^*fl*/*fl*^ versus *Lck*‐Cre;*Ptpn2* ^*fl*/*fl*^ HER‐2 CAR T cells isolated from the spleens of HER‐2 TG mice at (F) 70 days and (G) 10 days post‐adoptive transfer were stained for CD8, CD44 and CD62L and analysed by flow cytometry.Data information: Representative flow cytometry profiles and results (means ± SEM) from three independent experiments are shown. In (A), significance was determined using log‐rank (Mantel–Cox) test. In (D) significance was determined using 2‐way ANOVA test. In (C, E–G), significance was determined using 2‐tailed Mann–Whitney *U*‐test. *P < 0.05, **P < 0.01, ****P < 0.0001.

Strikingly, the repression of tumour growth by PTPN2‐deficient HER‐2 CAR T cells persisted long after HER‐2 tumours had been eradicated, so that approximately half of all mice were alive for longer than 200 days with the remaining half succumbing to tumours (Fig 5A). Moreover, the tumours that did re‐emerge lacked HER‐2 as assessed by immunohistochemistry (Fig 5B) and quantitative real‐time PCR (Fig 5C). These results are consistent with PTPN2‐deficient HER‐2 CAR T cells completely eliminating HER‐2‐expressing tumours and eliciting a selective pressure so that any re‐emerging tumours downregulate HER‐2. To explore this further and the influence of PTPN2 deficiency on CAR T‐cell memory, we re‐implanted HER‐2^+^ tumour cells into control HER‐2 transgenic mice, or those mice in which HER‐2^+^ tumours had been previously cleared by PTPN2‐deficient HER‐2 CAR T cells (Fig 5D and E). In those mice in which tumours had previously been cleared, splenic PTPN2‐deficient CAR T cells had a central memory phenotype (CD44^hi^CD62L^hi^) rather than the mixed central and effector/memory (CD44^hi^CD62L^lo^) phenotypes otherwise present on day 10 post‐adoptive transfer (Fig 5F and G), consistent with PTPN2 deficiency promoting CAR T‐cell memory. To assess systemic anti‐tumour immunity, HER‐2^+^ tumours cells were re‐implanted into the previously tumour‐free contralateral mammary fat pads. As a control, we also monitored the growth of HER‐2‐negative E0771 tumour cells. We found that the growth of HER‐2^+^ tumours in the previously tumour‐free contralateral mammary fat pads was markedly repressed or completely prevented (Fig 5D). The repression of tumour growth was accompanied by the increased infiltration of HER‐2 CAR T cells (Fig 5E). By contrast, the growth of HER‐2‐negative mammary E0771 tumours was not affected. These results are consistent with PTPN2 deficiency in HER‐2 CAR T cells promoting CAR T‐cell memory and recall to prevent the re‐emergence of HER‐2^+^ tumours, including those that may arise at distant metastatic sites.

### PTPN2 deficiency promotes CAR T‐cell homing

**Figure 6:**
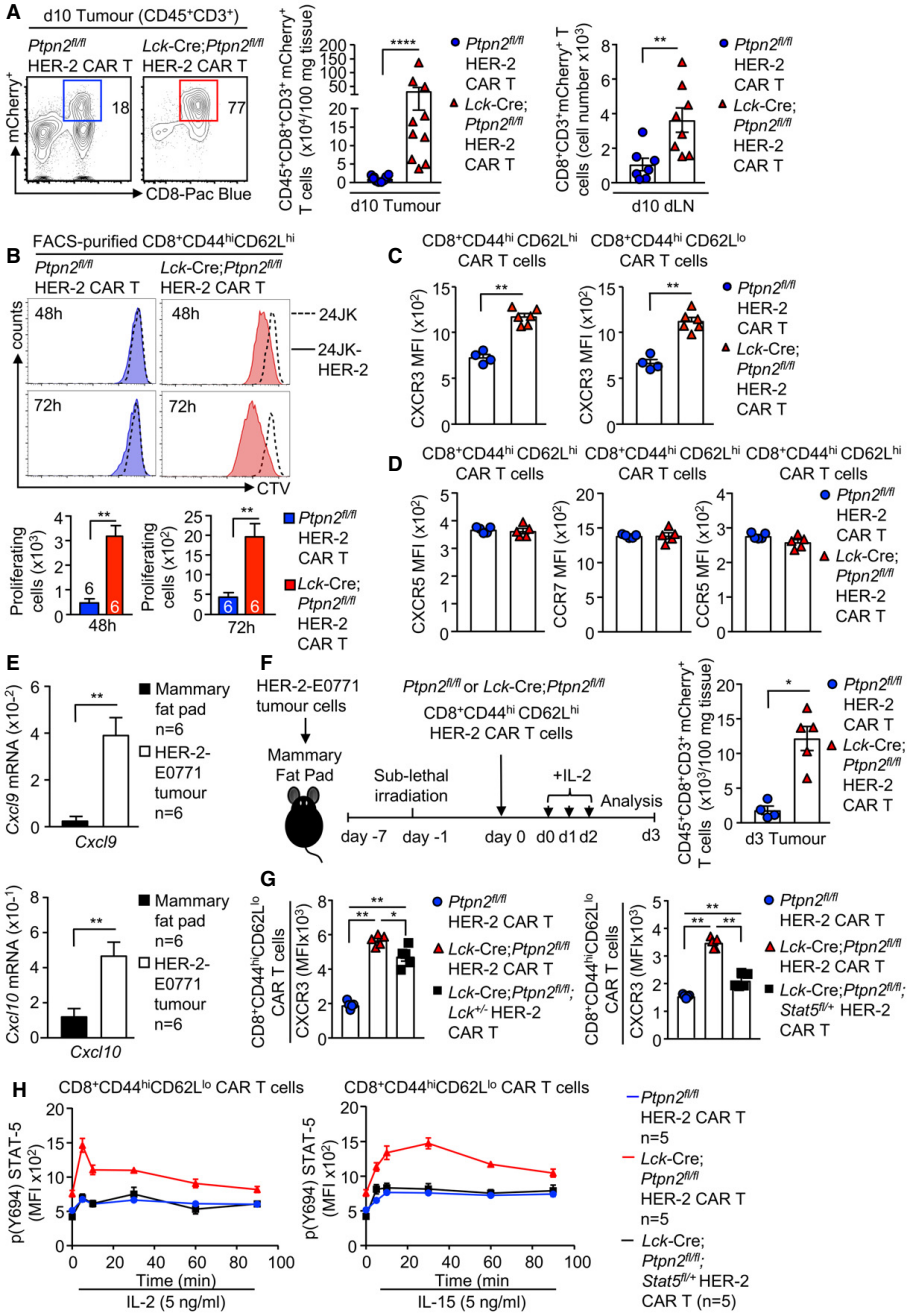
PTPN2 deficiency enhances CXCR3 expression and promotes CAR T‐cell homing HER‐2‐E0771 mammary tumour cells (2 × 10^5^) were injected into the fourth inguinal mammary fat pads of female HER‐2 TG mice. Six days after tumour injection, HER‐2 TG mice received total body irradiation (4 Gy) followed by the adoptive transfer of 6 × 10^6^ FACS‐purified CD8^+^CD62L^hi^CD44^hi^ central memory HER‐2 CAR T cells generated from *Ptpn2* ^*fl*/*fl*^ versus *Lck*‐Cre;*Ptpn2* ^*fl*/*fl*^ splenocytes. Mice were injected with IL‐2 (50,000 IU/day) on days 0–4 after adoptive CAR T‐cell transfer.
ALymphocytes were isolated from the tumours and dLN at day 10 post‐adoptive transfer, and mCherry^+^CD45^+^CD3^+^CD8^+^ CAR T‐cell numbers were determined by flow cytometry.BCTV‐labelled HER‐2 CAR T cells were incubated with 24JK‐HER‐2 or 24JK sarcoma cells and CTV dilution assessed by flow cytometry to monitor proliferation.C, DCXCR3 MFIs on CD8^+^CD44^hi^CD62L^hi^ versus CD8^+^CD44^hi^CD62L^lo^ CAR T cells (C) and CXCR5, CCR7, CCR5 MFIs on CD8^+^CD44^hi^CD62L^hi^ CAR T cells (D) were determined by flow cytometry.E
*Cxcl9* and *Cxcl10* mRNA levels in HER‐2‐E0771 tumours were assessed by quantitative real‐time PCR.FLymphocytes were isolated from HER‐2‐E0771 tumours at day 3 post‐adoptive CAR T‐cell transfer, and mCherry^+^CD45^+^CD3^+^CD8^+^ CAR T‐cell numbers were determined by flow cytometry.G
*Ptpn2* ^*fl*/*fl*^ versus *Lck*‐Cre;*Ptpn2* ^*fl*/*fl*^ versus *Lck*‐Cre;*Ptpn2* ^*fl*/*fl*^;*Lck* ^+/−^ or *Lck*‐Cre;*Ptpn2* ^*fl*/*fl*^;*Stat5* ^*fl*/+^ HER‐2 CAR T cells were incubated with plate‐bound α‐CD3 and CXCR3 MFIs on CD8^+^CD44^hi^CD62L^lo^ CAR T cells determined by flow cytometry.H
*Ptpn2* ^*fl*/*fl*^ versus *Lck*‐Cre;*Ptpn2* ^*fl*/*fl*^ versus *Lck*‐Cre;*Ptpn2* ^*fl*/*fl*^;*Stat5* ^*fl*/+^ HER‐2 CAR T cells were incubated with plate‐bound α‐CD3 and stimulated with recombinant IL‐2 and IL‐15 for the indicated time points. Intracellular p(Y694)‐STAT‐5 MFIs in CD8^+^CD44^hi^CD62L^lo^ were determined by flow cytometry.Data information: Representative flow cytometry profiles and results (means ± SEM) from two independent experiments are shown. In (A, B, C, E, F, G), significance was determined using 2‐tailed Mann–Whitney *U*‐test. *P < 0.05, **P < 0.01, ****P < 0.0001.

The clinical benefit of adoptive T‐cell therapy is highly reliant on the ability of T cells including CAR T cells to home efficiently into the target tissue (Slaney *et al*, 2014; Nagarsheth *et al*, 2017). We found that the repression of tumour growth by PTPN2‐deficient CD8^+^ HER‐2 CAR T cells was accompanied by a marked increase in CD45^+^ CD8^+^ CD3^+^ mCherry^+^ CAR T‐cell abundance in tumours and the corresponding draining lymph nodes (Fig 6A). In part, the increased CAR T‐cell abundance may reflect the expansion of CAR T cells after they engage tumour antigen, as PTPN2 deficiency increased the antigen‐specific proliferation of HER‐2 CAR T cells *in vitro* (Figs 6B and EV3A). However, the increased CAR T‐cell abundance may also reflect an increase in CAR T‐cell homing and infiltration as CD3ε^+^ lymphocytes accumulated within the tumour and at the tumour/stromal interface at 10 days post‐adoptive transfer (Fig EV3B). Previous studies have correlated the accumulation of TILs in tumours with the expression of the chemokine receptor CXCR3 (receptor for CXCL9, CXCL10 and CXCL11) (Slaney *et al*, 2014; Nagarsheth *et al*, 2017). We found that PTPN2‐deficient HER‐2 CAR T cells expressed higher cell surface levels of CXCR3 *in vitro* prior to adoptive transfer (Fig 6C) as well as *in vivo* after CAR T cells had infiltrated tumours (Fig EV3C). By contrast, cell surface levels of other chemokine receptors, including CXCR5, CCR7 and CCR5, were not altered *in vitro* (Fig 6D), or moderately increased *in vivo* after infiltrating tumours (Fig EV3C). The ligands for CXCR3 are increased in many tumours and associated with the intralesional accumulation of TILs and improved outcome (Slaney *et al*, 2014; Nagarsheth *et al*, 2017) and *Cxcl9* and *Cxcl10* (*Cxcl11* is not expressed in C57BL/6 mice; Sierro *et al*, 2007) were elevated in the HER‐2‐E0771 tumours analysed at 10 days after implantation (Fig 6E). Moreover, consistent with the potential for increased homing, we found that PTPN2‐deficient CXCR3^hi^ CAR T cells accumulated in HER‐2‐E0771 tumours within 3 days of adoptive transfer (Fig 6F), prior to any effects on tumour burden (Fig 4A).

To explore whether the increased cell surface CXCR3 might contribute to the increased homing and anti‐tumour activity of PTPN2‐deficient CAR T cells, we sought to correct the increased CXCR3 expression. CXCR3 is not detected in naïve T cells but is abundant in CD4^+^ T_H1_ cells and CD8^+^ cytotoxic T lymphocytes (CTLs). When CD8^+^ T cells are activated by a strong TCR stimulus and subsequently stimulated with IL‐2, they undergo differentiation into effectors and acquire CTL activity characterised by IFNγ and granzyme B expression (Malek *et al*, 2001; Pipkin *et al*, 2010). Our previous studies have shown that PTPN2 deficiency enhances the IL‐2‐induced generation of effectors from TCR crosslinked and activated T cells (Wiede *et al*, 2014b). Given that CAR T‐cell generation is reliant on TCR crosslinking (α‐CD3ε/α‐CD28) and stimulation with IL‐2 and IL‐7, we determined whether the enhanced LCK activation and increased downstream IL‐2 receptor and STAT‐5 signalling in PTPN2‐deficient CAR T cells might be responsible for the increased CXCR3 expression. To assess this, we took advantage of PTPN2‐deficient CD8^+^ CAR T cells that were heterozygous for *Lck* (*Lck*‐Cre;*Ptpn2* ^*fl*/*fl*^;*Lck* ^+/−^). Since IL‐2‐induced STAT‐5 signalling was only partially corrected in *Lck*‐Cre;*Ptpn2* ^*fl*/*fl*^;*Lck* ^+/−^ CAR T cells (Fig EV2F), we also crossed the *Lck*‐Cre;*Ptpn2* ^*fl*/*fl*^ mice onto the *Stat5* ^*fl*/+^ background so that we could independently correct the increased STAT‐5 signalling (Fig 6G and H). Cell surface CXCR3 levels were significantly albeit modestly reduced in *Lck* heterozygous CAR T cells (Fig 6G). By contrast, *Stat5* heterozygosity completely corrected the enhanced IL‐2‐ and IL‐15‐induced STAT‐5 signalling and almost completely corrected the increased CXCR3 (Fig 6G and H).

**Figure 7:**
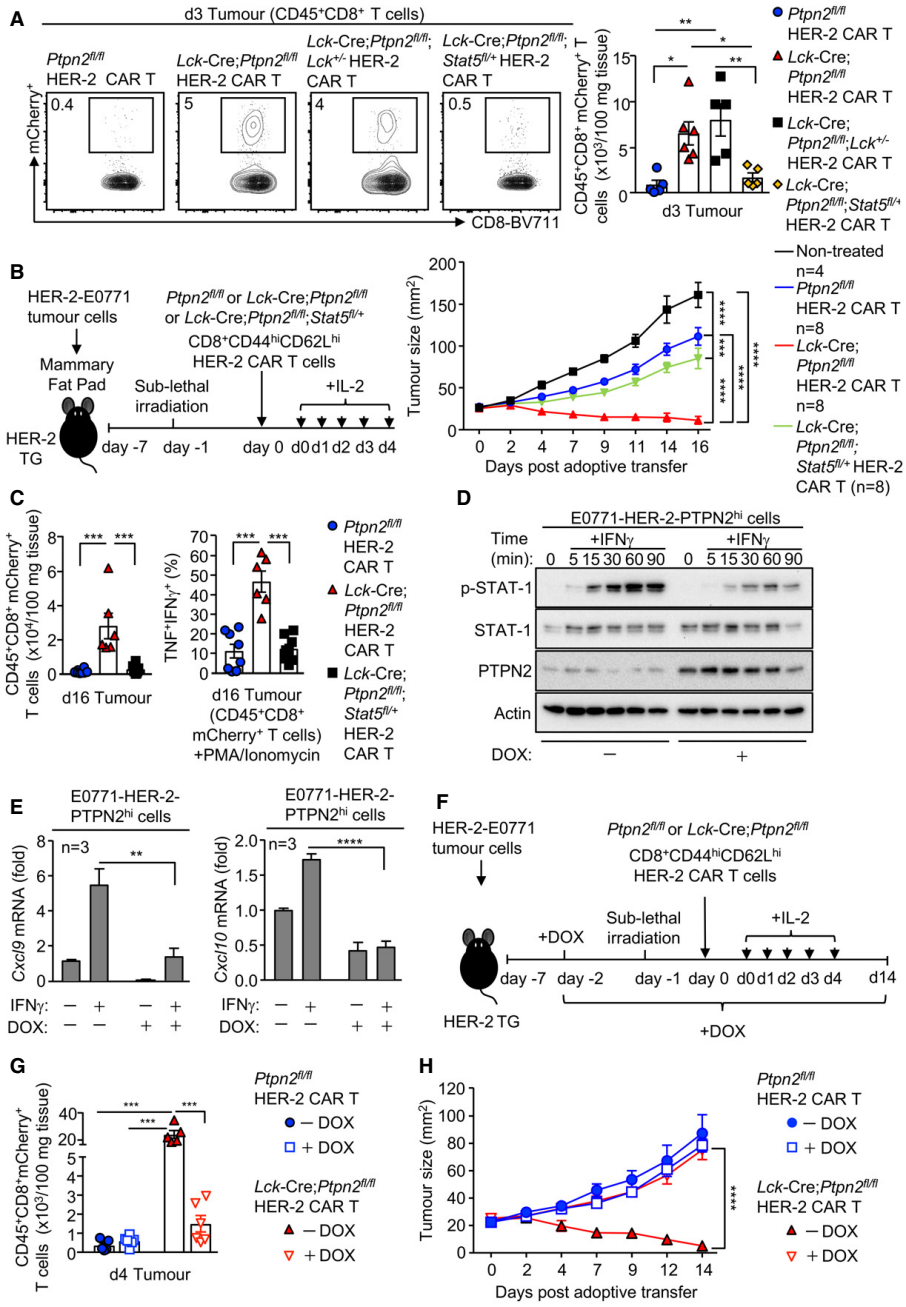
PTPN2 deficiency enhances CAR T‐cell efficacy in vivo by promoting STAT‐5‐mediating homing to CXCL9/10‐expressing tumours A–CHER‐2‐E0771 mammary tumours cells (2 × 10^5^) were injected into the fourth inguinal mammary fat pads of female HER‐2 TG mice. Six days after tumour injection, HER‐2 TG mice received total body irradiation (4 Gy) followed by the adoptive transfer of 6 × 10^6^ FACS‐purified CD8^+^CD62L^hi^CD44^hi^ central memory HER‐2 CAR T cells generated from *Ptpn2* ^*fl*/*fl*^, *Lck*‐Cre;*Ptpn2* ^*fl*/*fl*^, *Lck*‐Cre;*Ptpn2* ^*fl*/*fl*^;*Stat5* ^*fl*/+^ or *Lck*‐Cre;*Ptpn2* ^*fl*/*fl*^;*Lck* ^+/−^ splenocytes. Mice were injected with IL‐2 (50,000 IU/day) on days 0–4 after adoptive CAR T‐cell transfer and (B) monitored for tumour growth. (A, C) Lymphocytes were isolated from the tumours on (A) day 3 or (C) day 16 post‐adoptive transfer, and mCherry^+^CD45^+^CD8^+^ CAR T‐cell numbers were determined by flow cytometry. In (C), TILs were stained for intracellular IFNγ and TNF after PMA/ionomycin treatment.DHER‐2‐E0771 cells generated to inducibly overexpress PTPN2 in response to doxycycline (E0771‐HER‐2‐PTPN2^hi^) were pre‐incubated (24 h) with vehicle or doxycycline (DOX) subsequently stimulated with IFNγ for the indicated times. STAT‐1 Y701 phosphorylation (p‐STAT‐1) and PTPN2 levels were assessed by immunoblotting.E
*Cxcl9* and *Cxcl10* mRNA levels in vehicle versus DOX‐treated and IFNγ‐stimulated HER‐2‐E0771 cells were assessed by quantitative real‐time PCR.F–HE0771‐HER‐2‐PTPN2^hi^ mammary tumour cells (2 × 10^5^) were injected into the fourth inguinal mammary fat pads of female HER‐2 TG mice. Five days after tumour injection, mice were administered vehicle or DOX in drinking water followed by irradiation (4 Gy) on day 6 and the adoptive transfer of 6 × 10^6^ FACS‐purified central memory *Ptpn2* ^*fl*/*fl*^ versus *Lck*‐Cre;*Ptpn2* ^*fl*/*fl*^ HER‐2 CAR T cells. Mice were then injected with IL‐2 (50,000 IU/day) on days 0–4 post‐adoptive CAR T‐cell transfer, and (H) tumour growth was monitored. In (G), CD45^+^CD8^+^mCherry^+^ TILs were quantified by flow cytometry at day 4 post‐adoptive transfer.Data information: Representative flow cytometry profiles and results (means ± SEM) from two independent experiments are shown. In (A, E), significance was determined using 1‐way ANOVA test. In (B, G, H), significance was determined using 2‐way ANOVA test. In (C), significance was determined using 2‐tailed Mann–Whitney *U*‐test. *P < 0.05, **P < 0.01, ***P < 0.001, ****P < 0.0001.

Although CXCR3 is not transcribed by STAT‐5, there is evidence that STAT‐5 can drive the expression of the transcription factor T‐bet (T‐box transcription factors T‐box expressed in T cells) (Grange *et al*, 2013) which together with Eomes (eomesodermin) dictates CD8^+^ T‐cell differentiation and function. Indeed, T‐bet can drive the expression of CXCR3 (Taqueti *et al*, 2006) and has been shown to be important for the infiltration and anti‐tumour activity of cytotoxic CD8^+^ T cells (Zhu *et al*, 2010). Consistent with this, we found that PTPN2 deficiency in CAR T cells was associated with increased intracellular T‐bet expression that was corrected by *Stat5* heterozygosity (Fig EV3D and E). Strikingly, *Stat5* but not *Lck* heterozygosity prevented the increased homing of PTPN2‐deficient CAR T cells evident at 3 days post‐adoptive transfer (Fig 7A) and largely, albeit not completely, attenuated the ability of PTPN2‐deficient CAR T cells to suppress the growth solid tumours (Fig 7B). The repression of CAR T‐cell infiltration was also evident in resected tumours at day 16 with *Lck*‐Cre;*Ptpn2* ^*fl*/*fl*^;*Stat5* ^*fl*/+^ CAR T‐cell cytotoxicity markers (TNF, IFNγ; induced by PMA/ionomycin *ex vivo*) being reduced to those in *Ptpn2* ^*fl*/*fl*^ control CAR T cells (Fig 7C). Therefore, the promotion of STAT‐5 signalling might not only promote CXCR3 expression and the homing of CAR T cells to CXCL9/10‐expressing tumours, but also contribute to the acquisition of CTL activity probably through the induction of T‐bet and thereby CAR T‐cell function.

To complement these findings and further explore the extent to which the CXCR3/CXCL9/10/11 axis might contribute to the increased homing and efficacy of PTPN2‐deficient CAR T cells, we sought to repress the *Cxcl9/10* expression in HER‐2‐E0771 mammary tumours and assess the impact on the homing and function of PTPN2‐deficient CAR T cells (Fig 7D–H). As *Cxcl9/10* are transcriptional targets of STAT‐1 and PTPN2 dephosphorylates STAT‐1 to repress IFNγ‐induced STAT‐1‐mediated transcription (ten Hoeve *et al*, 2002; Gurzov *et al*, 2014; Manguso *et al*, 2017; Grohmann *et al*, 2018), we generated HER‐2‐E0771 cells in which PTPN2 could be inducibly overexpressed in response to doxycycline (Fig 7D). The inducible overexpression of PTPN2 not only repressed IFNγ‐induced p‐STAT‐1 (Fig 7D) and *Cxcl9/10* expression (Fig 7E), but most importantly also the recruitment of PTPN2‐deficient CAR T cells to HER‐2‐E0771 mammary tumours *in vivo* (Fig 7F–H). Importantly, the doxycycline‐inducible overexpression of PTPN2 in HER‐2‐E0771 cells and the decreased CAR T‐cell recruitment abrogated the ability of PTPN2‐deficient CAR T cells to suppress tumour growth (Fig 7H). Taken together, our findings are consistent with the efficacy of PTPN2‐deficient CAR T cells in solid tumours being attributed to (i) the increased LCK‐dependent activation of CAR T cells after antigen engagement, (ii) the LCK and STAT‐5‐dependent acquisition of CTL activity and (iii) the increased STAT‐5‐mediated and CXCR3‐dependent homing of PTPN2‐deficient CAR T cells to CXCL9/10‐expressing tumours.

### PTPN2‐deficient CAR T cells do not promote morbidity

**Figure EV4:**
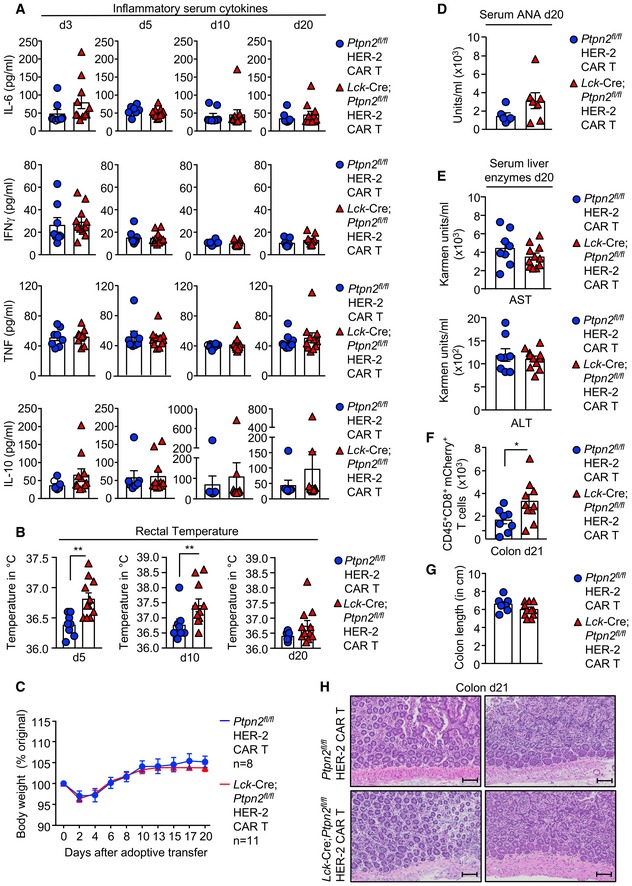
PTPN2 deficiency in CAR T cells does not result in autoimmunity HER‐2‐E0771 mammary tumour cells (2 × 10^5^) were injected into the fourth inguinal mammary fat pads of female HER‐2 TG mice. Six days after tumour injection, HER‐2 TG mice received total body irradiation (4 Gy) followed by the adoptive transfer of 20 × 10^6^ FACS‐purified CD8^+^CD44^hi^CD62L^hi^ central memory HER‐2 CAR T cells generated from *Ptpn2* ^*fl*/*fl*^ versus *Lck*‐Cre;*Ptpn2* ^*fl*/*fl*^ splenocytes. Mice were injected with IL‐2 (50,000 IU/day) on days 0–4 after adoptive CAR T‐cell transfer.
ASerum cytokines were determined with the BD CBA Mouse Inflammation Kit™.B, CBody core temperatures were measured using a mouse rectal probe, and (C) body weights were monitored.DSerum anti‐nuclear antibodies (ANA) were measured using a mouse anti‐nuclear antibodies Ig's (total IgA+G+M) ELISA Kit.ESerum AST and ALT activities were determined using a Transaminase II Kit.F, GLamina propria CD45^+^CD8^+^ donor CAR T‐cell numbers (F) and colon lengths (G) at 21 days post‐adoptive CAR T‐cell transfer.HColons at 21 days post‐CAR T‐cell transfer were fixed in formalin and processed for histology (haematoxylin and eosin) monitoring for tissue architecture and lymphocytic infiltrates. Scale bars: 100 μm.Data information: Representative results (means ± SEM) are shown from two independent experiments. In (B, F), significance was determined using 2‐tailed Mann–Whitney *U*‐test. *P < 0.05, **P < 0.01.

A potential complication of enhancing the function of CAR T cells is the development of systemic inflammation and autoimmunity (Bonifant *et al*, 2016; Yong *et al*, 2017). We found that the eradication of tumours by PTPN2‐deficient HER‐2 CAR T cells was not accompanied by systemic T‐cell activation and inflammation, as assessed by the unaltered number and activation of T cells in lymphoid and non‐lymphoid organs at 21 days post‐transfer (Appendix Fig S5A) and unaltered circulating pro‐inflammatory cytokines (Appendix Fig S5B) and lymphocytic infiltrates in non‐lymphoid tissues, including in the contralateral tumour‐negative mammary glands (Appendix Fig S5C). To further explore any impact of PTPN2 deficiency on the development of inflammatory disease, we increased the number of adoptively transferred CAR T cells from 6 × 10^6^ to 20 × 10^6^ and monitored for inflammation and autoimmunity. The increased CAR T‐cell numbers resulted in a more profound repression of tumour growth irrespective of PTPN2 status, but only PTPN2‐deficient CAR T cells eradicated tumours (Appendix Fig S5D). PTPN2 deficiency did not exacerbate systemic inflammation, as assessed by monitoring for lymphocytic infiltrates in non‐lymphoid tissues, including the lungs and livers (Appendix Fig S5E) or for circulating IL‐6, IFNγ, TNF and IL‐10 over time (Fig EV4A). Although PTPN2‐deficient CAR T cells increased core temperature as early as 5 days post‐adoptive transfer (Fig EV4B), this is to be expected for a developing immune response and this did not persist after tumours were cleared and did not affect body weight (Fig EV4B and C). In addition, the increased PTPN2‐deficient CAR T cells did not result in autoimmunity as reflected by the absence of circulating anti‐nuclear antibodies (Fig EV4D) and the lack of any overt tissue damage, including liver damage, as assessed by measuring the liver enzymes alanine transaminase (ALT) and aspartate transaminase (AST) in serum (Fig EV4E). Moreover, although PTPN2‐deficient CAR T cells were increased in the lamina propria (Fig EV4F), there were no signs of overt tissue damage (as assessed histologically) or colitis, as assessed by measuring colon length (Fig EV4G and H). Therefore, PTPN2‐deficient CAR T cells eradicate tumours without promoting systemic inflammation and immunopathologies.

Another potential complication of CAR T‐cell therapy is “on‐target off‐tumour” toxicities (Bonifant *et al*, 2016; Yong *et al*, 2017). Previous studies have shown that the WAP promoter in the HER‐2 TG mice drives HER‐2 in the lactating mammary gland and the cerebellum (Piechocki *et al*, 2003). Although PTPN2‐deficient CAR T cells were not evident in the contralateral tumour‐negative mammary glands, this is not unexpected as we used virgin mice in our studies. By contrast, the WAP promoter (Piechocki *et al*, 2003) drives HER‐2 expression in the cerebellum and we could detect HER‐2 throughout the cerebellum (Appendix Fig S6A) to similar levels seen in HER‐2‐E0771 mammary tumours (Appendix Fig S6B). Although we detected some CD3^+^mCherry^+^ CAR T cells surrounding the crus 1 of the ansiform lobule of the cerebellum, they were not detected in other regions (Appendix Fig S6C) and there was no evidence of overt cerebellar tissue damage (Appendix Fig S6D), as assessed on day 10 post‐adoptive transfer when PTPN2‐deficient CAR T cells were activated (Appendix Fig S6E). Consistent with this, PTPN2‐deficient CAR T cells did not result in overt morbidity up to 70 days post‐transfer and did not affect the cerebellar control of neuromotor function, as assessed in rotarod tests (Appendix Fig S6F), even when a greater number of CAR T cells were adoptively transferred. In part, the lack of toxicity may be due to CXCR3‐expressing CAR T cells being unable to efficiently home and infiltrate into the cerebellum, as the cerebellum expressed negligible levels of *Cxcl9* and *Cxcl10* when compared to the HER‐2‐E0771 tumours (Appendix Fig S6G). Therefore, targeting PTPN2 may not only enhance the antigen‐specific activation and function of CAR T cells, but also limit “on‐target off‐tumour” toxicities by driving the homing of CXCR3‐expressing CAR T cells to CXCL9/10/11‐expressing tumours. Taken together, our results demonstrate that PTPN2 deletion in murine CD8^+^ CAR T cells dramatically enhances their recruitment into the tumour site, as well as their antigen‐specific activation and ability to overcome the immunosuppressive tumour microenvironment to effectively suppress the growth of solid tumours without promoting overt morbidity.

### PTPN2 inhibition enhances the antigen‐specific activation of human CAR T cells

**Figure EV5:**
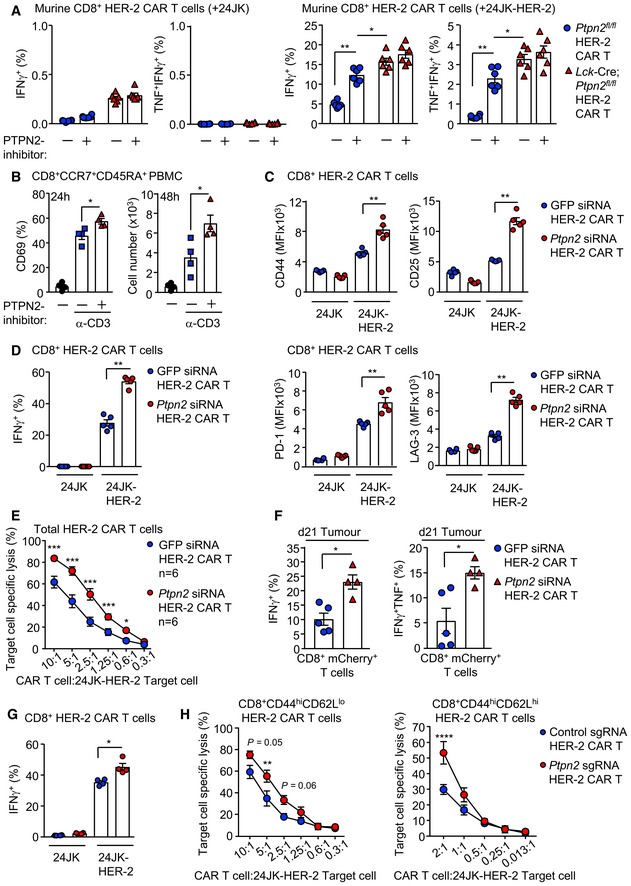
Targeting Ptpn2 with siSTABLE™ siRNAs or using CRISPR‐Cas9 RNP enhances tumour‐specific CAR T‐cell responses A
*Ptpn2* ^*fl*/*fl*^ versus *Lck*‐Cre;*Ptpn2* ^*fl*/*fl*^ CD8^+^ HER‐2 CAR T cells were treated with PTPN2‐inhibitor (+) or vehicle (−) followed by incubation with 24JK‐HER‐2 versus 24JK sarcoma cells. The proportion of CD8^+^IFNγ^+^ versus CD8^+^IFNγ^+^TNF^+^ CAR T cells was determined by flow cytometry.BHuman PBMCs were pretreated with PTPN2‐inhibitor (+) or vehicle (−) and stimulated with α‐CD3. MFIs for CD69 on CD8^+^CCR7^+^CD45RA^+^ T cells and CD8^+^CCR7^+^CD45RA^+^ T‐cell numbers were determined by flow cytometry.CHER‐2 CAR T cells transfected with GFP versus *Ptpn2* siSTABLE™ siRNAs were incubated with 24JK‐HER‐2 versus 24JK sarcoma cells. CD44, CD25, PD‐1 and LAG‐3 MFIs on CD8^+^ CAR T cells were determined by flow cytometry.DHER‐2 CAR T cells transfected with GFP versus *Ptpn2* siSTABLE™ siRNAs were incubated with 24JK‐HER‐2 versus 24JK sarcoma cells. The proportion of CD8^+^IFNγ^+^ CAR T cells was determined by flow cytometry.EHER‐2 CAR T cells transfected with GFP versus *Ptpn2* siSTABLE™ siRNAs were incubated with 5 μM CTV‐labelled (CTV^bright^) 24JK‐HER‐2 and 0.5 μM CTV‐labelled (CTV^dim^) 24JK sarcoma cells. Antigen‐specific target cell lysis (24JK‐HER‐2 versus 24JK response) was monitored for the depletion of CTV^bright^ 24JK‐HER‐2 cells by flow cytometry.FmCherry^+^ CAR T cells isolated from HER‐2‐E0771 tumours 21 days post‐adoptive transfer were assessed for the proportion of CD8^+^IFNγ^+^ versus CD8^+^IFNγ^+^TNF^+^ CAR T cells by flow cytometry.G, HHER‐2 CAR T cells were transfected with control versus *Ptpn2* sgRNAs plus Cas9 using the Lonza 4D‐Nucleofector to delete PTPN2 by CRISPR‐Cas9 RNP. (G) Control and PTPN2‐deleted HER‐2 CAR T cells were incubated with 24JK‐HER‐2 versus 24JK sarcoma cells and the proportion of CD8^+^IFNγ^+^ CAR T cells determined by flow cytometry. (H) Alternatively, FACS‐purified CD8^+^CD44^hi^CD62L^hi^ CAR T cells and CD8^+^CD44^hi^CD62L^lo^ CAR T control and PTPN2‐deleted HER‐2 CAR T cells were incubated with 5 μM CTV‐labelled (CTV^bright^) 24JK‐HER‐2 cells and 0.5 μM CTV‐labelled (CTV^dim^) 24JK sarcoma cells. Antigen‐specific target cell lysis (24JK‐HER‐2 versus 24JK response) was assessed by monitoring for the depletion of CTV^bright^ 24JK‐HER‐2 cells by flow cytometry.Data information: Representative results (means ± SEM) from at least two independent experiments are shown. In (A–D, F, G), significance was determined using 2‐tailed Mann–Whitney *U*‐test. In (E, H), significance was determined using 2‐way ANOVA test. *P < 0.05, **P < 0.01, ***P < 0.001, ****P < 0.0001.

**Figure 8:**
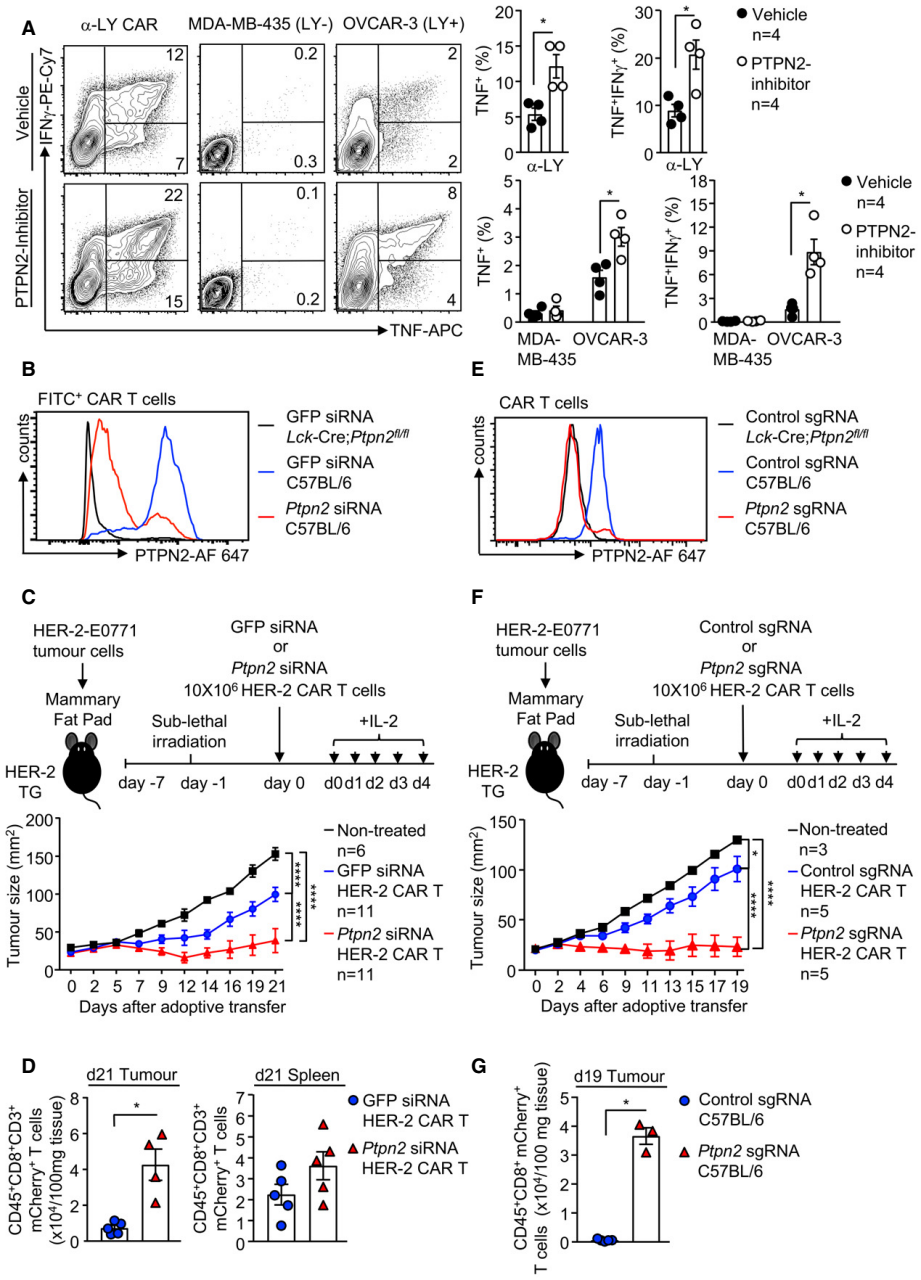
PTPN2 targeting enhances murine and human CAR T‐cell responses ACD8^+^ LY CAR T cells generated from human PBMCs were treated with PTPN2‐inhibitor (+) or vehicle (−) followed by incubation with plate‐bound α‐LY, LY‐negative MDA‐MB‐435 cells and LY‐expressing OVCAR‐3 cells. The proportion of IFNγ^+^ versus IFNγ^+^TNF^+^CD8^+^ CAR T cells was determined by flow cytometry.BHER‐2 CAR T cells generated from C57BL/6 and *Lck*‐Cre;*Ptpn2* ^*fl*/*fl*^ splenocytes were transfected with GFP versus *Ptpn2* siSTABLE™ FITC‐conjugated siRNAs, and intracellular PTPN2 levels were determined by flow cytometry.C, DHER‐2‐E0771 mammary tumour cells (2 × 10^5^) were injected into the fourth inguinal mammary fat pads of female HER‐2 TG mice. Six days after tumour injection, HER‐2 TG mice received total body irradiation (4 Gy) followed by the adoptive transfer of 10 × 10^6^ HER‐2 CAR T cells generated from C57BL/6 splenocytes transfected with GFP versus *Ptpn2* siSTABLE™ FITC‐conjugated siRNAs 2 days before adoptive CAR T‐cell transfer. Mice were injected with IL‐2 (50,000 IU/day) on days 0–4 after adoptive CAR T‐cell transfer, and (C) tumour growth was monitored. (D) CD45^+^CD3^+^CD8^+^mCherry^+^ CAR T cells numbers were determined in HER‐2‐E0771‐positive tumours and spleens by flow cytometry 21 days post‐adoptive transfer.EHER‐2 CAR T cells generated from C57BL/6 and *Lck*‐Cre;*Ptpn2* ^*fl*/*fl*^ splenocytes were transfected with Cas9 and control or *Ptpn2* sgRNAs using the Lonza 4D‐Nucleofector and after 2 days intracellular PTPN2 levels determined by flow cytometry.F, GHER‐2‐E0771 mammary tumour cells (2 × 10^5^) were injected into the fourth inguinal mammary fat pads of female HER‐2 TG mice. Six days after tumour injection, HER‐2 TG mice received total body irradiation (4 Gy) followed by the adoptive transfer of 10 × 10^6^ control HER‐2 CAR T cells or those in which PTPN2 had been deleted by CRISPR RNP. Mice were injected with IL‐2 (50,000 IU/day) on days 0–4 post‐adoptive CAR T‐cell transfer and (F) tumour growth monitored. (G) CD45^+^CD8^+^mCherry^+^ CAR T‐cell numbers were determined in HER‐2‐E0771 tumours by flow cytometry 19 days post‐adoptive transfer.Data information: Representative flow cytometry profiles and results (means ± SEM) from two independent experiments are shown. In (A, D, G), significance was determined using 2‐tailed Mann–Whitney *U*‐test. In (C, F), significance was determined using 2‐way ANOVA test. *P < 0.05, ****P < 0.0001.

To explore whether targeting PTPN2 in human T cells and CAR T cells might similarly promote their activation and function, we took advantage of a highly specific PTPN2 active site inhibitor, compound 8 (Zhang *et al*, 2009; Loh *et al*, 2011). Treatment of murine CD8^+^ HER‐2 CAR T cells with compound 8 *in vitro* increased their antigen‐specific cytotoxic potential to levels seen in *Lck*‐Cre;*Ptpn2* ^*fl*/*fl*^ HER‐2 CAR T cells, but had no additional effect on *Lck*‐Cre;*Ptpn2* ^*fl*/*fl*^ HER‐2 CAR T cells, consistent with the inhibitor acting specifically to inhibit PTPN2 and thereby activate CAR T cells upon engagement with specific tumour antigen (Fig EV5A). Importantly, we found that treatment of human peripheral blood mononuclear cells with compound 8 enhanced their activation (as assessed by the activation marker CD69) and the expansion of CD8^+^CCR7^+^CD45RA^+^CD69^+^ T cells in response to TCR ligation (α‐CD3ε) (Fig EV5B). To assess whether targeting of PTPN2 might enhance the cytotoxic potential of human CAR T cells, we took advantage of human CAR T cells targeting the Lewis Y (LY) antigen (Westwood *et al*, 2008; Peinert *et al*, 2010) that is overexpressed in many human cancers, including 80% of lung adenocarcinomas, 25% of ovarian carcinomas and 25% of colorectal adenocarcinomas (Fig 8A). Treatment of human LY CAR T cells with compound 8 significantly enhanced their cytotoxic potential, as assessed by the expression of IFNγ and TNF, in response to CAR crosslinking (with α‐LY) or engagement with LY‐expressing human ovarian (OVCAR‐3) carcinoma cells, but not melanoma cells (MDA‐MB‐435) that do not express LY (Fig 8A). Taken together, these results are consistent with PTPN2 targeting increasing the potential therapeutic efficacy of human CAR T cells as seen in our pre‐clinical models.

### PTPN2 knockdown or deletion enhances the therapeutic efficacy of CAR T cells

Whole‐body, T‐cell‐ or hematopoietic compartment‐specific PTPN2 deletion in mice results in systemic inflammation, overt autoreactivity and morbidity (You‐Ten *et al*, 1997; Wiede *et al*, 2011, 2012, 2017b, 2019). The potential for inflammatory complications, including cytokine release syndrome, precludes the utility of PTPN2 inhibitors for systemic therapy and the promotion of T‐cell‐mediated anti‐tumour immunity. Accordingly, we sought alternate ways by which to target PTPN2 in the context of adoptive cell therapy. To this end, we first knocked down *Ptpn2* in murine CAR T cells by RNA interference using nuclease resistant siRNA duplexes (siSTABLE™) that efficiently knockdown genes for prolonged periods (Figs 8B–D and EV5C–F). HER‐2 CAR T cells were transfected with GFP‐ or *Ptpn2*‐specific siSTABLE™ siRNAs 2 days prior to adoptive cell therapy; PTPN2 was knocked down in approximately one‐third of total HER‐2 CAR T cells as assessed by flow cytometry (Fig 8B) using validated antibodies (Wiede *et al*, 2014a). *Ptpn2* knockdown enhanced the tumour antigen‐specific activation/cytotoxic potential and killing capacity of HER‐2 CAR T cells *ex vivo* (Fig EV5C–E) and markedly repressed the growth of HER‐2‐E0771 mammary tumours *in vivo* (Fig 8C). The repression of tumour growth was accompanied by the significant infiltration of mCherry^+^ CD8^+^ HER‐2 CAR T cells into tumours (Fig 8D); infiltrating HER‐2 T cells exhibited increased cytotoxic capacity (as assessed by IFNγ and TNF expression after PMA/ionomycin treatment *ex vivo*) (Fig EV5F). By contrast, mCherry^+^ CD8^+^ HER‐2 CAR T‐cell numbers in the spleen were not affected by *Ptpn2* knockdown (Fig 8D). Therefore, the transient repression of PTPN2, even in a fraction of adoptively transferred CAR T cells, is sufficient to significantly enhance their activity/cytotoxicity and efficacy *in vivo*.

An alternate approach by which to target PTPN2 in T cells is through CRISPR‐Cas9 genome editing (Kim *et al*, 2014). In particular, we took advantage of Cas9 ribonucleoprotein (RNP)‐mediated gene editing to effectively delete PTPN2 in CAR T cells. This plasmid‐free approach allows for efficient but transient genome editing *ex vivo* so that resultant CAR T cells do not overexpress Cas9 and do not elicit immunogenic responses post‐adoptive transfer. To this end, we transfected total CAR T cells with recombinant nuclear‐localised Cas9 pre‐complexed with short guide (sg) RNAs capable of directing Cas9 to the *Ptpn2* locus (Figs 8E–G and EV5G and H). sgRNAs targeting the *Ptpn2* locus completely ablated PTPN2 protein in HER‐2 CAR T cells (Fig 8E) and enhanced their antigen‐specific activation/cytotoxic potential (assessed by IFNγ production) (Fig EV5G) and their capacity to specifically kill HER‐2‐expressing 24JK cells *ex vivo* (Fig EV5H). Importantly, we found that *Ptpn2* deletion led to the effective eradication of HER‐2‐E0771 mammary tumours (Fig 8F) and this was accompanied by the increased infiltration of mCherry^+^ HER‐2 CAR T cells into HER‐2‐E0771 mammary tumours (Fig 8G) *in vivo*. These results demonstrate that CRISPR‐Cas9 genome editing can be used to efficiently ablate PTPN2 to enhance the therapeutic efficacy of CAR T cells in solid cancer.

## Discussion

Approaches aimed at harnessing host immunity to destroy tumour cells have revolutionised cancer therapy (Pardoll, 2012; Ribas & Wolchok, 2018). Such approaches have relied on the targeting of immune checkpoints, such as PD‐1, to alleviate inhibitory constraints on T‐cell‐mediated anti‐tumour immunity in immunogenic tumours, or alternatively on adoptive T‐cell therapy, especially that employing CAR T cells (Pardoll, 2012; Yong *et al*, 2017; Ribas & Wolchok, 2018). Although the latter does not require pre‐existing anti‐tumour immunity, there are significant hurdles limiting efficacy and prohibiting the widespread utility of CAR T cells in the treatment of solid tumours (Yong *et al*, 2017). These include inefficient CAR T‐cell homing and infiltration into solid tumours and inadequate CAR T‐cell activation in the solid tumour microenvironment, which can be overtly immunosuppressive (Yong *et al*, 2017). Like PD‐1 (Nishimura *et al*, 1999; Wang *et al*, 2005), PTPN2 is fundamentally important in mediating T‐cell tolerance in mice and humans (Consortium WTCC, 2007, Long *et al*, 2011; Wiede *et al*, 2011, 2014a,b, 2019). This is underscored by the striking phenotype similarities between PTPN2‐deficient mice and those null for PD‐1 (You‐Ten *et al*, 1997; Nishimura *et al*, 1999; Wang *et al*, 2005; Wiede *et al*, 2011, 2012, 2019). Consistent with this, our studies herein demonstrate that the deletion of PTPN2 in T cells enhances cancer immunosurveillance and the anti‐tumour activity of adoptively transferred T cells. In particular, our studies demonstrate that the deletion of PTPN2 not only drives the homing of CAR T cells to solid tumours, but also their activation to eradicate tumours in an otherwise immunosuppressive tumour microenvironment. Therefore, targeting PTPN2 may provide a means for enhancing the anti‐tumour activity of T cells and extending the utility of CAR T cells beyond haematological malignancies to solid cancers.

A recent CRISPR loss‐of‐function screen in tumour cells identified PTPN2 as a top‐hit for the recruitment of T cells and the sensitisation of tumours to anti‐PD‐1 therapy (Manguso *et al*, 2017; Wiede & Tiganis, 2017). This was reliant on PTPN2 deficiency driving the IFNγ‐induced and STAT‐1‐mediated expression of antigen‐presentation pathway genes and T‐cell chemoattractants, such as *Cxcl9* in tumour cells (Manguso *et al*, 2017). In humans, *CXCL9* expression is generally associated with increased CD8^+^ T‐cell infiltrates and improved overall survival and response to chemotherapy (Nagarsheth *et al*, 2017). Our own studies indicate that the deletion of PTPN2 in CAR T cells drives the expression of CXCR3 and the trafficking of CAR T cells to CXCL9/10‐expressing mammary tumours. Indeed, we demonstrated that the homing and efficacy of PTPN2‐deficient CAR T cells was reliant on tumours expressing STAT‐1‐driven CXCR3 chemokines. Thus, PTPN2‐deficient CAR T cells might be especially effective against tumours such as oestrogen receptor‐negative and triple‐negative breast cancers (Shields *et al*, 2013) or lung cancers (Feng *et al*, 2017) that have low PTPN2 levels. However, it is important to note that a variety of human tumours, including for example breast, colon and ovarian cancers, express CXCL9/10/11 or do so after chemotherapy (Denkert *et al*, 2010; Sistigu *et al*, 2014; Au *et al*, 2016; Bronger *et al*, 2016; Loi *et al*, 2016; Nagarsheth *et al*, 2017; Opzoomer *et al*, 2019).

Although it may be possible to target PTPN2 systemically with drugs to enhance T‐cell and CAR T‐cell responses and potentially achieve synergistic effects through the targeting of PTPN2 in both tumour cells and T cells/CAR T cells, there are several important considerations. First, the currently available PTPN2 inhibitors target the active site of the enzyme and are therefore hydrophilic and have difficulties with bioavailability and pharmacokinetics (Zhang *et al*, 2015). Second, we have shown that the inducible deletion of PTPN2 in the hematopoietic compartment of adult non‐autoimmune‐prone C57BL/6 mice is sufficient to promote the development of systemic inflammation and autoimmunity (Wiede *et al*, 2017b), whereas PTPN2 deletion in T cells in autoimmune‐prone NOD1 mice markedly accelerates type 1 diabetes onset, as well as other autoimmune and inflammatory disorders, including colitis (Wiede *et al*, 2019). Therefore, any systemic targeting of PTPN2 might exacerbate immune complications frequently associated with immunotherapy, including cytokine release syndrome that can be life‐threatening in CAR T‐cell therapy (Yong *et al*, 2017). Third, we and others have shown that the deletion of PTPN2 in some solid tumours can enhance tumorigenicity (Shields *et al*, 2013; Lee *et al*, 2017; Grohmann *et al*, 2018). For example, PTPN2 deletion in the liver can facilitate the STAT‐3‐dependent development of hepatocellular carcinoma in obesity (Grohmann *et al*, 2018). Accordingly, we propose that the targeting of PTPN2 in adoptively transferred CAR T cells using stabilised siRNAs or CRISPR RNP genome editing might be a safer and ultimately more effective means for enhancing the clinical efficacy of CAR T cells in solid tumours.

Our studies demonstrate that the deletion of PTPN2 enhances the function of CAR T cells by promoting antigen‐induced LCK activation and cytokine‐induced STAT‐5 signalling. We and others have shown that LCK and STAT‐5 can serve as *bona fide* substrates of PTPN2 in thymocytes/T cells (Simoncic *et al*, 2002; Tiganis & Bennett, 2007; Wiede *et al*, 2011, 2014a, 2017a; Gurzov *et al*, 2014). In this study, we found that correcting the increased LCK or STAT‐5 signalling diminished the efficacy of PTPN2‐deficient CAR T cells. Our studies indicate that the induction of LCK is necessary for the increased antigen‐induced CAR T‐cell activation, IL‐2/IL‐15 receptor subunit (CD25, CD122 and CD132) expression and cytotoxicity of PTPN2‐deficient CAR T cells, whereas the concomitant direct promotion of STAT5 phosphorylation and cytokine signalling might not only facilitate the acquisition of CTL activity, but also promote homing to CXCL9/10/11‐expressing tumours through the induction of CXCR3. IL‐2‐induced STAT‐5 signalling is necessary for the differentiation of activated CD8^+^ T cells into effectors and the acquisition of CTL activity (Malek *et al*, 2001; Pipkin *et al*, 2010). Although the precise mechanism by which STAT‐5 promotes CXCR3 in PTPN2‐deficient CAR T cells remains unclear, CXCR3 is elevated in effector CD8^+^ T cells and previous studies have shown that STAT‐5 can drive T‐bet and Eomes expression (Grange *et al*, 2013). Consistent with this, our studies demonstrate that the elevated STAT‐5 signalling in PTPN2‐deficient CAR T cells was also associated with increased T‐bet expression. Importantly, T‐bet can promote the expression of CXCR3 (Taqueti *et al*, 2006) and both T‐bet and Eomes have been shown to be important for the tumour infiltration and anti‐tumour activity of cytotoxic CD8^+^ T cells (Zhu *et al*, 2010). Moreover, in the context of viral infection, CXCR3 on CD8^+^ T cells facilitates T‐cell homing to infected tissue and the ability of T cells to locate and eliminate infected cells (Hickman *et al*, 2015). In our studies, we found STAT‐5 and the resultant increased CXCR3 promoted the homing of PTPN2‐deficient CAR T cells to CXCL9/10‐expressing mammary tumours within 3 days of adoptive transfer. By contrast, significant PTPN2‐deficient CAR T cells were not detected in the HER‐2^+^ cerebellum that did not express CXCL9/10. As systemic inflammation, tissue damage, autoimmunity and morbidity were not evident in HER2 TG mice, despite abundant HER2 expression in the cerebellum, we conclude that the enhancement of STAT5 signalling limits “on‐target off‐tumour” toxicities by promoting the specific homing of CAR T cells to CXCL9/10‐expressing tumours. Although other tissues such as the gut epithelium also express CXCR3 chemokines (Dwinell *et al*, 2001), and comparatively small increases in PTPN2‐deficient CAR T cells were detected in the colon (lamina propria), this was not accompanied by overt tissue damage or colitis. This is in keeping the lack or HER2 expression in the colon in HER2 TG mice and our findings demonstrating that PTPN2 deficiency only promotes the antigen‐induced activation of CAR T cells. Nonetheless, going forth, it would remain important to carefully select the tumour‐associated antigen targeted by CAR T cells to limit “on‐target off‐tumour” toxicities.

The results of this study have established the importance of PTPN2 in T‐cell immunosurveillance and defined a novel target for bolstering the anti‐tumour activity of T cells, especially in the context of adoptive T‐cell therapy. In particular, our findings have defined an approach by which to enhance the efficacy of CAR T cells and extend their utility to the treatment of solid tumours without promoting systemic inflammation/autoimmunity and morbidity.

## Materials and Methods

### Cell lines and mice

The C57BL/6 mouse mammary carcinoma cell line E0771 (a gift from Robin Anderson, Peter MacCallum Cancer Centre) (Johnstone *et al*, 2015) and the C57BL/6 mouse sarcoma cell line 24JK (a gift from Patrick Hwu, NIH, Bethesda, Maryland, USA) (Shiloni *et al*, 1993) were genetically engineered to express truncated human HER‐2 (HER‐2‐E0771) as described previously (Kershaw *et al*, 2004). The C57BL/6 mouse mammary tumour cell line AT‐3 was genetically engineered to express chicken ovalbumin (AT‐3‐OVA) and has been previously described (Loi *et al*, 2013). The GP+E86 packaging line was generated as previously described (Darcy *et al*, 2000). The human epithelial adenocarcinoma cell line OVCAR‐3 (ATCC^®^HTB‐161™) and the human melanoma cell line MDA‐MB‐435 (ATCC^®^HTB‐219™) were obtained from the ATCC, Manassas, Virginia, USA. HER‐2‐E0771 cells were engineered to inducibly overexpress murine PTPN2 (HER‐2‐E0771‐PTPN2^hi^) in response to doxycycline using the Tet‐On 3G Inducible Expression System according to the manufacturer's instructions (Clontech). Tumour cells were cultured in RPMI 1640 (HER‐2‐E0771, OVCAR‐3, MDA‐MB‐435) or high‐glucose DMEM (AT3‐OVA) supplemented with 10% FBS, l‐glutamine (2 mM), penicillin (100 units/ml)/streptomycin (100 μg/ml), MEM non‐essential amino acids (0.1 mM), sodium‐pyruvate (1 mM), HEPES (10 mM) and 2‐mercaptoethanol (50 μM).

Mice were maintained on a 12‐h light–dark cycle in a temperature‐controlled high barrier facility with free access to food and water. Six‐ to 10‐week‐old female Ly5.1 (B6.SJL‐*Ptprc* ^*a*^ *Pepc* ^*b*^/BoyJ) and human HER‐2 transgenic (TG) recipient mice and 6‐ to 8‐week‐old female donor mice were used for adoptive transfers. For *ex vivo* experiments, either male or female mice were used. Aged‐ and sex‐matched littermates were used in all experiments. *Ptpn2* ^*fl*/*fl*^ and *Lck*‐Cre;*Ptpn2* ^*fl/fl*^ mice and the corresponding OT‐1 TCR transgenic mice were described previously (Wiede *et al*, 2011). *p53* ^+/−^ (C57BL/6) mice have been described previously (Jacks *et al*, 1994) and were a gift from Prof. Andreas Strasser (WEHI, Melbourne, Australia). *Ptpn2* ^*fl*/*fl*^;*p53* ^+/−^ and *Lck*‐Cre;*Ptpn2* ^*fl*/*fl*^ *;p53* ^+/−^ mice were generated by crossing *p53* ^+/−^ (C57BL/6) with *Lck*‐Cre;*Ptpn2* ^*fl*/*fl*^ mice. B6.129S2‐*Lck*<tm1Mak>/J mice were a gift from Dr Andre Veillette (McGill University, Montreal) and were bred with *Lck*‐Cre;*Ptpn2* ^*fl*/*fl*^ mice to generate *Lck*‐Cre;*Ptpn2* ^*fl*/*fl*^;*Lck* ^+/−^ mice. Ly5.1 and C57BL/6 mice were purchased from the WEHI Animal Facility (Kew, Australia), and human HER‐2 (C57BL/6) transgenic (TG) mice were bred and maintained at the Peter MacCallum Cancer Centre.

### Materials

For cell stimulation, anti‐CD3ε (clone 145‐2C11) and anti‐CD28 (clone 37.51) antibodies were purchased from BD Biosciences. Antibodies against p‐(Y701) STAT1 (clone 58D6), p‐(Y694) STAT5 (D47E7) XP^®^ and STAT1 were from Cell Signaling. Anti‐actin (clone ACTN05) was purchased from Thermo Fisher Scientific. The mouse antibody against PTPN2 (clone 6F3) was provided by M. Tremblay (McGill University). For immunofluorescence staining and immunohistochemistry, rabbit anti‐CD3ε and mouse anti‐HER‐2 from Abcam were used. Recombinant human IL‐2, murine IL‐7 and IL‐15 used for T‐cell stimulation or IFNγ used for stimulating tumour cells were purchased from the NIH or PeproTech, respectively. RetroNectin was purchased from Takara, Dnase I and doxycycline from Sigma‐Aldrich. The mouse anti‐nuclear antibodies Ig's (total IgA+G+M) ELISA Kit (Alpha Diagnostic Int.) and the Transaminase II Kit (Wako Pure Chemicals) were used according to the manufacturer's instructions. FBS was purchased from Thermo Scientific; Dulbecco‐Phosphate‐Buffered Saline (D‐PBS), RPMI 1640, DMEM, MEM non‐essential amino acids and sodium‐pyruvate were from Invitrogen, and collagenase type IV was purchased from Worthington Biochemical.

### Flow cytometry

Single‐cell suspensions from spleen, lymph nodes and hepatic lymphocytes were obtained as previously described (Wiede *et al*, 2011). For the detection of intracellular cytokines, cells were fixed and permeabilised with the BD Cytofix/Cytoperm kit according to the manufacturer's instructions. For the detection of intracellular FoxP3 and T‐bet, the Foxp3/Transcription Factor Staining Buffer Set (eBioscience) was used according to the manufacturer's instructions. For the detection of intracellular p(Y418)‐SFK and PTPN2, cells were stained and prepared for flow cytometry as previously described (Wiede *et al*, 2014a).

For the detection of serum cytokines, either the LEGENDplex T_h_ Cytokine Panel™ from BioLegend or the BD CBA Mouse Inflammation Kit™ was used according to the manufacturers’ instructions.

Cells were stained with the specified antibodies on ice for 30 min and analysed using a LSRII, Fortessa, Symphony (BD Biosciences) or CyAn™ ADP (Beckman Coulter).

For FACS sorting, cells were stained in 15‐ml Falcon tubes (BD Biosciences) for 30 min on ice and purified using either a BD Influx cell sorter, or the BD FACSAria II, BD FACSAria Fusion 3 or BD FACSAria Fusion 5 instruments.

Data were analysed using FlowJo8.7 or FlowJo10 (Tree Star Inc.) software. For cell quantification, a known number of Calibrite™ Beads (BD Biosciences) or Nile Red Beads (ProsiTech) or Flow‐Count Fluorospheres (Beckman Coulter) were added to samples before analysis.

The following antibodies from BD Biosciences, BioLegend or eBioscience were used for flow cytometry: Phycoerythrin (PE) or peridinin–chlorophyll cyanine 5.5 (PerCP‐Cy5.5)‐conjugated CD3 (145‐2C11); PerCP‐Cy5.5 or phycoerythrin–cyanine 7 (PE‐Cy7)‐conjugated CD4 (RM4‐5); BV711, Pacific Blue‐conjugated (PB), allophycocyanin (APC)‐Cy7 or APC‐conjugated CD8 (53‐6.7); PerCP‐Cy5.5, PE or APC‐Cy7‐conjugated CD25 (P61); Fluorescein isothiocyanate (FITC), V450, Alexa Fluor 700 or PE‐Cy7‐conjugated CD44 (IM7); APC‐conjugated CD45 (30‐F11); V450, APC, Alexa Fluor 488 or PE‐conjugated CD45.1 (Ly5.1; A20); FITC, PB or PerCP‐Cy5.5‐conjugated CD45.2 (Ly5.2; 104); APC‐conjugated CD45R (B220; RA3‐6B2); PE‐Cy7, APC or PE‐conjugated CD62L (Mel‐14); PE‐conjugated CD122 (TM‐β1); PE‐conjugated CD132 (4G3); Alexa Fluor 647‐conjugated CD183 (CXCR3‐173); PerCP‐eFluo710‐conjugated CD185 (CXCR5; SPRCL5); PE‐Cy7 conjugated CD197 (CCR7; 4B12); PE‐conjugated CD223 (LAG‐3; C9B7W); PE‐Cy7‐conjugated CD279 (PD‐1, RMP1‐14); FITC‐conjugated CD11b (M1/70); APC‐Cy7‐conjugated Ly6C (AL‐21); PE‐conjugated Ly‐6G (clone 1A8); APC‐conjugated F4/80 (BM8); PE‐conjugated IL‐10 (JES5‐16E3), PE‐conjugated Nur77 (12.14); PE‐Cy7‐conjugated IFNγ (XMG1.2); FITC or APC‐conjugated TNF (MP6‐XT22); Alexa Fluor 647‐conjugated Granzyme B (GB11); PE‐Cy7‐conjugated TCR‐Vα2 (B20.1); eFluor 660‐conjugated p(Y418)‐Src (SC1T2M3); Alexa Fluor 647‐conjugated T‐bet (4B10); and V450‐conjugated FoxP3 (clone MF23).

The following antibodies from Miltenyi Biotec were used for flow cytometry: PE‐conjugated CD197 (CCR7; REA108); VioBright FITC‐conjugated CD8 (BW135/80); and VioBlue‐conjugated CD45RA (REA1047).

### Generation of murine CAR T cells

Splenocytes were isolated from murine spleens by mechanical disruption, and contaminating red blood cells were removed by incubation with 1 ml of Blood Cell Lysing Buffer Hybri‐Max™ (Sigma‐Aldrich) for 7 min at room temperature per spleen. Lymphocytes (0.5 × 10^7^/ml) were cultured overnight with anti‐CD3ε (0.5 μg/ml) and anti‐CD28 (0.5 μg/ml) in the presence of 100 IU/ml human IL‐2 and 0.2 ng/ml murine IL‐7 in complete T‐cell medium [RPMI supplemented with 10% FBS, l‐glutamine (2 mM), penicillin (100 units/ml)/streptomycin (100 μg/ml), non‐essential amino acids, Na‐pyruvate (1 mM), HEPES (10 mM) and 2‐mercaptoethanol (50 μM)]. Dead cells were removed by Ficoll centrifugation (GE Healthcare) according to the manufacturer's instructions. Retrovirus encoding a second‐generation chimeric antibody receptor (CAR) consisting of an extracellular scFv‐anti‐human HER‐2, a membrane proximal CD8 hinge region and the transmembrane and the cytoplasmic signalling domains of CD28 fused to the cytoplasmic region of CD3ζ (scFv‐anti‐HER‐2‐CD28‐ζ) was obtained from the supernatant of the GP+E86 packaging line as described previously (Haynes *et al*, 2002). Retroviral supernatant was added to non‐treated RetroNectin‐coated (10 μg/ml) 6‐well plates (Takara Bio) and spun for 30 min (1,200× g) at room temperature. T cells were resuspended in 1 ml of additional retroviral‐containing supernatant supplemented with IL‐2 and IL‐7 and then added to the RetroNectin‐coated plates to a final volume of 5 ml/well with a final T‐cell concentration of 1 × 10^7^ per well. T cells were spun for 90 min (1,200× *g*) at room temperature and incubated overnight before the second viral transduction. T cells were maintained in IL‐2‐ and IL‐7‐containing media and cells used at days 7–8 after transduction.

### Generation of human CAR T cells

Human peripheral blood mononuclear cells (PBMCs) were isolated from normal donor buffy coats. PBMCs were stimulated with anti‐human CD3 (OKT3; 30 ng/ml, Ortho Biotech) and human IL‐2 (600 IU/ml) in complete T‐cell medium. Retrovirus encoding a second‐generation chimeric antibody receptor (CAR) consisting of an extracellular scFv‐anti‐human LeY domain, a membrane proximal CD8 hinge region and the transmembrane and the cytoplasmic signalling domains of CD28 fused to the cytoplasmic region of CD3ζ was obtained from the supernatant of the PG13 packaging cell line and has been described previously (Westwood *et al*, 2008; Peinert *et al*, 2010). Retroviral supernatant (5 ml) was added to non‐treated RetroNectin‐coated (10 μg/ml) 6‐well plates (Takara Bio) and incubated for 4 h at 37°C. Supernatant was removed, and 2.5 × 10^6^ T cells were incubated overnight in 5 ml of complete T‐cell medium supplemented with human IL‐2 before the second viral transduction. At day 7 post retroviral transduction, human CAR T cells were sorted for CD34^+^ (CD34 MicroBead Kit UltraPure, MACS, Miltenyi Biotec) CAR T cells and expanded for up to 15 days in the presence of human IL‐2. The expression of the chimeric receptor was determined by staining with Alexa Flour 647‐conjugated anti‐3S193 idiotype [supplied by Ludwig Institute for Cancer Research (LICR)], and truncated CD34 was detected with PE‐conjugated anti‐human CD34 (BioLegend). Cell surface phenotyping of transduced cells was determined by staining with BV785‐conjugated anti‐human CD3 (UCHT1, BioLegend), BV605‐conjugated anti‐human CD4 (OKT‐4, BioLegend) and PE‐Cy7‐conjugated anti‐human CD8 (SK1, BioLegend).

### Assessment of cytokine production in human CAR T cells

CAR T cells (2 × 10^5^) generated from human PBMC were incubated with Lewis Y‐expressing OVCAR‐3 cells (1 × 10^5^) or Lewis Y‐negative MDA‐MB‐435 cells (1 × 10^5^) or plate‐bound anti‐LY (1 μg/ml; Cell Therapies Pty Ltd, Peter MacCallum Cancer Centre) for 6 h at 37°C in complete T‐cell medium. GolgiPlug™ and GolgiStop™ were added 3 h before cells were processed for surface and intracellular staining for CD4 (BV785‐conjugated anti‐human; OKT‐4, BioLegend), CD8 (APC‐H7‐conjugated anti‐human; SK1, BD Bioscience), IFNγ (PE‐Cy7‐conjugated anti‐human; B27, BioLegend) and TNF (APC‐conjugated anti‐human TNF; MAb11; BioLegend) by flow cytometry.

### Cytokine signalling in CAR T cells

CAR T cells generated from mouse splenocytes were incubated with plate‐bound anti‐CD3ε for 24 h at 37°C in complete T‐cell medium. Cells were washed twice and rested in RPMI/1% FBS for 1 h. Cells were stimulated for the indicated time points with mouse recombinant IL‐2 (5 or 10 ng/ml) or IL‐15 (5 or 10 ng/ml) in 100 μl RPMI/1 %FBS. Cells were processed for intracellular p(Y694)‐STAT‐5 detection by flow cytometry as previously described (Wiede *et al*, 2014a).

### CAR T‐cell proliferation

CAR T cells generated from mouse splenocytes were stained with fluorochrome‐conjugated antibodies for CD8, CD62L and CD44 and sorted for CD8^+^CD44^hi^CD62L^hi^ CAR T cells. For the assessment of CAR T‐cell proliferation by CellTrace™ Violet (CTV; Molecular Probes, Thermo Fisher Scientific) dilution, purified CD8^+^CD44^hi^CD62L^hi^ CAR T cells were incubated with CTV in D‐PBS supplemented with 0.1% (v/v) BSA at a final concentration of 2 μM for 10 min at 37°C. Cells were then washed three times with D‐PBS supplemented with 10% (v/v) FBS. CAR T cells (2 × 10^5^) were incubated with HER‐2‐expressing or HER‐2‐negative 24JK sarcoma cells (1 × 10^5^) in complete T‐cell medium.

For the assessment of CAR T‐cell proliferation after anti‐CD3ε stimulation, purified CD8^+^CD44^hi^CD62L^hi^ CAR T cells were incubated with plate‐bound anti‐CD3ε for 24 h at 37°C in complete T‐cell medium. Pre‐activated CAR T Cells (2 × 10^5^) were labelled with 5 μM CTV and incubated with HER‐2‐expressing or HER‐2‐negative 24JK sarcoma cells (1 × 10^5^) in complete T‐cell medium. At various time points, proliferating cells were harvested and CTV dilution was monitored by flow cytometry. For cell quantification, Nile Red Beads (ProsiTech) or Flow‐Count Fluorospheres (Beckman Coulter) were added to samples before analysis.

### PTPN2‐inhibition studies

T cells were incubated rocking on an orbital shaker with PTPN2‐inhibitor compound 8 (100 nM) or vehicle (DMSO 1% v/v) in serum‐free RPMI 1640 for 1 h at 37°C. Cells were washed three times with complete T‐cell medium and processed for T‐cell activation studies.

### Treatment of HER‐2‐E0771 tumour‐bearing HER‐2 TG mice

Female human HER‐2 TG mice were anaesthetised with Ketamine (100 mg/kg) and Xylazil (10 mg/kg) (Troy Laboratories) and injected orthotopically with 2 × 10^5^ HER‐2‐E0771 cells resuspended in 20 μl D‐PBS into the fourth mammary fat pad. At day 6 post‐tumour cell injection, human HER‐2 TG mice were pre‐conditioned with total body irradiation (4 Gy) prior to the adoptive transfer of 6 × 10^6^ FACS‐purified CD8^+^CD44^hi^CD62L^hi^ CAR T cells. Mice were treated with 50,000 IU IL‐2 on days 0–4 after T‐cell transfer.

### Treatment of B16.F10‐OVA tumour‐bearing mice

Ly5.1 mice were anaesthetised with Ketamine (100 mg/kg) and Xylazil (10 mg/kg) (Troy Laboratories). The flanks of the mice were shaved using clippers and depilated with Veet (Reckitt Benckiser), and the skin (~2 mm^2^) was abraded using a MultiPro Dremel with a grindstone attachment. B16.F10‐OVA‐expressing melanoma cells (1 × 10^5^) were resuspended in 10 μl of Matrigel™ (BD Biosciences) and orthotopically applied to the lesion. Set Matrigel was covered with a piece of Op‐site FlexigridTM (Smith and Nephew). The torsos of the mice were wrapped with a soft hypoallergenic MicroporeTM tape (3M Health Care) and then by a stronger porous polyethylene TransporeTM tape (3M Health Care) to protect the abraded site. Twenty‐four hours later, 2 × 10^5^ FACS‐purified CD8^+^ naive CD44^lo^CD62L^hi^ OT‐1 T cells were adoptively transferred. Recipient mice were monitored daily until bandages were removed 6 days after tumour engraftment.

### Treatment of AT‐3‐OVA tumour‐bearing Ly5.1 mice

Female Ly5.1 mice were anaesthetised with Ketamine (100 mg/kg) and Xylazil (10 mg/kg) (Troy Laboratories) and were injected orthotopically with 1 × 10^6^ AT‐3‐OVA cells resuspended in 20 μl D‐PBS into the fourth mammary fat pad. At day 7 post‐tumour injection, 2 × 10^6^ FACS‐purified CD8^+^CD44^hi^CD62L^hi^ OT‐1 T cells were adoptively transferred.

### Analysis of tumour‐infiltrating T cells

Tumour‐bearing mice were sacrificed, and tumours were excised and digested at 37°C for 30 min using a cocktail of 1 mg/ml collagenase type IV (Worthington Biochemicals) and 0.02 mg/ml DNase (Sigma‐Aldrich) in DMEM supplemented with 2% (v/v) FBS. Cells were passed through a 70‐μm cell strainer (BD Biosciences) twice and processed for flow cytometry. For the detection of intracellular cytokines in tumour‐infiltrating lymphocytes, cells (1 × 10^6^/200 μl) were stimulated with PMA (20 ng/ml; Sigma‐Aldrich) and ionomycin (1 μg/ml; Sigma‐Aldrich) in the presence of GolgiPlug™ and GolgiStop™ (BD Biosciences) for 4 h at 37°C in complete T‐cell medium.

### Re‐challenge of tumour‐infiltrating T cells

AT‐3‐OVA tumour‐bearing C57BL/6 mice were sacrificed, and tumours were collected in ice‐cold D‐PBS and then digested with 50 μg/ml Liberase (Roche Diagnostics, Indianapolis, IN) in Hank's Balanced Salt Solution (Ca^2+^, Mg^2+^) and 10 mM HEPES for 1 h at 37°C. Cells were passed through a 70‐μm cell strainer twice (BD Biosciences, San Jose, CA) and washed three times with 50 ml PBS. Cells were resuspended in DMEM supplemented with 10% FBS, 2 mM l‐glutamine, penicillin (100 units/ml), streptomycin (100 μg/ml) and seeded into 24‐well plates. Cells were incubated at 37°C until they reached 80% confluency. Tumour‐infiltrating T cells from AT‐3‐OVA tumour‐bearing C57BL/6 mice *Ptpn2* ^*fl*/*fl*^ and *Lck*‐Cre;*Ptpn2* ^*fl*/*fl*^ mice were labelled with 2 μM CTV and incubated with adherent, 80% confluent AT‐3‐OVA tumour cells overnight. GolgiPlug™ and GolgiStop™ were added 3 h before cells were processed for intracellular staining for IFNγ and TNF by flow cytometry.

### Cytotoxic T‐cell assays

For the assessment of CAR T‐cell cytotoxicity, HER‐2‐expressing 24JK sarcoma cells (5 μM CTV) and HER‐2‐negative 24JK sarcoma cells (0.5 μM CTV) were labelled with CellTrace™ Violet (CTV; Invitrogen‐Molecular Probes) in D‐PBS supplemented with 0.1% (v/v) BSA for 15 min at 37°C. Tumour cells were then washed three times with D‐PBS supplemented with 10% (v/v) FBS and mixed at a 1:1 ratio. FACS‐purified CD8^+^ central memory (CD44^hi^CD62L^hi^) or effector/memory (CD44^hi^CD62L^lo^) or total HER‐2‐specific CAR T cells were added at different concentrations to the mix of HER‐2‐expressing (5 × 10^4^) and HER‐2‐negative (5 × 10^4^) 24JK sarcoma cells and incubated for 4 h at 37°C in complete T‐cell medium. Antigen‐specific target cell lysis (24JK‐HER‐2 cell depletion) was monitored by flow cytometry.

### Immunohistochemistry

For brain immunohistochemistry, mice were perfused transcardially with heparinised saline [10,000 units/l heparin in 0.9% (w/v) NaCl] followed by 4% (w/v) paraformaldehyde in phosphate buffer (0.1 M, pH 7.4). Brains were post‐fixed overnight and then kept for 4 days in 30% (w/v) sucrose in 0.1 M phosphate buffer to cryoprotect the tissue, before freezing on dry ice. 30‐μm sections (120 μm apart) were cut in the coronal plane throughout the entire rostral‐caudal extent of the cerebellum. For detection of CD3ε, sections were subjected to antigen retrieval in citrate acid buffer [10 mM sodium citrate, 0.05% (v/v) Tween 20, pH 6.0] at 85°C for 20 min. Sections were incubated at room temperature for 2 h in blocking buffer [0.1M phosphate buffer, 0.2% (v/v) Triton X‐100, 10% (v/v) normal goat serum (Sigma‐Aldrich) and then overnight at 4°C in rabbit anti‐CD3 (Dako) in 1% (v/v) blocking buffer. After washing with PBS, sections were incubated with goat anti‐rabbit Alexa Fluor 488‐conjugated secondary antibody (Life Technologies) in blocking buffer for 2 h at room temperature. Sections were mounted with Mowiol 4‐88 mounting media and visualised using an Olympus Provis AX70 microscope. Images were captured with an Olympus DP70 digital camera and processed using AnalySIS (Olympus) software. To assess gross tissue morphology, a subset of sections throughout the entire rostral‐caudal extent of the cerebellum were stained with haematoxylin and eosin.

For detection of HER‐2, 30‐μm sections throughout the entire rostral‐caudal extent of the cerebellum from C57BL/6 or HER‐2 TG C57BL/6 mice were subjected to antigen retrieval and incubated in blocking buffer (as described above). Sections were then incubated overnight in (4°C) with anti‐c‐ErbB2/c‐Neu (Ab‐3) mouse mAb (3B5) (1:500, Calbiochem). After washing with PBS, HER‐2‐positive cells were visualised using rabbit IgG VECTORSTAIN ABC Elite and DAB (3,3′‐diaminobenzidine) Peroxidase Substrate Kits (Vector Laboratories, UK) and visualised using a bright field microscope.

For mammary fat pad tumour immunohistochemistry, animals were culled and mammary fat pad immediately dissected and fixed in buffered formalin solution for 48 h. Tissues were embedded in paraffin and 4‐μm sections of the entire block prepared. Every tenth to fourteenth section of the tissue was used to detect HER‐2 and CD3 by immunohistochemistry. After deparaffinisation and rehydration, sections were subjected to antigen retrieval in Tris/EDTA buffer (pH 8.0) at 120°C for 10 min. Sections were blocked with 5% (v/v) bovine serum albumin in 0.1 M phosphate buffer for 1 h at room temperature and incubated overnight (4°C) with anti‐c‐ErbB2/c‐Neu (Ab‐3) mouse mAb (3B5) (1:500, Calbiochem) or anti‐CD3 (1:500; RAM 34 clone, 14‐0341, Affymetrix eBioscience, San Diego, CA). After washing with PBS, HER‐2‐ and CD3‐positive cells were visualised using rabbit IgG VECTORSTAIN ABC Elite and DAB (3,3′‐diaminobenzidine) Peroxidase Substrate Kits (Vector Laboratories, UK) and counterstained with haematoxylin. Sections were visualised on a Zeiss Axioskop 2 mot plus microscope (Carl Zeiss, Göttingen, Germany).

### Quantitative real‐time PCR

RNA was extracted with TRIzol reagent (Thermo Fisher Scientific, #15596018) and RNA quality and quantity determined using a NanoDrop 2000 (Thermo Fisher Scientific). mRNA was reverse transcribed using a High‐Capacity cDNA Reverse Transcription Kit (Applied Biosystems, #4368814) and processed for quantitative real‐time PCR either using the Fast SYBR™ Green Master Mix (Applied Biosystems, #4385612). Primer sets from PrimePCR^™^ SYBR^®^ Green Assay (Bio‐Rad, #10025636) were utilised to perform quantitative PCR detecting *Cxcl9, Cxcl10, Tgfb1*,* Cd274*,* H2‐K1* and *Rps18*. Primer sets for *HER‐2, Nono and SerpinB14 (ovalbumin)* were purchased from Sigma‐Aldrich. Relative gene expression (ΔCt) was determined by normalisation to the house‐keeping gene *Nono* throughout the study except for Fig 2C, where *Rps18* was used and ΔΔCt analysis performed.

**Table:** 

*Cxcl9*	Bio‐Rad PrimePCR^™^ SYBR^®^ Green Assay: qMmuCID0023784
*Cxcl10*	Bio‐Rad PrimePCR^™^ SYBR^®^ Green Assay: qMmuCED0049500
*Cd274*	Bio‐Rad PrimePCR^™^ SYBR^®^ Green Assay: qMmuCED0044192
*H2‐K1*	Bio‐Rad PrimePCR^™^ SYBR^®^ Green Assay: qMmuCED0004490
*Tgfb1*	Bio‐Rad PrimePCR^™^ SYBR^®^ Green Assay: qMmuCED0044726
*HER‐2*	*Forward: 5′‐GCTCTTTGAGGACAACTATG‐3′* *Reverse: 5′‐TCAAGATCTCTGTGAGGC‐3′*
*SerpinB14 (ovalbumin)*	*Forward: 5′‐GGCATCAATGGCTTCTGAGAA‐3′* *Reverse: 5′‐CCAACATGCTCATTGTCCCA‐3′*
*Nono*	*Forward: 5′‐GCCAGAATGAAGGCTTGACTAT‐3′* *Reverse: 5′‐TATCAGGGGGAAGATTGCCCA‐3′*
*Rps18*	Bio‐Rad PrimePCR^™^ SYBR^®^ Green Assay: qMmuCED0045430

### Ptpn2 knockdown using siSTABLE™ siRNAs targeting in murine CAR T cells

*Ptpn2* was knocked down transiently in HER‐2 CAR T cells using nuclease resistant *Ptpn2* siRNA duplexes (*Ptpn2*; GCCCAUAUGAU CACAGUCG) (siSTABLE™; Dharmacon Thermo Scientific); siRNA duplexes (siSTABLE™) for enhanced green fluorescent protein (GFP; CAAGCUGACCCUGAAGUUC) were used as a control. CAR T cells were transfected with *Ptpn2* siRNA (300 nM) conjugated to FITC or GFP siRNA (300 nM) conjugated to FITC 2 days prior to adoptive T‐cell therapy using the Mouse T‐cell Nucleofector™ Kit (Lonza Bioscience) according to the manufacturer's instructions.

### CRISPR‐Cas9 genome editing in murine CAR T cells

*Ptpn2* was deleted in HER‐2 CAR T cells using Cas9 ribonucleoprotein (RNP)‐mediated gene editing. Briefly, total CAR T cells were transfected with recombinant Cas9 (74 pmol; Alt‐R S.p. Cas9 Nuclease V3, IDT) pre‐complexed with short guide (sg) RNAs (600 pmol; Synthego) targeting the *Ptpn2* locus (*Ptpn2*; 5′‐AAGAAGUUACAU CUUAACAC) or non‐targeting sgRNAs (GCACUACCAG AGCUAA CUCA) as a control 2 days prior to adoptive T‐cell therapy using the P3 Primary Cell 4D‐Nucleofector X™ Kit (Lonza Bioscience) according to the manufacturer's instructions.

### Overexpression of Ptpn2 in HER‐2‐E0771 cells

The Tet‐on 3G inducible expression system (Clontech Laboratories) was used to generate the HER‐2‐E0771‐PTPN2^hi^ cell line. Briefly, murine *Ptpn2* cDNA from HER‐2‐E0771 cells was reverse transcribed and amplified by PCR and cloned into the *Sma*I and *Eco*R1 restriction sites of Tre‐3G plasmid (Clontech Laboratories) to generate the Tre‐3G‐*Ptpn2* construct; the fidelity of the cloned cDNA was confirmed by dideoxy sequencing. HEK293T cells were transfected with either the Tre‐3G‐*Ptpn2 or* Tet‐On 3G constructs using the Lenti‐X Packing system (Clontech Laboratories) according to the manufacturer's instructions. HER‐2‐E0771 mammary tumour cells were transduced first with Tet‐On 3G lentivirus and selected with G418 (0.8 mg/ml) and subsequently transduced with Tre‐3G‐*Ptpn2* lentivirus and selected with puromycin (1.5 μg/ml). Where indicated, the resultant HER‐2‐E0771‐PTPN2^hi^ cells were incubated with 2 mg/ml doxycycline (DOX) to induce the expression of PTPN2. Cells were serum starved in DMEM medium without FBS for 12 h and stimulated with 2 ng/ml IFNγ for the indicated times and processed for immunoblotting or incubated with 1 ng/ml IFNγ for 24 h and processed for quantitative real‐time PCR.

For *in vivo* studies, 2 × 10^5^ HER‐2‐E0771‐PTPN2^hi^ cells were resuspended in 20 μl D‐PBS and injected orthotopically into the fourth mammary fat pad of female human HER‐2 TG mice. At day 5 post‐tumour cell injection, mice were administered DOX (2 mg/ml) in the drinking water for the entirety of the experiment. At day 6 post‐tumour cell injection, human HER‐2 TG mice were pre‐conditioned with total body irradiation (4 Gy) prior to the adoptive transfer of 6 × 10^6^ FACS‐purified CD8^+^CD44^hi^CD62L^hi^ CAR T cells. Mice were treated with 50,000 IU IL‐2 on days 0–4 after T‐cell transfer.

### Statistical analyses

Statistical analyses were performed with GraphPad Prism software 7.0b using the non‐parametric using 2‐tailed Mann–Whitney *U*‐test, the parametric 2‐tailed Student's *t*‐test, the 1‐way or 2‐way ANOVA test using Turkey or Sidak post hoc comparison or the log‐rank (Mantel–Cox test) where indicated. *P < 0.05, **P < 0.01, ***P < 0.001 and ****P < 0.0001 were considered as significant.

### Animal ethics

All experiments were performed in accordance with the NHMRC Australian Code of Practice for the Care and Use of Animals. All protocols were approved by the Monash University School of Biomedical Sciences Animal Ethics Committee (Ethics number: MARP/2012/124) or the Peter MacCallum Animal Ethics and Experimentation Committee (Ethics numbers: E570, E582 and E604).

## Author contributions

Conceptualisation: TT; Methodology: FW, K‐HL, XD, SL, KH, GTD, PKG, CK, DM, PAB, MAH, SLP, JW, SZ, Z‐YZ, JO, TG, PKD and TT; Data acquisition: FW, K‐HL, XD, SL, KH, GTD, PKG, MAH and SLP; Data analysis: FW, K‐HL, XD, SL, KH, GTD, PKG; MAH; TG, PKD and TT; Data interpretation: FW, K‐HL, XD, SL, KH, GTD, PKG, CK, DM, PAB, MAH, SLP, JW, SZ, Z‐YZ, JO, TG, PKD and TT; Writing‐Original Draft: TT; Writing‐Review and Editing: FW, K‐HL, XD, SL, KH, GTD, PKG, CK, DM, PAB, MAH, SLP, JW, SZ, Z‐YZ, JO, TG, PKD and TT. Funding acquisition: FW, PKD, TG and TT; Final approval for publishing: FW, K‐HL, XD, SL, KH, GTD, PKG, CK, DM, PAB, MAH, SLP, JW, SZ, Z‐YZ, JO, TG, PKD and TT.

## Conflict of interest

The authors declare that they have no conflict of interest.

## Supporting information



Appendix

Expanded View Figures PDF

Source Data for Appendix

Review Process File
