# Supplementary material for: PTPN2 phosphatase deletion in T cells promotes anti‐tumour immunity and CAR T‐cell efficacy in solid tumours
Source: EMBO J. 2019 Dec 5;39(2):e103637. doi: 10.15252/embj.2019103637 (PMC6960448; doi:10.15252/embj.2019103637)
Supplement: Supplementary file 1 — Appendix [file EMBJ-39-e103637-s001.pdf]

APPENDIX TABLE OF CONTENTS

Figure S1: ***Tissue architecture and tumours in p53$^{+/-}$ mice.***

Figure S2: ***Inflammatory disease in Ptpn2$^{fl/fl}$;p53$^{+/-}$ and Lck*-Cre;*Ptpn2$^{fl/fl}$;p53$^{+/-}$ mice.***

Figure S3: ***PTPN2 deficiency increases T cell infiltration and activation and decreases T cell exhaustion in a syngeneic model of triple negative breast cancer.***

Figure S4: ***PTPN2 deficiency enhances the antigen-induced cytotoxic activity of CAR T cells.***

Figure S5: ***PTPN2 deficiency in CAR T cells does not result in systemic inflammation and collateral tissue damage.***

Figure S6: ***PTPN2 deficiency does not result in marked CAR T cell cerebellar infiltration and tissue damage.***

Table EV1: ***Pathology in Ptpn2$^{fl/fl}$;p53$^{+/-}$mice.***

APPENDIX FIGURE LEGENDS

Appendix figure S1. Tissue architecture and tumours in p53$^{+/-}$ mice. Gross images and histological analyses (Hematoxylin and Eosin: H&E) from 12 month old *Ptpn2$^{fl/fl}$;p53$^{+/-}$* and *Lck*-Cre;*Ptpn2$^{fl/f}$;p53$^{+/-}$* mice. Scale bars H&E: Thymus/Thymoma/ Lymph node: 500 µm (full size), 200 µm and 50 µm (zoom). Spleen: 800 µm (full size), 200 µm and 50 µm (zoom). Sarcoma: 2 mm (full size), 300 µm and 50 µm (zoom). Scale bars organ images: 10 mm.

Appendix figure S2. Inflammatory disease in Ptpn2$^{fl/fl}$;p53$^{+/-}$ and Lck-**Cre;*****Ptpn2$^{fl/fl}$;p53$^{+/-}$ mice.*** Inflammatory serum cytokines in **a**) *Ptpn2$^{fl/fl}$;p53$^{+/-}$* versus *Lck*-Cre;*Ptpn2$^{fl/fl}$;p53$^{+/-}$* mice and **b**) *Ptpn2$^{fl/fl}$* versus *Lck*-Cre;*Ptpn2$^{fl/fl}$* mice were determined with the LEGENDplex T_H Cytokine Panel™ kit. **c**) Livers from 12 month old *Ptpn2$^{fl/fl}$;p53$^{+/-}$* versus *Lck*-Cre;*Ptpn2$^{fl/fl}$;p53$^{+/-}$* mice were fixed in formalin and processed for histology. Gross images are shown of *Ptpn2$^{fl/fl}$;p53$^{+/-}$* livers bearing hepatomas (T). Scale bars H&E/Picrosirius Red: 3 mm (full size), 300 µm and 100 µm (zoom). Scale bars organ images: 5 mm. **d**) Day 26 (d26) tumour infiltrating lymphocytes (TILs) from *Ptpn2$^{fl/fl}$* verus *Lck*-Cre;*Ptpn2$^{fl/fl}$* mice were assessed for the proportion of $CD4^+CD25^{hi}FoxP3^+$ T_{regs} by flow cytometry. **e**) Day 26 (d26) tumour infiltrating lymphocytes (TILs) from *Ptpn2$^{fl/fl}$* versus *Lck*-Cre;*Ptpn2$^{fl/fl}$* mice were incubated overnight with α-CD3 (10 µg/ml) and IL-2 (50 ng/ml) and intracellular Nur77 and IL-10 in $CD4^+CD25^{hi}FoxP3^+$ T_{regs} were determined by flow cytometry. Significance in (b, d, e) was determined using 2-tailed Mann-Whitney U Test. *$p<0.05$, **$p<0.01$, ***$p<0.001$.

Appendix figure S3. PTPN2 deficiency increases T cell infiltration and activation and decreases T cell exhaustion in a syngeneic model of triple negative breast cancer. AT-3-OVA mammary tumour cells (1x10^6) were injected into the fourth inguinal fat pads of female

Ly5.1$^+$ mice. Seven days after tumour injection FACS-purified naïve CD8$^+$CD44^{lo}CD62L^{hi} lymph node T cells from Ly5.2$^+$ OT-1;*Ptpn2$^{fl/fl}$* (2x10^6) versus Ly5.2$^+$ OT-1;*Lck*-Cre;*Ptpn2$^{fl/fl}$* (2x10^6) mice were adoptively transferred into tumour-bearing Ly5.1$^+$ mice. (**a**) 21 days after adoptive transfer Ly5.1$^-$Ly5.2$^+$ donor T cell numbers were determined in the draining lymph nodes (dLN) by flow cytometry. **b)** 9 days after adoptive transfer Ly5.1$^-$Ly5.2$^+$ donor T cell numbers were determined in the AT-3-OVA tumours by flow cytometry. **c**) 9 days after adoptive transfer Ly5.1$^-$Ly5.2$^+$ donor T cells isolated from mammary tumours or dLN were assessed for the proportion of Ly5.2$^+$IFNγ$^+$TNF$^+$ and Ly5.2$^+$GrzB$^+$ T cells. **d-g**) 9 days or 21 days post adoptive transfer Ly5.1$^-$Ly5.2$^+$ donor T cells isolated from mammary tumours or dLN were assessed for **d, e, g**) PD-1 and LAG-3 MFIs or **f**) CD44 MFIs. **h**) AT-3-OVA tumour growth was monitored in female Ly5.1$^+$ mice up to 42 days post adoptive transfer. Representative flow cytometry profiles and results (means ± SEM) from at least two independent experiments are shown. Significance in (a-c, e-g) was determined using 2-tailed Mann-Whitney U Test. In (h) significance was determined using 2-way ANOVA Test. *p<0.05, **p<0.01, ***p<0.001, ****p<0.0001.

Appendix figure S4. PTPN2 deficiency enhances the antigen-induced cytotoxic activity of CAR T cells. **a**) *Ptpn2$^{fl/fl}$* versus *Lck*-Cre;*Ptpn2$^{fl/fl}$* HER-2 CAR T cells were incubated with 24JK-HER-2 or 24JK sarcoma cells and the proportion of CD8$^+$IFNγ$^+$ CAR T cells was determined by flow cytometry. **b**) FACS-purified CD8$^+$ central memory (CD44^{hi}CD62L^{hi}) and effector/memory (CD44^{hi}CD62L^{lo}) *Ptpn2$^{fl/fl}$* versus *Lck*-Cre;*Ptpn2$^{fl/fl}$* HER-2 CAR T cells were incubated with 5 μM CTV-labelled (CTV^{bright}) 24JK-HER-2 and 0.5 μM CTV-labelled (CTV^{dim}) 24JK sarcoma cells. Antigen-specific target cell lysis (24JK-HER-2 versus 24JK response) was monitored for the depletion of CTV^{bright} 24JK-HER-2 cells by flow cytometry. Representative flow cytometry profiles and results (means ± SEM) from at three independent

experiments are shown. Significance in (a) was determined using 2-tailed Mann-Whitney U Test. Significance in (b) was determined using 2-tailed Student's t test. *$p<0.05$, **$p<0.01$, ***$p<0.001$.

Appendix figure S5. PTPN2 deficiency in CAR T cells does not result in systemic inflammation and collateral tissue damage. HER-2-E0771 cells ($2x10^5$) were injected into the fourth inguinal mammary fat pads of female HER-2 TG mice. Six days after tumour injection HER-2 TG mice received total body irradiation (4 Gy) followed by the adoptive transfer of $6x10^6$ FACS-purified $CD8^+CD44^{hi}CD62L^{hi}$ central memory HER-2 CAR T cells generated from *Ptpn2*$^{fl/fl}$ versus *Lck*-Cre;*Ptpn2*$^{fl/fl}$ splenocytes. Mice were injected with IL-2 (50,000 IU/day) on days 0-4 after adoptive CAR T cell transfer. **a**) Lymphocytes isolated from the spleens and livers of HER-2 TG recipient mice were assessed for $CD3^+CD8^+$ donor CAR T cell numbers and PD-1 mean fluorescence intensities (MFI) by flow cytometry 21 days after adoptive CAR T cell transfer. **b**) Inflammatory serum cytokines in HER-2 TG recipient mice were determined with the LEGENDplex T_H Cytokine Panel™ kit. **c**) The tumour-free contralateral fourth inguinal mammary fat pads were fixed in formalin at 21 days post CAR T cell transfer and processed for histological assessment (hematoxylin and eosin) monitoring for tissue architecture and lymphocytic infiltrates. Scale bars: 100 μm. **d**) HER-2-E0771 breast cancer cells ($2x10^5$) were injected into the fourth inguinal mammary fat pads of female HER-2 TG mice. Six days after tumour injection HER-2 TG mice received total body irradiation (4 Gy) followed by the adoptive transfer of $20x10^6$ FACS-purified $CD8^+CD44^{hi}CD62L^{hi}$ central memory HER-2 CAR T cells generated from *Ptpn2*$^{fl/fl}$ versus *Lck*-Cre;*Ptpn2*$^{fl/fl}$ splenocytes. Mice were injected with IL-2 (50,000 IU/day) on days 0-4 after adoptive CAR T cell transfer and tumour growth was monitored. **e**) Lungs and livers were fixed in formalin at 21 days post CAR T cell transfer and processed for histological assessment (hematoxylin and eosin)

monitoring for tissue architecture and lymphocytic infiltrates. Scale bars: 200 μm. Representative results (means ± SEM) from at least two independent experiments are shown. In (d) significance was determined using 2-way ANOVA Test. ****p<0.0001.

Appendix figure S6. PTPN2 deficiency does not result in marked CAR T cell cerebellar infiltration and tissue damage. **a**) Cerebella from +/+ C57BL/6 and HER-2 TG C57BL/6 mice were processed for α-HER-2 immunohistochemistry. Scale bars: 2 mm. **b**) *HER-2* gene expression in cerebella from HER-2 TG mice, E0771-HER-2 tumours or HER-2-E0771 cells were assessed by quantitative real time PCR. **c**) Cerebella from HER-2 TG C57BL/6 mice administered *Ptpn2*$^{fl/fl}$ or *Lck*-Cre;*Ptpn2*$^{fl/fl}$ mCherry$^+$ HER-2 CAR T cells were processed for α-CD3ε immunohistochemistry 10 days after the transfer of HER-2 CAR T cells. CD3ε$^+$mCherry$^+$ CAR T cells were not detected in the cerebellar lobules, including the 8th cerebellar lobule (8Cb). Some CD3ε$^+$mCherry$^+$ staining was evident adjacent to the crus 1 of the ansiform lobule (Crus1) in mice treated with PTPN2-deficient HER-2 CAR T cells. Representative images for 3 mice per genotype are shown. **d**) Cerebella from HER-2 TG mice administered *Ptpn2*$^{fl/fl}$ or *Lck*-Cre;*Ptpn2*$^{fl/fl}$ HER-2 CAR T cells were processed for histology (hematoxylin and eosin: H&E) 10 days after the adoptive transfer of HER-2 CAR T cells to assess gross tissue architecture. **e**) CD44 MFIs on CD45$^+$CD8$^+$CD3$^+$ mCherry$^+$ CAR T cells and the proportion of IFNγ$^+$TNF$^+$ versus GrzB$^+$ CD45$^+$CD8$^+$CD3$^+$ mCherry$^+$ CAR T cells isolated from HER-2-E0771 tumours 10 days post adoptive CAR T cell transfer were determined by flow cytometry. **f**) HER-2 TG mice administered *Lck*-Cre;*Ptpn2*$^{fl/fl}$ HER-2 CAR T cells were subjected to a rotarod test 50 days post tumour clearance and the latency to fall determined in consecutive trials. **g**) *Cxcl9 and Cxcl10* gene expression in cerebella from C57Bl/6 and HER-2 TG mice and E0771-HER-2 tumours were assessed by quantitative real time PCR. Representative results (means ± SEM) from at least two independent experiments

are shown. In (b, g) significance was determined using 1-way ANOVA Test. In (e) significance was determined using 2-tailed Mann-Whitney U Test. **$p<0.01$, ***$p<0.001$, ****$p<0.0001$.

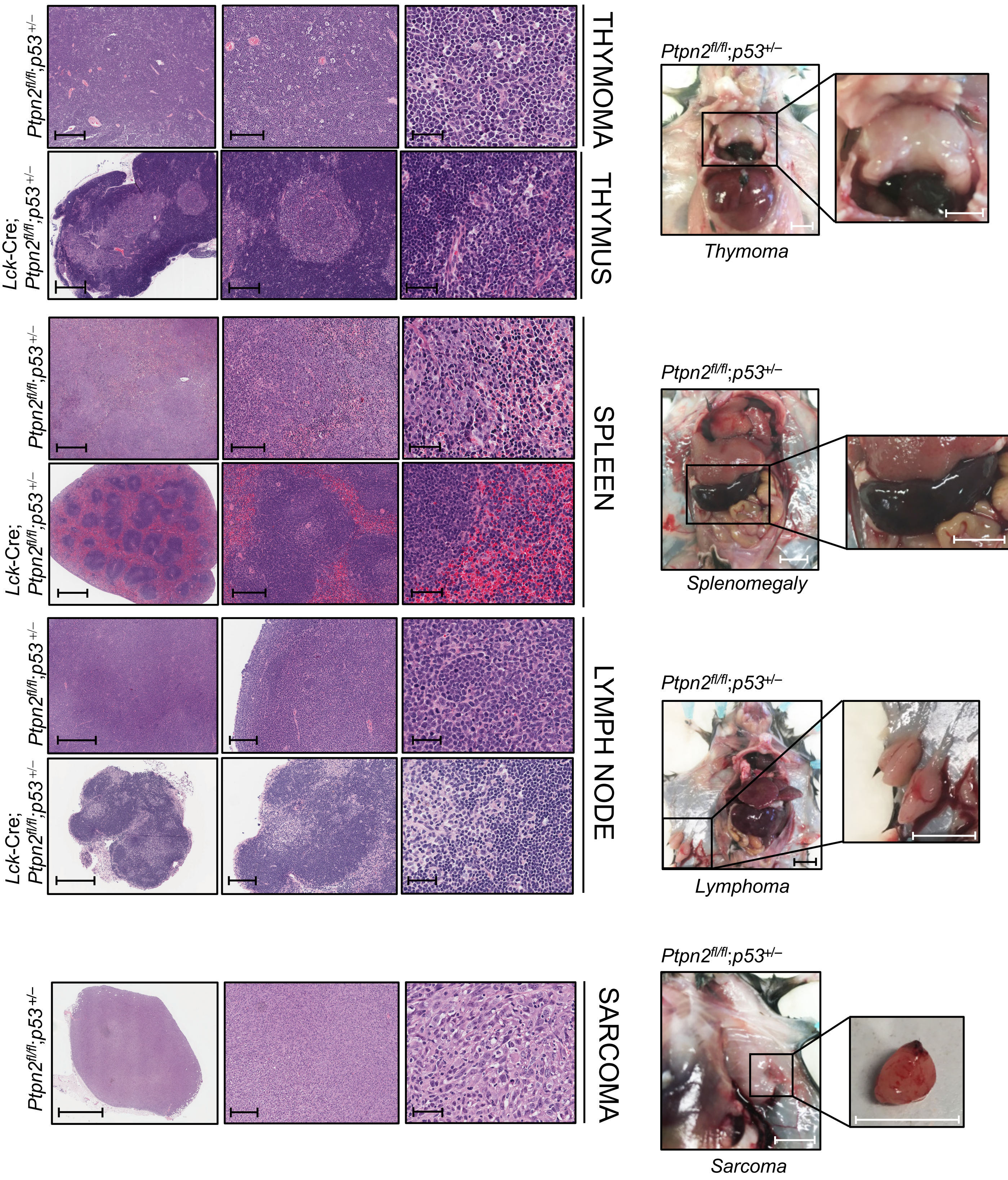

Appendix Figure S1

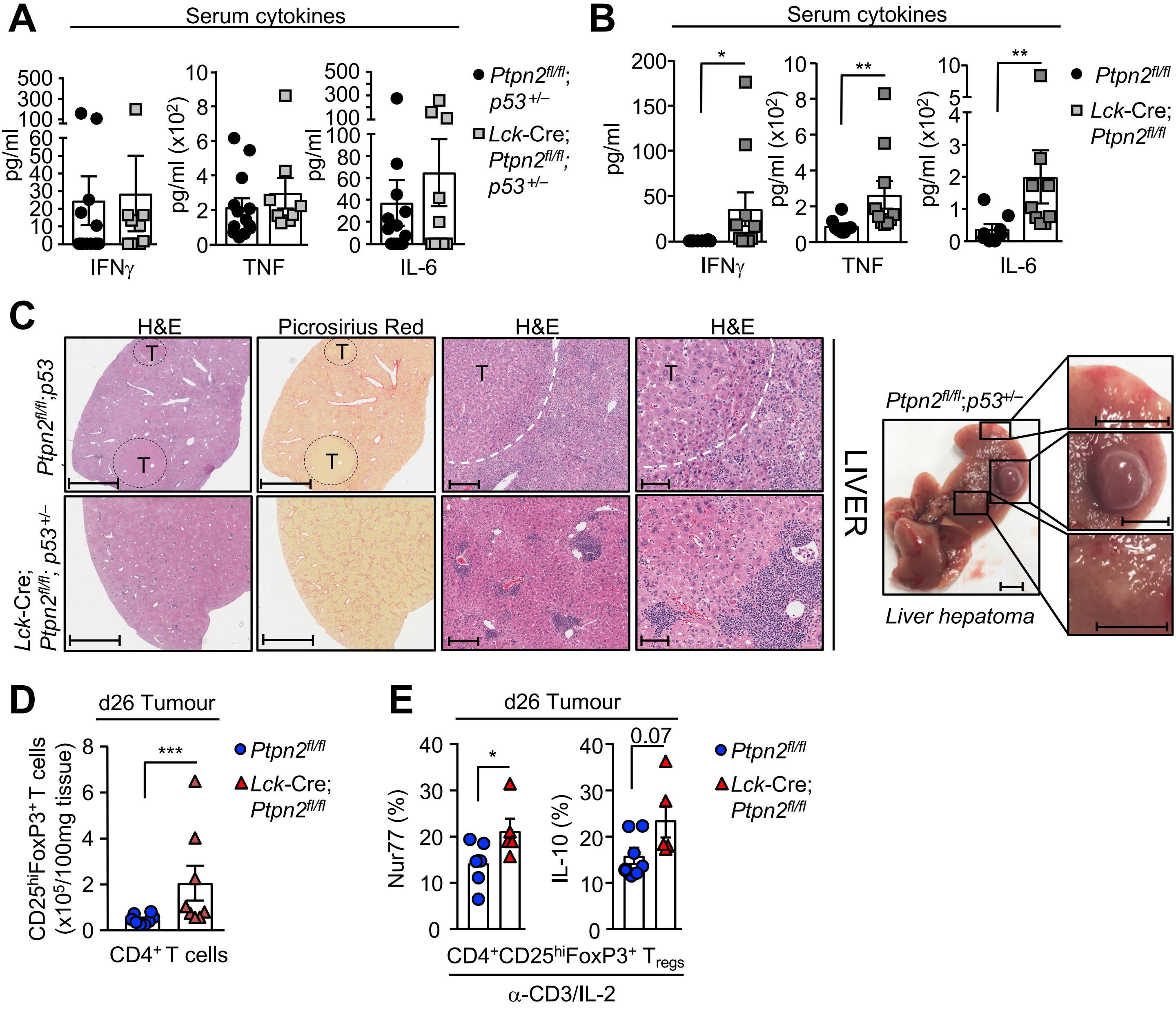

Appendix Figure S2

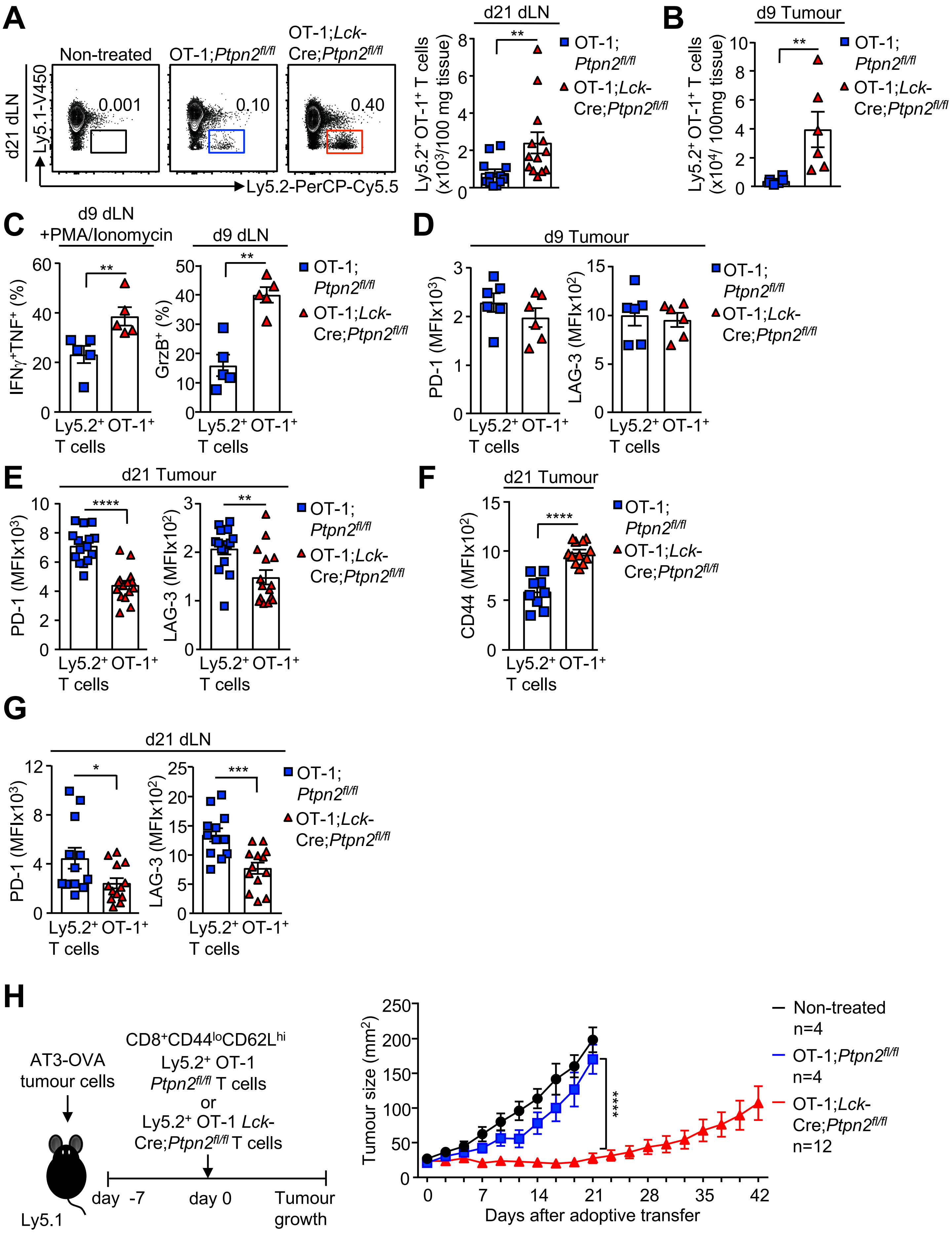

Appendix Figure S3

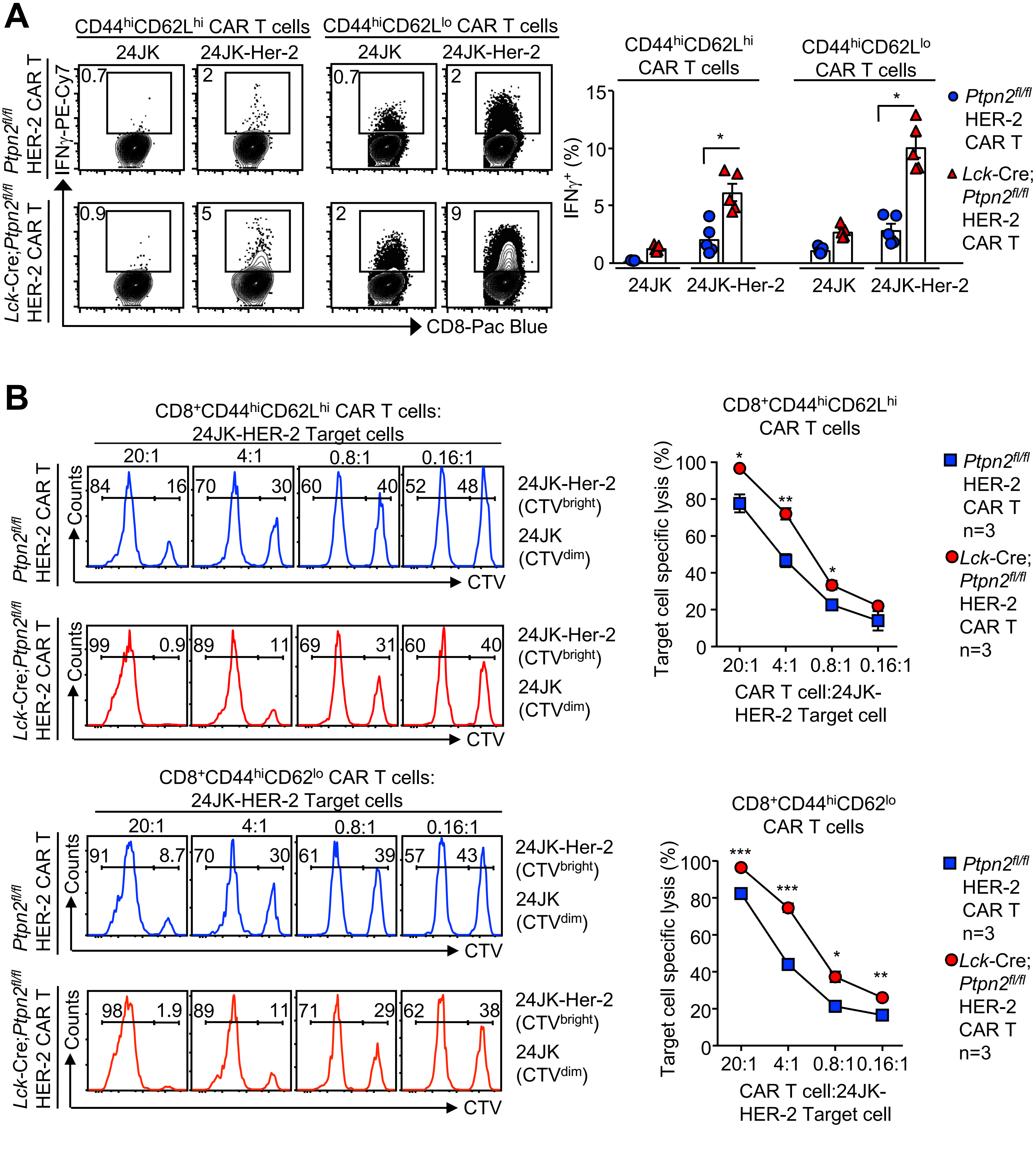

Appendix Figure S4

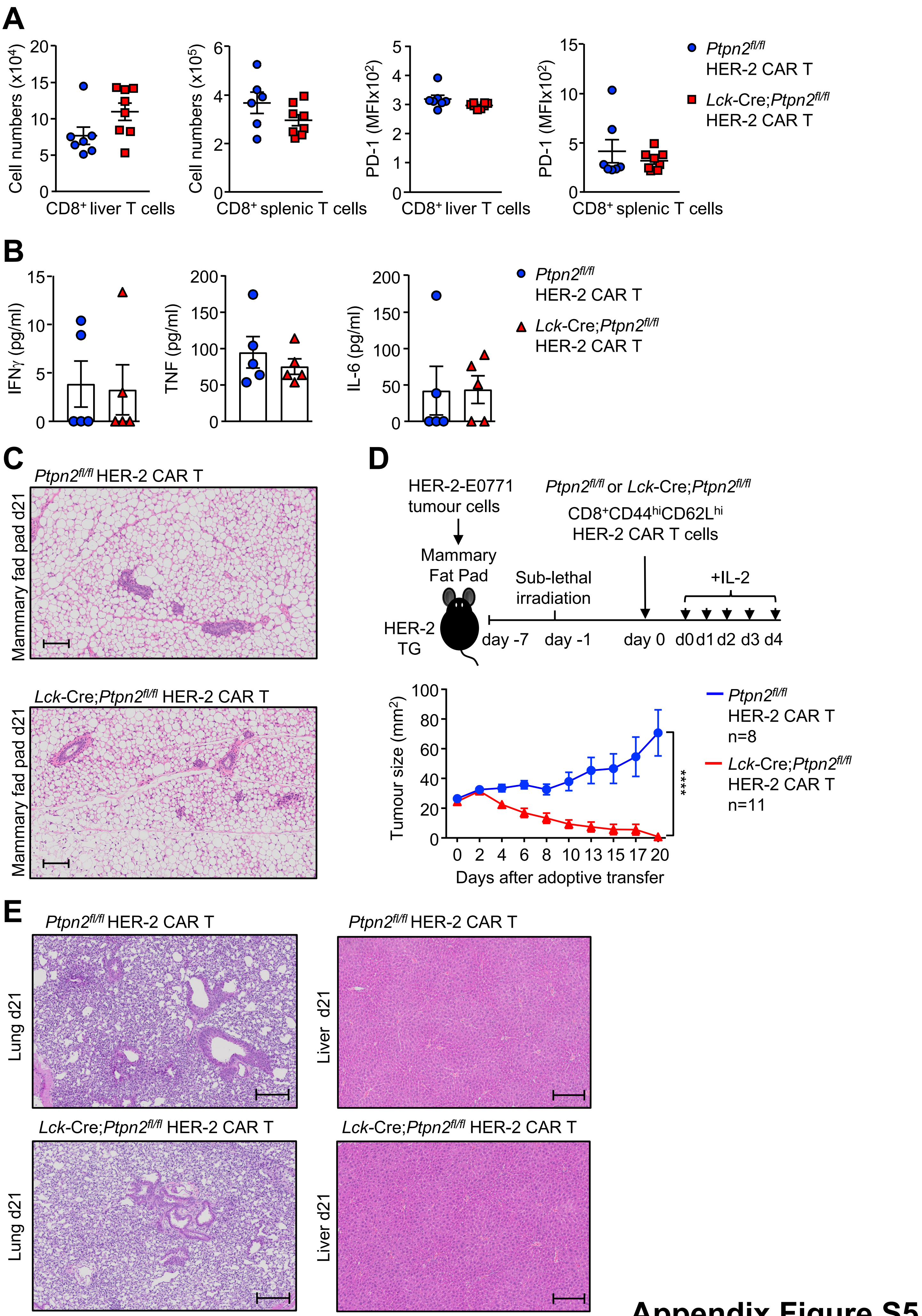

Appendix Figure S5

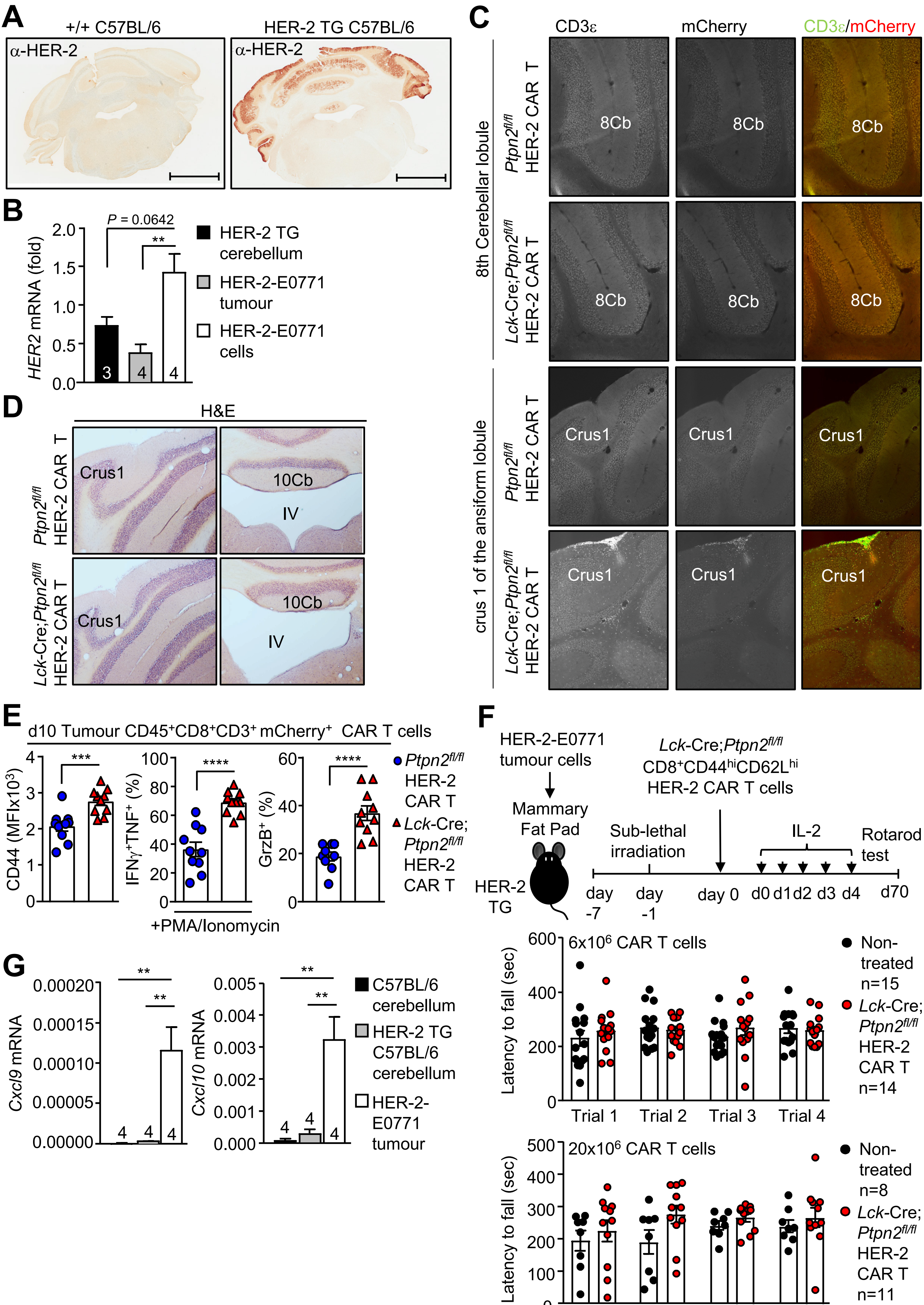

Appendix Figure S6

	Table EV1. Pathology in *Ptpn2*$^{fl/fl}$;*p53*$^{+/-}$ mice							
**Mouse ID**	**Hepatoma**	**Sarcoma/ Carcinoma**	**Thymoma**	**Lymphoma**	**Splenomegaly**	**B1 cell leukemia**	**Thymic DP T cell leukemia**	**Splenic/Hepatic DP T cell leukemia**
**#1**	No	No	**Yes**	No	No	No	**Yes**	**Yes**
**#2**	No	No	No	**Yes**	**Yes**	**Yes**	No	No
**#4**	No	No	No	No	No	**Yes**	No	No
**#6**	No	No	No	No	**Yes**	**Yes**	No	No
**#7**	No	**Yes**	No	No	No	No	**Yes**	**Yes**
**#8**	No	No	No	No	No	No	**Yes**	No
**#14**	**Yes**	No	No	No	**Yes**	No	No	No
**#15**	No	No	No	No	**Yes**	**Yes**	No	No
**#18**	No	**Yes**	No	No	No	No	No	No
**#20**	No	No	**Yes**	No	No	No	**Yes**	**Yes**
**#23**	No	No	**Yes**	No	No	No	**Yes**	**Yes**
**#27**	No	No	No	No	**Yes**	**Yes**	No	No
**#30**	No	**Yes**	No	No	No	No	No	No
**#32**	No	No	No	No	**Yes**	**Yes**	No	No
**#34**	**Yes**	No	No	No	No	No	No	No
