# Supplementary material for: PTPN2 phosphatase deletion in T cells promotes anti‐tumour immunity and CAR T‐cell efficacy in solid tumours
Source: EMBO J. 2019 Dec 5;39(2):e103637. doi: 10.15252/embj.2019103637 (PMC6960448; doi:10.15252/embj.2019103637)
Supplement: Supplementary file 2 — Expanded View Figures PDF [file EMBJ-39-e103637-s002.pdf]

Expanded View Figures

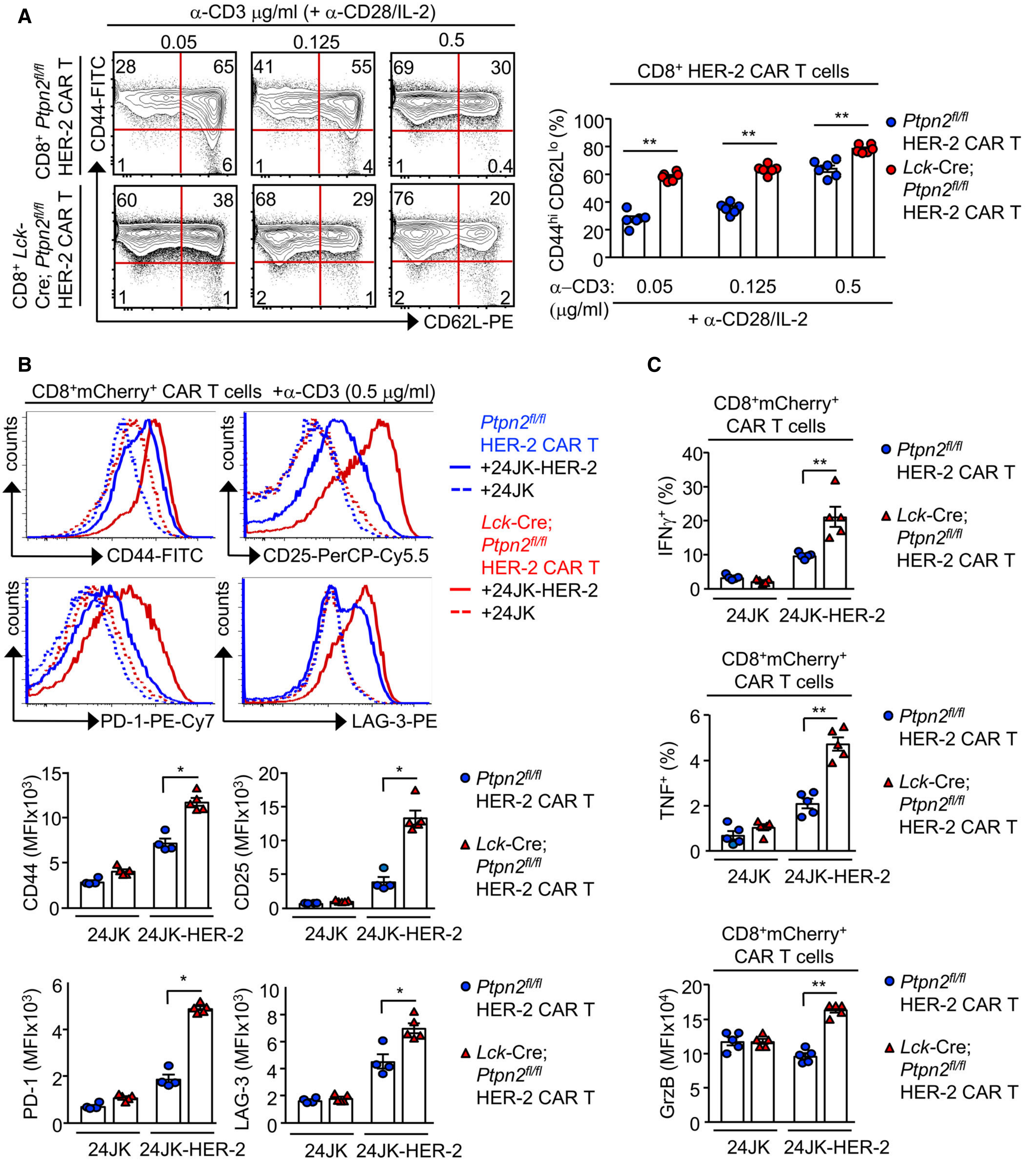

Figure EV1.

Figure EV1. PTPN2 deficiency enhances the generation and the antigen-induced activation of CAR T cells.

A *Ptpn2*$^{fl/fl}$ versus *Lck*-Cre;*Ptpn2*$^{fl/fl}$ HER-2 CAR T cells were generated with varying concentrations of α-CD3 (0.05, 0.125 and 0.5 μg/ml) in the presence of α-CD28 (0.5 μg/ml) and IL-2 (2 ng/ml). After 6 days in culture, the generation of effector/memory (CD44^{hi}CD62L^{lo}) CD8^{+} HER-2 CAR T cells was determined by flow cytometry.

B, C *Ptpn2*$^{fl/fl}$ versus *Lck*-Cre;*Ptpn2*$^{fl/fl}$ HER-2 CAR T cells were incubated with HER-2-expressing 24JK sarcoma cells (24JK-HER-2) and HER-2-negative 24JK sarcoma cells. (B) CD44, CD25, PD-1 and LAG-3 MFIs, (C) the proportion of CD8^{+}IFNγ^{+} versus CD8^{+}TNF^{+} CAR T cells and GrzB mean fluorescence intensity (MFI) were determined by flow cytometry.

Data information: Significance in (A–C) was determined using 2-tailed Mann–Whitney *U*-test. *$P < 0.05$, **$P < 0.01$.

Figure EV2. PTPN2 deficiency increases SFK and IL-2/15-induced STAT5 signalling in CD8^{+} HER-2 CAR T cells.

A *Ptpn2*$^{fl/fl}$ versus *Lck*-Cre;*Ptpn2*$^{fl/fl}$ HER-2 CD8^{+}CD44^{hi}CD62L^{hi} or CD44^{hi}CD62L^{lo} CAR T cells were assessed for intracellular p(Y418)-SFK MFI by flow cytometry.

B HER-2-specific *Ptpn2*$^{fl/fl}$ versus *Lck*-Cre;*Ptpn2*$^{fl/fl}$ CAR T cells were incubated with plate-bound α-CD3 and CD25; CD122 and CD132 MFIs on CD8^{+}CD44^{hi}CD62L^{hi} CAR T cells versus CD8^{+}CD44^{hi}CD62L^{lo} CAR T cells were determined by flow cytometry.

C, D *Ptpn2*$^{fl/fl}$ versus *Lck*-Cre;*Ptpn2*$^{fl/fl}$ HER-2 CAR T cells were incubated with plate-bound α-CD3 and stimulated with recombinant (C) IL-2 and (D) IL-15 for the indicated time points. Intracellular p(Y694)-STAT-5 MFIs on CD8^{+}CD44^{hi}CD62L^{hi} versus CD8^{+}CD44^{hi}CD62L^{lo} CAR T cells were determined by flow cytometry.

E *Ptpn2*$^{fl/fl}$, *Lck*-Cre;*Ptpn2*$^{fl/fl}$ and *Lck*-Cre;*Ptpn2*$^{fl/fl}$;*Lck*$^{+/-}$ HER-2 CAR T cells were incubated with plate-bound α-CD3; CD122 and CD132 MFIs on CD8^{+}CD44^{hi}CD62L^{lo} versus CD8^{+}CD44^{hi}CD62L^{hi} CAR T cells were determined by flow cytometry.

F *Ptpn2*$^{fl/fl}$ versus *Lck*-Cre;*Ptpn2*$^{fl/fl}$ versus *Lck*-Cre;*Ptpn2*$^{fl/fl}$;*Lck*$^{+/-}$ HER-2 CAR T cells were incubated with plate-bound α-CD3 and stimulated with recombinant IL-2 and IL-15 for the indicated time points. Intracellular p(Y694)-STAT-5 MFIs on CD8^{+}CD44^{hi}CD62L^{lo} CAR T cells were determined by flow cytometry.

Data information: Representative results (means ± SEM) are shown from two independent experiments. In (A, B, E), significance was determined using 2-tailed Mann–Whitney *U*-test. **$P < 0.01$.

 © 2019 The Authors

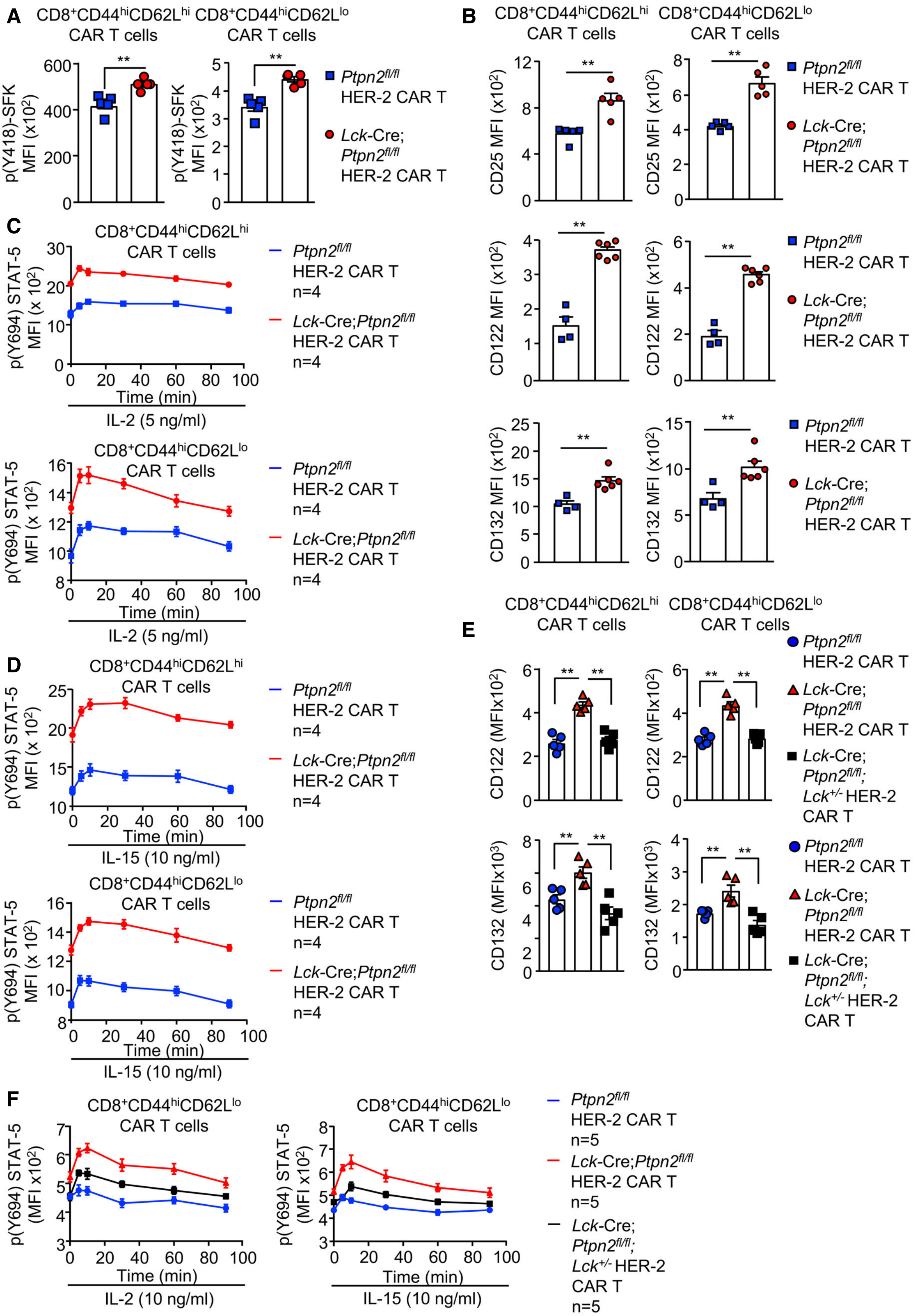
A
CD8+CD44hiCD62Lhi CAR T cells
CD8+CD44hiCD62Llo CAR T cells
p(Y418)-SFK MFI (x10²)
Ptpn2fl/fl HER-2 CAR T
Lck-Cre; Ptpn2fl/fl HER-2 CAR T
B
CD25 MFI (x10²)
CD122 MFI (x10²)
CD132 MFI (x10²)
C
p(Y694) STAT-5 MFI (x 10²)
Time (min)
IL-2 (5 ng/ml)
Ptpn2fl/fl HER-2 CAR T n=4
Lck-Cre;Ptpn2fl/fl HER-2 CAR T n=4
D
IL-15 (10 ng/ml)
E
CD122 (MFIx10²)
CD132 (MFIx10³)
Lck-Cre; Ptpn2fl/fl; Lck+/- HER-2 CAR T
F
p(Y694) STAT-5 (MFI x10²)
IL-2 (10 ng/ml)
IL-15 (10 ng/ml)
Ptpn2fl/fl HER-2 CAR T n=5
Lck-Cre;Ptpn2fl/fl HER-2 CAR T n=5
Lck-Cre; Ptpn2fl/fl; Lck+/- HER-2 CAR T n=5

Figure EV2.

Figure EV3. PTPN2 deficiency enhances CAR T-cell proliferation and tumour infiltration.

A CD8^{+}CD44^{hi}CD62L^{hi} HER-2 CAR T cells generated from *Ptpn2*$^{fl/fl}$ versus *Lck*-Cre;*Ptpn2*$^{fl/fl}$ splenocytes were stimulated with plate-bound α-CD3 and subsequently labelled with CTV and incubated with 24JK-HER-2 cells, and proliferation was determined by flow cytometry.

B HER-2-E0771 tumours isolated from HER-2 TG mice on day 10 after adoptive *Ptpn2*$^{fl/fl}$ versus *Lck*-Cre;*Ptpn2*$^{fl/fl}$ HER-2 CAR T-cell transfer were analysed for CD3^{+} T-cell infiltrates by immunohistochemistry. Scale bars: 500 μm (full size) and 200 μm (zoom).

C HER-2-E0771 cells (2×10^{5}) were injected into the fourth inguinal mammary fat pads of female HER-2 transgenic (TG) mice. Six days after tumour injection, HER-2 TG mice received total body irradiation (4 Gy) followed by the adoptive transfer of 6×10^{6} FACS-purified CD8^{+}CD62L^{hi}CD44^{hi} central memory HER-2 CAR T cells generated from *Ptpn2*$^{fl/fl}$ versus *Lck*-Cre;*Ptpn2*$^{fl/fl}$ splenocytes. Mice were injected with IL-2 (50,000 IU/day) on days 0–4 after adoptive CAR T-cell transfer. Lymphocytes were isolated from the tumours at day 16 post-adoptive transfer and CXCR3, CXCR5 and CCR7 MFIs on CD45^{+}CD8^{+} T cells determined by flow cytometry.

D, E Intracellular T-bet MFIs in CD8^{+} HER-2 CAR T cells were determined by flow cytometry.

Data information: Representative flow cytometry profiles and results (means ± SEM) are shown from two independent experiments. In (A, C, D, E), significance was determined using 2-tailed Mann–Whitney *U*-test. *$P < 0.05$, **$P < 0.01$.

© 2019 The Authors

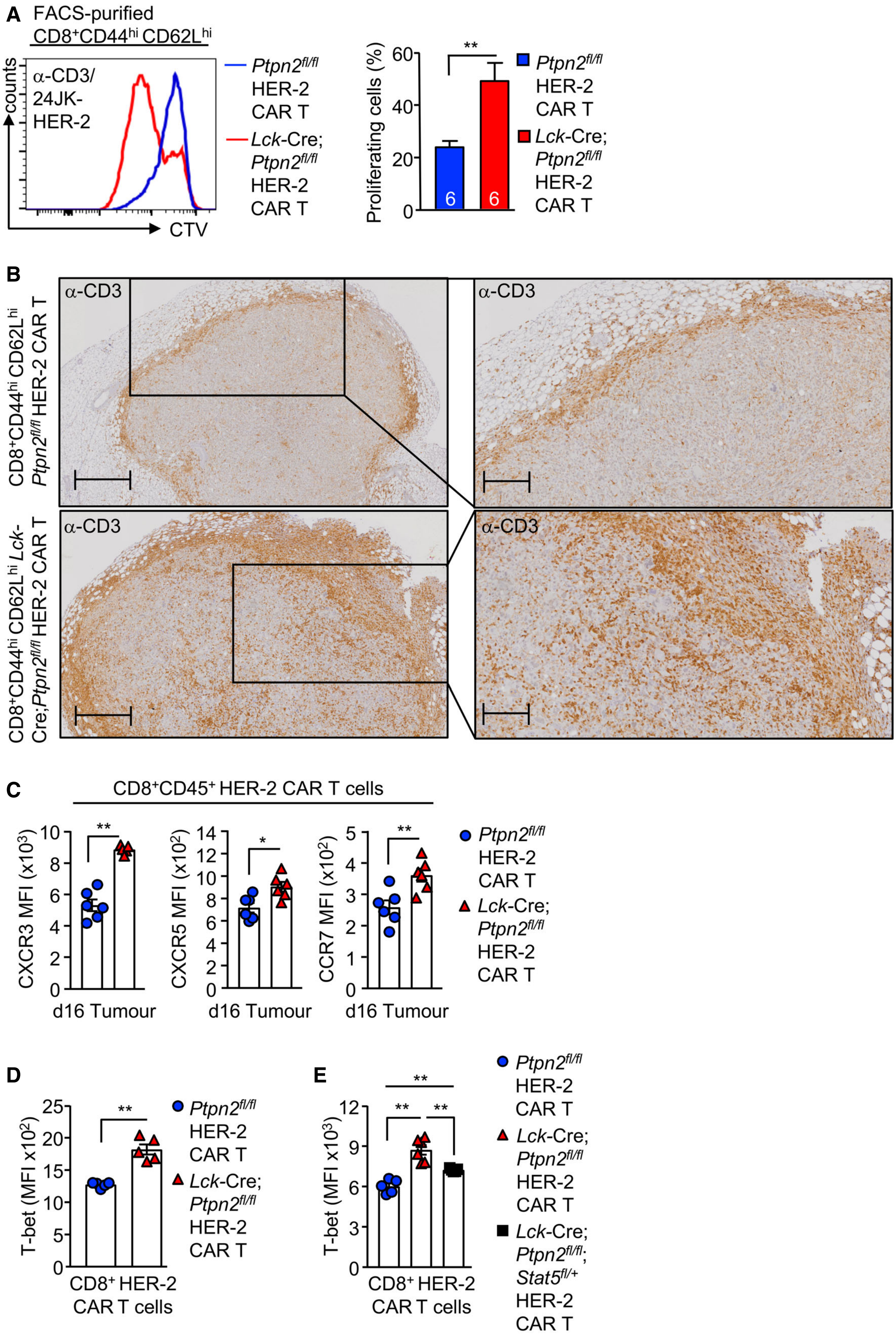
A FACS-purified CD8+CD44hi CD62Lhi
α-CD3/ 24JK-HER-2
counts
CTV
Ptpn2fl/fl HER-2 CAR T
Lck-Cre; Ptpn2fl/fl HER-2 CAR T
Proliferating cells (%)
**
6
6
B
α-CD3
CD8+CD44hi CD62Lhi Ptpn2fl/fl HER-2 CAR T
CD8+CD44hi CD62Lhi Lck-Cre;Ptpn2fl/fl HER-2 CAR T
C CD8+CD45+ HER-2 CAR T cells
CXCR3 MFI (x10³)
CXCR5 MFI (x10²)
CCR7 MFI (x10²)
d16 Tumour
D
T-bet (MFI x10²)
CD8+ HER-2 CAR T cells
E
T-bet (MFI x10³)
Lck-Cre; Ptpn2fl/fl; Stat5fl/+ HER-2 CAR T

Figure EV3.

Figure EV4. PTPN2 deficiency in CAR T cells does not result in autoimmunity.
HER-2-E0771 mammary tumour cells (2×10^5) were injected into the fourth inguinal mammary fat pads of female HER-2 TG mice. Six days after tumour injection, HER-2 TG mice received total body irradiation (4 Gy) followed by the adoptive transfer of 20×10^6 FACS-purified $CD8^+CD44^{hi}CD62L^{hi}$ central memory HER-2 CAR T cells generated from *Ptpn2*$^{fl/fl}$ versus *Lck*-Cre;*Ptpn2*$^{fl/fl}$ splenocytes. Mice were injected with IL-2 (50,000 IU/day) on days 0–4 after adoptive CAR T-cell transfer.

A Serum cytokines were determined with the BD CBA Mouse Inflammation Kit™.
B, C Body core temperatures were measured using a mouse rectal probe, and (C) body weights were monitored.
D Serum anti-nuclear antibodies (ANA) were measured using a mouse anti-nuclear antibodies Ig's (total IgA+G+M) ELISA Kit.
E Serum AST and ALT activities were determined using a Transaminase II Kit.
F, G Lamina propria $CD45^+CD8^+$ donor CAR T-cell numbers (F) and colon lengths (G) at 21 days post-adoptive CAR T-cell transfer.
H Colons at 21 days post-CAR T-cell transfer were fixed in formalin and processed for histology (haematoxylin and eosin) monitoring for tissue architecture and lymphocytic infiltrates. Scale bars: 100 μm.

Data information: Representative results (means ± SEM) are shown from two independent experiments. In (B, F), significance was determined using 2-tailed Mann–Whitney *U*-test. $*P < 0.05$, $**P < 0.01$.

 © 2019 The Authors

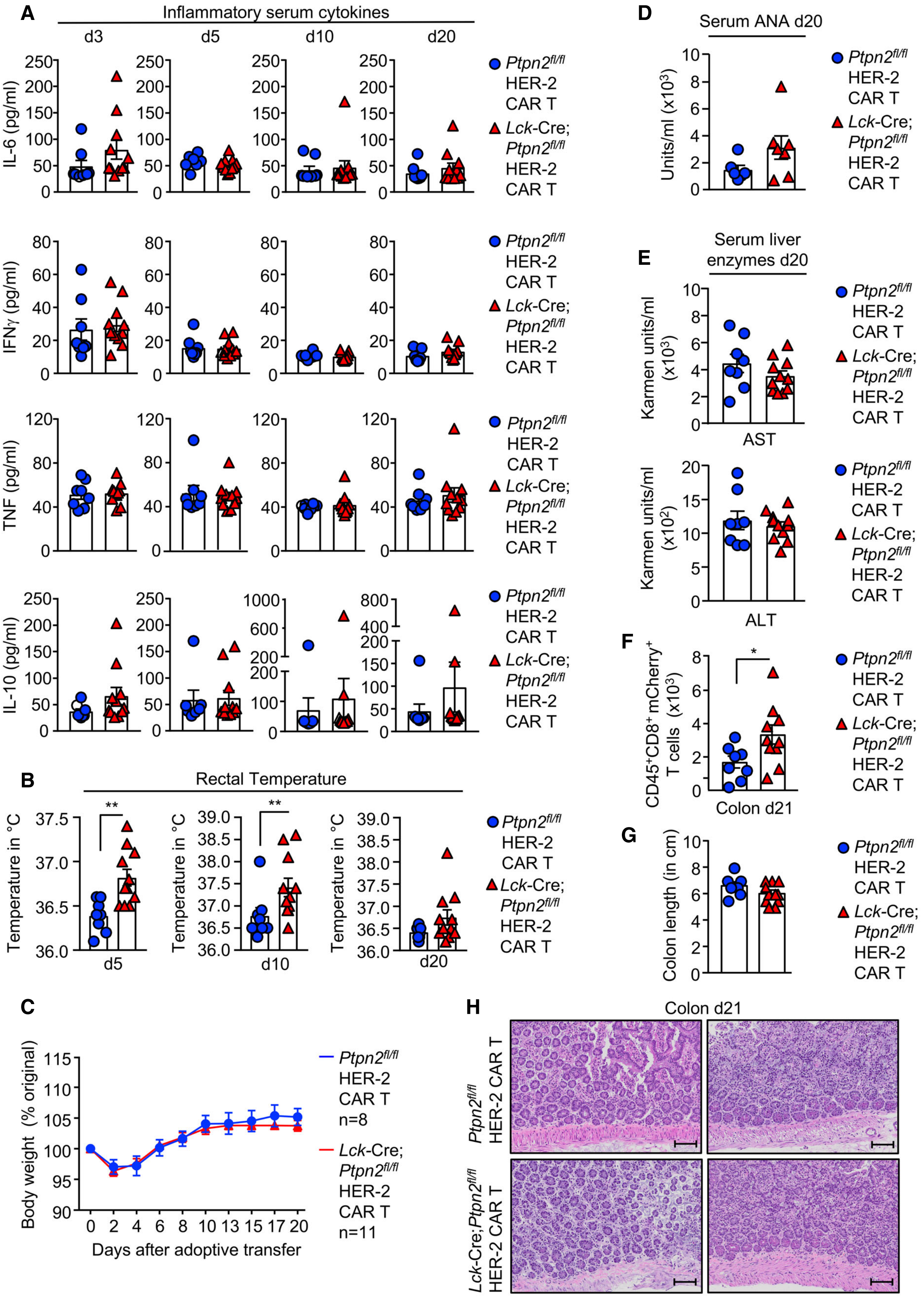
A
Inflammatory serum cytokines
d3
d5
d10
d20
IL-6 (pg/ml)
IFNγ (pg/ml)
TNF (pg/ml)
IL-10 (pg/ml)
Ptpn2fl/fl HER-2 CAR T
Lck-Cre; Ptpn2fl/fl HER-2 CAR T
B
Rectal Temperature
Temperature in °C
d5
d10
d20
C
Body weight (% original)
Days after adoptive transfer
Ptpn2fl/fl HER-2 CAR T n=8
Lck-Cre; Ptpn2fl/fl HER-2 CAR T n=11
D
Serum ANA d20
Units/ml (x10³)
E
Serum liver enzymes d20
Karmen units/ml (x10³)
AST
Karmen units/ml (x10²)
ALT
F
CD45+CD8+ mCherry+ T cells (x10³)
Colon d21
G
Colon length (in cm)
H
Colon d21
Ptpn2fl/fl HER-2 CAR T
Lck-Cre;Ptpn2fl/fl HER-2 CAR T

Figure EV4.

Figure EV5. Targeting Ptpn2 with siSTABLE™ siRNAs or using CRISPR-Cas9 RNP enhances tumour-specific CAR T-cell responses.

A *Ptpn2*$^{fl/fl}$ versus *Lck*-Cre;*Ptpn2*$^{fl/fl}$ CD8$^+$ HER-2 CAR T cells were treated with PTPN2-inhibitor (+) or vehicle (–) followed by incubation with 24JK-HER-2 versus 24JK sarcoma cells. The proportion of CD8$^+$IFNγ$^+$ versus CD8$^+$IFNγ$^+$TNF$^+$ CAR T cells was determined by flow cytometry.

B Human PBMCs were pretreated with PTPN2-inhibitor (+) or vehicle (–) and stimulated with α-CD3. MFIs for CD69 on CD8$^+$CCR7$^+$CD45RA$^+$ T cells and CD8$^+$CCR7$^+$CD45RA$^+$ T-cell numbers were determined by flow cytometry.

C HER-2 CAR T cells transfected with GFP versus *Ptpn2* siSTABLE™ siRNAs were incubated with 24JK-HER-2 versus 24JK sarcoma cells. CD44, CD25, PD-1 and LAG-3 MFIs on CD8$^+$ CAR T cells were determined by flow cytometry.

D HER-2 CAR T cells transfected with GFP versus *Ptpn2* siSTABLE™ siRNAs were incubated with 24JK-HER-2 versus 24JK sarcoma cells. The proportion of CD8$^+$IFNγ$^+$ CAR T cells was determined by flow cytometry.

E HER-2 CAR T cells transfected with GFP versus *Ptpn2* siSTABLE™ siRNAs were incubated with 5 μM CTV-labelled (CTV^{bright}) 24JK-HER-2 and 0.5 μM CTV-labelled (CTV^{dim}) 24JK sarcoma cells. Antigen-specific target cell lysis (24JK-HER-2 versus 24JK response) was monitored for the depletion of CTV^{bright} 24JK-HER-2 cells by flow cytometry.

F mCherry$^+$ CAR T cells isolated from HER-2-E0771 tumours 21 days post-adoptive transfer were assessed for the proportion of CD8$^+$IFNγ$^+$ versus CD8$^+$IFNγ$^+$TNF$^+$ CAR T cells by flow cytometry.

G, H HER-2 CAR T cells were transfected with control versus *Ptpn2* sgRNAs plus Cas9 using the Lonza 4D-Nucleofector to delete PTPN2 by CRISPR-Cas9 RNP. (G) Control and PTPN2-deleted HER-2 CAR T cells were incubated with 24JK-HER-2 versus 24JK sarcoma cells and the proportion of CD8$^+$IFNγ$^+$ CAR T cells determined by flow cytometry. (H) Alternatively, FACS-purified CD8$^+$CD44^{hi}CD62L^{hi} CAR T cells and CD8$^+$CD44^{hi}CD62L^{lo} CAR T control and PTPN2-deleted HER-2 CAR T cells were incubated with 5 μM CTV-labelled (CTV^{bright}) 24JK-HER-2 cells and 0.5 μM CTV-labelled (CTV^{dim}) 24JK sarcoma cells. Antigen-specific target cell lysis (24JK-HER-2 versus 24JK response) was assessed by monitoring for the depletion of CTV^{bright} 24JK-HER-2 cells by flow cytometry.

Data information: Representative results (means ± SEM) from at least two independent experiments are shown. In (A–D, F, G), significance was determined using 2-tailed Mann–Whitney *U*-test. In (E, H), significance was determined using 2-way ANOVA test. *$P < 0.05$, **$P < 0.01$, ***$P < 0.001$, ****$P < 0.0001$.

© 2019 The Authors

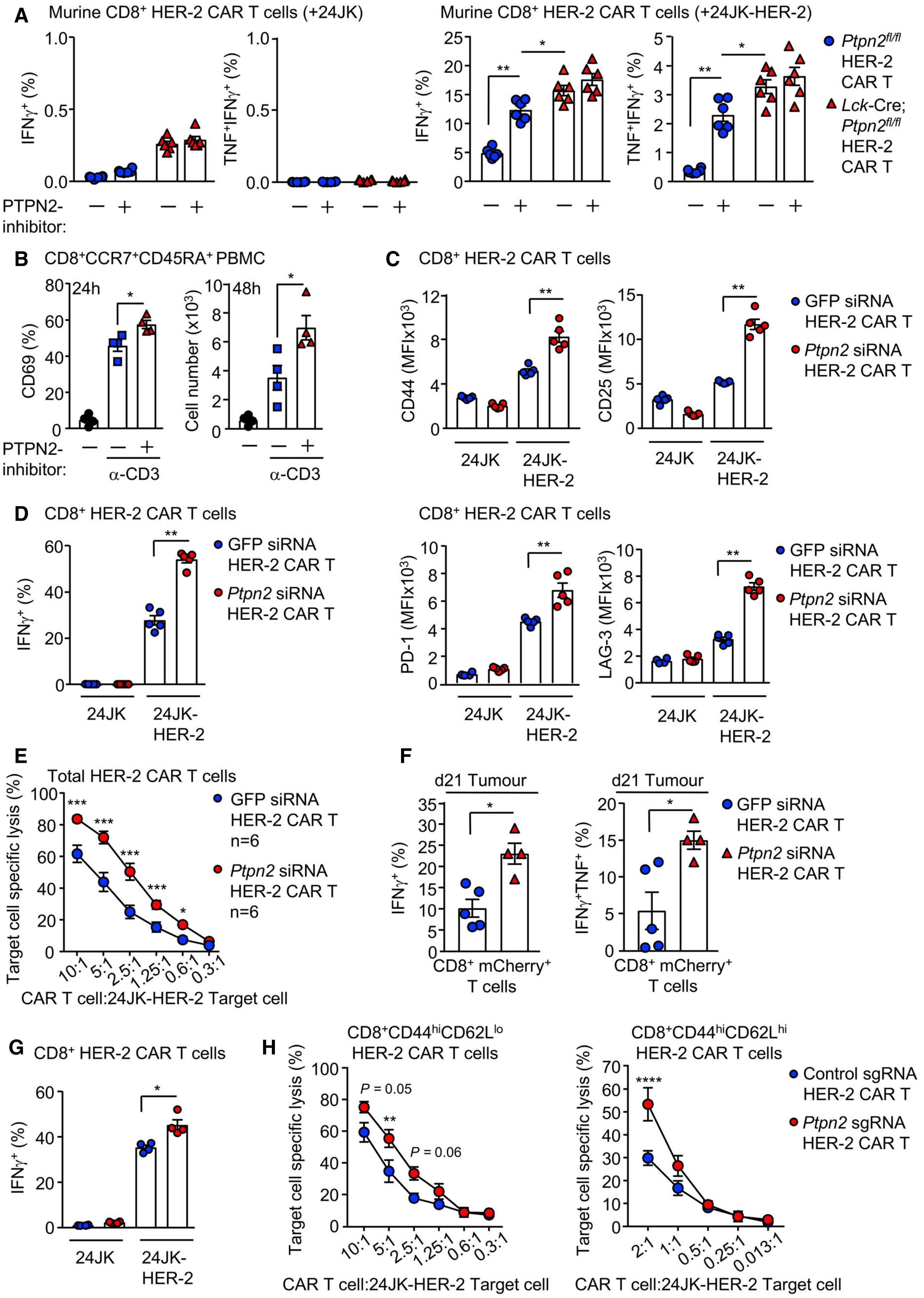
A
Murine CD8+ HER-2 CAR T cells (+24JK)
Murine CD8+ HER-2 CAR T cells (+24JK-HER-2)
IFNγ+ (%)
TNF+IFNγ+ (%)
PTPN2-inhibitor:
Ptpn2fl/fl HER-2 CAR T
Lck-Cre; Ptpn2fl/fl HER-2 CAR T
B
CD8+CCR7+CD45RA+ PBMC
24h
48h
CD69 (%)
Cell number (x10³)
α-CD3
C
CD8+ HER-2 CAR T cells
CD44 (MFIx10³)
CD25 (MFIx10³)
24JK
24JK-HER-2
GFP siRNA HER-2 CAR T
Ptpn2 siRNA HER-2 CAR T
D
CD8+ HER-2 CAR T cells
PD-1 (MFIx10³)
LAG-3 (MFIx10³)
E
Total HER-2 CAR T cells
Target cell specific lysis (%)
GFP siRNA HER-2 CAR T n=6
Ptpn2 siRNA HER-2 CAR T n=6
CAR T cell:24JK-HER-2 Target cell
F
d21 Tumour
IFNγ+TNF+ (%)
CD8+ mCherry+ T cells
G
H
CD8+CD44hiCD62Llo HER-2 CAR T cells
CD8+CD44hiCD62Lhi HER-2 CAR T cells
P = 0.05
P = 0.06
Control sgRNA HER-2 CAR T
Ptpn2 sgRNA HER-2 CAR T

Figure EV5.
