# Supplementary figures and images for: PTPN2 phosphatase deletion in T cells promotes anti‐tumour immunity and CAR T‐cell efficacy in solid tumours
Source: EMBO J. 2019 Dec 5;39(2):e103637. doi: 10.15252/embj.2019103637 (PMC6960448; doi:10.15252/embj.2019103637)

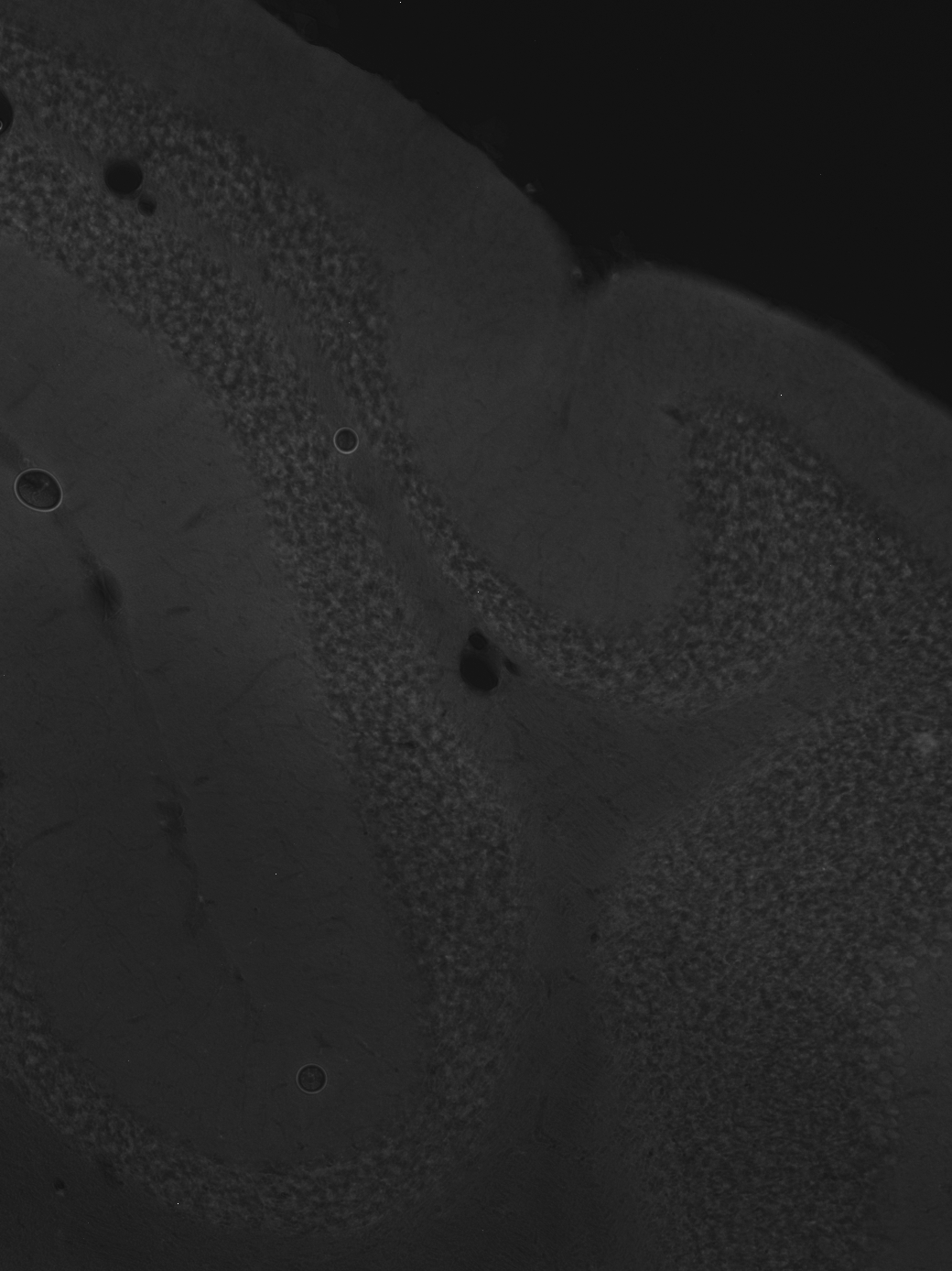

Supplement: Supplementary file 3 — Source Data for Appendix [file EMBJ-39-e103637-s003.zip › Appendix_Figure_S_6C_Source_Data/WT_CD3_crus_1_ansiform_lobe.tiff]
