# Supplementary material for: PTPN2 phosphatase deletion in T cells promotes anti‐tumour immunity and CAR T‐cell efficacy in solid tumours
Source: EMBO J. 2019 Dec 5;39(2):e103637. doi: 10.15252/embj.2019103637 (PMC6960448; doi:10.15252/embj.2019103637)
Supplement: Supplementary file 4 — Review Process File [file EMBJ-39-e103637-s004.pdf]

THE
EMBO
JOURNAL

# PTPN2 phosphatase deletion in T cells promotes anti-tumour immunity and CAR-T cell efficacy in solid tumours

Florian Wiede, Kun-Hui Lu, Xin Du, Shuwei Liang, Katharina Hochheiser, Garron T. Dodd, Pei K. Goh, Conor Kearney, Deborah Meyran, Paul A. Beavis, Melissa A. Henderson, Simone L. Park, Jason Waithman, Sheng Zhang, Zhong-Yin Zhang, Jane Oliaro, Thomas Gebhardt, Phillip K. Darcy, and Tony Tiganis

**Review timeline:**

| | |
|---|---|
| Submission date: | 4th Oct 2019 |
| Accepted: | 8th Nov 2019 |

Editor: Daniel Klimmeck

**Transaction Report:**

(No Peer Review Process File is available with this article, as the authors have chosen not to make the review process public in this case.)

© European Molecular Biology Organization 

EMBO PRESS

YOU MUST COMPLETE ALL CELLS WITH A PINK BACKGROUND ➔

PLEASE NOTE THAT THIS CHECKLIST WILL BE PUBLISHED ALONGSIDE YOUR PAPER


Corresponding Author Name: Tony Tiganis



Journal Submitted to: EMBO J

Manuscript Number: EMBOJ-2019-103637R


### Reporting Checklist For Life Sciences Articles (Rev. June 2017)

This checklist is used to ensure good reporting standards and to improve the reproducibility of published results. These guidelines are consistent with the Principles and Guidelines for Reporting Preclinical Research issued by the NIH in 2014. Please follow the journal's authorship guidelines in preparing your manuscript.

### A- Figures

### 1. Data

**The data shown in figures should satisfy the following conditions:**

- ➔ the data were obtained and processed according to the field's best practice and are presented to reflect the results of the experiments in an accurate and unbiased manner.
- ➔ figure panels include only data points, measurements or observations that can be compared to each other in a scientifically meaningful way.
- ➔ graphs include clearly labeled error bars for independent experiments and sample sizes. Unless justified, error bars should not be shown for technical replicates.
- ➔ if n< 5, the individual data points from each experiment should be plotted and any statistical test employed should be justified
- ➔ Source Data should be included to report the data underlying graphs. Please follow the guidelines set out in the author ship guidelines on Data Presentation.

### 2. Captions

**Each figure caption should contain the following information, for each panel where they are relevant:**

- ➔ a specification of the experimental system investigated (eg cell line, species name).
- ➔ the assay(s) and method(s) used to carry out the reported observations and measurements
- ➔ an explicit mention of the biological and chemical entity(ies) that are being measured.
- ➔ an explicit mention of the biological and chemical entity(ies) that are altered/varied/perturbed in a controlled manner.

- ➔ the exact sample size (n) for each experimental group/condition, given as a number, not a range;
- ➔ a description of the sample collection allowing the reader to understand whether the samples represent technical or biological replicates (including how many animals, litters, cultures, etc.).
- ➔ a statement of how many times the experiment shown was independently replicated in the laboratory.
- ➔ definitions of statistical methods and measures:
  - common tests, such as t-test (please specify whether paired vs. unpaired), simple χ2 tests, Wilcoxon and Mann-Whitney tests, can be unambiguously identified by name only, but more complex techniques should be described in the methods section;
  - are tests one-sided or two-sided?
  - are there adjustments for multiple comparisons?
  - exact statistical test results, e.g., P values = x but not P values < x;
  - definition of 'center values' as median or average;
  - definition of error bars as s.d. or s.e.m.

Any descriptions too long for the figure legend should be included in the methods section and/or with the source data.

**In the pink boxes below, please ensure that the answers to the following questions are reported in the manuscript itself. Every question should be answered. If the question is not relevant to your research, please write NA (non applicable). We encourage you to include a specific subsection in the methods section for statistics, reagents, animal models and human subjects.**

### USEFUL LINKS FOR COMPLETING THIS FORM

| | |
|---|---|
| http://www.antibodypedia.com | Antibodypedia |
| http://1degreebio.org | 1DegreeBio |
| http://www.equator-network.org/reporting-guidelines/improving-bioscience-research-repo | ARRIVE Guidelines |
| http://grants.nih.gov/grants/olaw/olaw.htm | NIH Guidelines in animal use |
| http://www.mrc.ac.uk/Ourresearch/Ethicsresearchguidance/Useofanimals/index.htm | MRC Guidelines on animal use |
| http://ClinicalTrials.gov | Clinical Trial registration |
| http://www.consort-statement.org | CONSORT Flow Diagram |
| http://www.consort-statement.org/checklists/view/32-consort/66-title | CONSORT Check List |
| http://www.equator-network.org/reporting-guidelines/reporting-recommendations-for-tun | REMARK Reporting Guidelines (marker prognostic studies) |
| http://datadryad.org | Dryad |
| http://figshare.com | Figshare |
| http://www.ncbi.nlm.nih.gov/gap | dbGAP |
| http://www.ebi.ac.uk/ega | EGA |
| http://biomodels.net/ | Biomodels Database |
| http://biomodels.net/miriam/ | MIRIAM Guidelines |
| http://jjj.biochem.sun.ac.za | JWS Online |
| http://oba.od.nih.gov/biosecurity/biosecurity_documents.html | Biosecurity Documents from NIH |
| http://www.selectagents.gov/ | List of Select Agents |

### B- Statistics and general methods

| | Please fill out these boxes ➔ (Do not worry if you cannot see all your text once you press return) |
|---|---|
| 1.a. How was the sample size chosen to ensure adequate power to detect a pre-specified effect size? | For in vitro studies sample size was chosen based on common practice and previous experience. Hence, sample size was not calculated on power calculations. However, we have consistently aimed for samples sizes at least > 4, which ensures the correct application of a non-parametric t-test. |
| 1.b. For animal studies, include a statement about sample size estimate even if no statistical methods were used. | For in vivo studies the sample size was generally not predefined before each individual experiment. There were several limiting factors in this study (e.g. availability of experimental female HER-2 mice and frequency of FACS-purified CAR T cells) why sample-size calculation was not performed: Homozygous over-expression of human HER-2 has negative implications on mating productivity hence only wild-type with heterozygous HER-2 mice were mated which lowered the frequency of experimental HER-2+ mice to 25% per litter (in addition, only females could be used for the E0771 orthotopic breast cancer model). For all our ACT experiments we have used FACS-purified central/memory CAR T cells or FACS-purified naive OT-1 T cells. Sorting efficiency and sorting time were two critical factors which detemined the number of tumuor-bearing recipients that could be used. |
| 2. Describe inclusion/exclusion criteria if samples or animals were excluded from the analysis. Were the criteria pre-established? | For in vitro studies no data exclusions were undertaken in this manuscript with the exception in Supplemental Figure 2a, where one data point for the IL-6 measurement in the Lck-Cre;Ptpn2fl/fl group was removed with the Grubb-outlier test using Graphpad Prism 7.0b. For in vivo studies, exclusion criteria were defined by the experimental protocol approved by the AECC. |
| 3. Were any steps taken to minimize the effects of subjective bias when allocating animals/samples to treatment (e.g. randomization procedure)? If yes, please describe. | NA |
| For animal studies, include a statement about randomization even if no randomization was used. | Tumour-bearing mice were not randomized before adoptive T cell therapy but rather evenly distributed according to tumour size between the non-treated group, the WT T cell recipients and the PTPN2 KO T cell recipients This ensured equal tumour sizes between the different groups at the start of every experiment. |
| 4.a. Were any steps taken to minimize the effects of subjective bias during group allocation or/and when assessing results (e.g. blinding of the investigator)? If yes please describe. | NA |
| 4.b. For animal studies, include a statement about blinding even if no blinding was done | Where possible tumour measurements were performed double-blinded. |
| 5. For every figure, are statistical tests justified as appropriate? | We have included a statment at the end of every figure legend, which describes the stastical test for every experiment. |
| Do the data meet the assumptions of the tests (e.g., normal distribution)? Describe any methods used to assess it. | The statistical tests were chosen based on the underlying distribution. In cases where normal distribution could not be assumed, non-parametric t-tests were used. |
| Is there an estimate of variation within each group of data? | Yes |
| Is the variance similar between the groups that are being statistically compared? | Yes |

### C- Reagents

| | |
|---|---|
| 6. To show that antibodies were profiled for use in the system under study (assay and species), provide a citation, catalog number and/or clone number, supplementary information or reference to an antibody validation profile. e.g., Antibodypedia (see link list at top right), 1DegreeBio (see link list at top right). | The following antibodies raised agains mouse from BD Biosciences, BioLegend or eBioscience were used for flow cytometry: Phycoerythrin (PE) or peridinin-chlorophyll cyanine 5.5 (PerCP-Cy5.5)-conjugated CD3 (145-2C11); PerCP-Cy5.5 or phycoerythrin-cyanine 7 (PE-Cy7)-conjugated CD4 (RM4-5); BV711, Pacific Blue-conjugated (PB), allophycocyanin (APC)-Cy7 or APC-conjugated CD8 (53-6.7); PerCP-Cy5.5, PE or APC-Cy7-conjugated CD25 (P61); Fluorescein isothiocyanate (FITC), V450, AlexaFluor 700 or PE-Cy7-conjugated CD44 (IM7); APC-conjugated CD45 (30-F11); V450, APC, AlexaFluor 488 or PE-conjugated CD45.1 (Ly5.1; A20); FITC, PB or PerCP-Cy5.5-conjugated CD45.2 (Ly5.2; 104); APC-conjugated CD45R (B220; RA3-6B2); PE-Cy7, APC or PE-conjugated CD62L (Mel-14); PE-conjugated CD122 (TM-β1); PE-conjugated CD132 (4G3); AlexaFluor 647-conjugated CD183 (CXCR3-173); PerCP-eFluo710-conjugated CD185 (CXCR5; SPRCL5); PE-Cy7 conjugated CD197 (CCR7; 4B12); PE-conjugated CD223 (LAG-3; C9B7W); PE-Cy7-conjugated CD279 (PD-1, RMP1-14); FITC-conjugated CD11b (M1/70); APC-Cy7-conjugated Ly6C (AL-21); PE-conjugated Ly-6G (clone 1A8), APC-conjugated F4/80 (BM8); PE-conjugated IL-10 (JES5-16E3), PE-conjugated Nur77 (12.14), PE-Cy7-conjugated IFN-g (XMG1.2); FITC or APC-conjugated TNF (MP6-XT22); AlexaFluor 647-conjugated Granzyme B (GB11), PE-Cy7-conjugated TCR-Va2 (B20.1); eFluor 660-conjugated p(Y418)-Src (SC1T2M3); AlexaFluor 647-conjugated T-bet (4B10), V450-conjugated FoxP3 (clone MF23).<br>The following antibodies against human T cells were used for flow cytometry: PE-conjugated CD197 (CCR7; REA108; Miltenyi Biotec); VioBright FITC-conjugated CD8 (BW135/80; Miltenyi Biotec); VioBlue-conjugated CD45RA (REA1047, Miltenyi Biotec), AlexaFlour 647-conjugated anti-3S193 idiotype [supplied by Ludwig Institute for Cancer Research (LICR)], PE-conjugated anti-human CD34 (BioLegend), BV785-conjugated anti-human CD3 (UCHT1, BioLegend), BV605-conjugated anti-human CD4 (OKT-4, BioLegend) and PE-Cy7-conjugated anti-human CD8 (SK1, BioLegend)CD4 (BV785-conjugated anti-human; OKT-4, BioLegend), CD8 (APC-H7-conjugated anti-human; SK1, BD Bioscience), IFN-g (PE-Cy7-conjugated anti-human; B27, BioLegend) and TNF (APC-conjugated anti-human TNF; MAb11; BioLegend). For cell stimulation, anti-CD3e (clone 145-2C11), anti-CD28 (clone 37.51) and anti-CD3e (clone OKT-3) antibodies were purchased from BD Biosciences. Antibodies against p-(Y701) STAT1 (clone 58D6), p-(Y694) STAT5 (D47E7) XP® and STAT1 were from Cell Signaling. Anti-Actin (clone ACTN05) was purchased from Thermo Fisher Scientific. The mouse antibody against PTPN2 (clone 6F3) was provided by M. Tremblay (McGill University). Anti-PTPN2 (6F3) was validated by immunoblotting as previously shown by Wiede et al. (Nature Communications, 2014). For immunofluorescence staining and immunohistochemistry rabbit anit-CD3e and mouse anti-HER-2 from Abcam was used. |
| 7. Identify the source of cell lines and report if they were recently authenticated (e.g., by STR profiling) and tested for mycoplasma contamination. | The human epithelial adenocarcinoma cell line OVCAR-3 (ATCC®HTB-161™) and the human melanoma cell line MDA-MB-435 (ATCC®HTB-219™) were obtained from the American Type Culture Collection (ATCC).The C57BL/6 mouse breast carcinoma cell line E0771 were obtained from Robin Anderson (Peter MacCallum Cancer Centre) and the C57BL/6 mouse sarcoma cell line (24-JK) was obtained from Patrick Hwu, NIH, Bethesda, Maryland, USA. Both cells lines have been genetically engineered to express truncated human HER-2 (HER-2-E0771) by Kershaw et al. (2004, Journal of Immunology).<br>The C57BL/6 mouse breast tumour cell line AT-3 was genetically engineered to express chicken ovalbumin (AT-3-OVA) and has been previously described (Loi et al; 2013, PNAS). The GP+E86 packaging line was generated by Darcy et al. (2000, Journal of Immunology) as previously described. |

* for all hyperlinks, please see the table at the top right of the document

## D- Animal Models

| | |
|---|---|
| 8. Report species, strain, gender, age of animals and genetic modification status where applicable. Please detail housing and husbandry conditions and the source of animals. | Mice were maintained on a 12 h light-dark cycle in a temperature-controlled high barrier facility with free access to food and water. All mice used in this study were on the C57BL/6J background. 6-10 week old female Ly5.1 (B6.SJL-PtprcaPepcb/BoyJ) and human HER-2 transgenic (TG) recipient mice and 6-8 week old female donor mice were used for adoptive transfers. For ex-vivo experiments either d 6-8 week old male or female mice were used. Aged- and sex-matched littermates were used in all experiments. Ptpn2fl/fl and Lck-Cre;Ptpn2fl/fl mice and the corresponding OT-1 TCR transgenic mice were described in Wiede et. al (JCI, 2011). p53+/– mice have been described previously (Jacks et al., 1994) and were a gift from Prof. Andreas Strasser (WEHI, Melbourne, Australia). Ptpn2fl/fl;p53+/– and Lck-Cre;Ptpn2fl/fl;p53+/– mice were generated by crossing p53+/– with Lck-Cre;Ptpn2fl/fl mice. B6.129S2-Lck<tm1Mak>/J mice were a gift from Dr Andre Veillette (McGill University, Montreal) and were bred with Lck-Cre;Ptpn2fl/fl mice to generate Lck-Cre;Ptpn2fl/fl;Lck+/– mice. Ly5.1 and C57BL/6 mice were purchased from the WEHI Animal Facility (Kew, Australia) and human HER-2 (C57BL/6) transgenic (TG) mice were bred and maintained at the Peter MacCallum Cancer Centre. |
| 9. For experiments involving live vertebrates, include a statement of compliance with ethical regulations and identify the committee(s) approving the experiments. | All experiments were performed in accordance with the NHMRC Australian Code of Practice for the Care and Use of Animals. All protocols were approved by the Monash University School of Biomedical Sciences Animal Ethics Committee (Ethics number: MARP/2012/124) or the Peter MacCallum Animal Ethics and Experimentation Committee (Ethics numbers: E570, E582 and E604). |
| 10. We recommend consulting the ARRIVE guidelines (see link list at top right) (PLoS Biol. 8(6), e1000412, 2010) to ensure that other relevant aspects of animal studies are adequately reported. See author guidelines, under 'Reporting Guidelines'. See also: NIH (see link list at top right) and MRC (see link list at top right) recommendations. Please confirm compliance. | Animal studies were reported according to the ARRIVE guidelines. |

## E- Human Subjects

| | |
|---|---|
| 11. Identify the committee(s) approving the study protocol. | NA |
| 12. Include a statement confirming that informed consent was obtained from all subjects and that the experiments conformed to the principles set out in the WMA Declaration of Helsinki and the Department of Health and Human Services Belmont Report. | NA |
| 13. For publication of patient photos, include a statement confirming that consent to publish was obtained. | NA |
| 14. Report any restrictions on the availability (and/or on the use) of human data or samples. | NA |
| 15. Report the clinical trial registration number (at ClinicalTrials.gov or equivalent), where applicable. | NA |
| 16. For phase II and III randomized controlled trials, please refer to the CONSORT flow diagram (see link list at top right) and submit the CONSORT checklist (see link list at top right) with your submission. See author guidelines, under 'Reporting Guidelines'. Please confirm you have submitted this list. | NA |
| 17. For tumor marker prognostic studies, we recommend that you follow the REMARK reporting guidelines (see link list at top right). See author guidelines, under 'Reporting Guidelines'. Please confirm you have followed these guidelines. | NA |

## F- Data Accessibility

| | |
|---|---|
| 18: Provide a "Data Availability" section at the end of the Materials & Methods, listing the accession codes for data generated in this study and deposited in a public database (e.g. RNA-Seq data: Gene Expression Omnibus GSE39462, Proteomics data: PRIDE PXD000208 etc.) Please refer to our author guidelines for 'Data Deposition'.<br><br>Data deposition in a public repository is mandatory for:<br>a. Protein, DNA and RNA sequences<br>b. Macromolecular structures<br>c. Crystallographic data for small molecules<br>d. Functional genomics data<br>e. Proteomics and molecular interactions | NA |
| 19. Deposition is strongly recommended for any datasets that are central and integral to the study; please consider the journal's data policy. If no structured public repository exists for a given data type, we encourage the provision of datasets in the manuscript as a Supplementary Document (see author guidelines under 'Expanded View' or in unstructured repositories such as Dryad (see link list at top right) or Figshare (see link list at top right). | NA |
| 20. Access to human clinical and genomic datasets should be provided with as few restrictions as possible while respecting ethical obligations to the patients and relevant medical and legal issues. If practically possible and compatible with the individual consent agreement used in the study, such data should be deposited in one of the major public access-controlled repositories such as dbGAP (see link list at top right) or EGA (see link list at top right). | NA |
| 21. Computational models that are central and integral to a study should be shared without restrictions and provided in a machine-readable form. The relevant accession numbers or links should be provided. When possible, standardized format (SBML, CellML) should be used instead of scripts (e.g. MATLAB). Authors are strongly encouraged to follow the MIRIAM guidelines (see link list at top right) and deposit their model in a public database such as Biomodels (see link list at top right) or JWS Online (see link list at top right). If computer source code is provided with the paper, it should be deposited in a public repository or included in supplementary information. | NA |

## G- Dual use research of concern

| | |
|---|---|
| 22. Could your study fall under dual use research restrictions? Please check biosecurity documents (see link list at top right) and list of select agents and toxins (APHIS/CDC) (see link list at top right). According to our biosecurity guidelines, provide a statement only if it could. | No |
